# WASPAS method and Aczel-Alsina aggregation operators for managing complex interval-valued intuitionistic fuzzy information and their applications in decision-making

**DOI:** 10.7717/peerj-cs.1362

**Published:** 2023-05-11

**Authors:** Haojun Fang, Tahir Mahmood, Zeeshan Ali, Shouzhen Zeng, Yun Jin

**Affiliations:** 1 Wuxi Vocational College of Science and Technology, Wuxi, China; 2 International Islamic University Islamabad, Islamabad, Pakistan; 3 Ningbo University, Ningbo, China; 4 Zhejiang Gongshang University, Hangzhou, China

**Keywords:** Complex interval-valued intuitionistic fuzzy sets, Aczel-Alsina aggregation operators, Decision-making strategy, WASPAS method

## Abstract

Aczel-Alsina t-norm and t-conorm are a valuable and feasible technique to manage ambiguous and inconsistent information because of their dominant characteristics of broad parameter values. The main theme of this analysis is to explore Aczel-Alsina operational laws in the presence of the complex interval-valued intuitionistic fuzzy (CIVIF) set theory. Furthermore, we derive the theory of aggregation frameworks based on Aczel-Alsina operational laws for managing the theory of CIVIF information. The CIVIF Aczel-Alsina weighted averaging (CIVIFAAWA), CIVIF Aczel-Alsina ordered weighted averaging (CIVIFAAOWA), CIVIF Aczel-Alsina hybrid averaging (CIVIFAAHA), CIVIF Aczel-Alsina weighted geometric (CIVIFAAWG), CIVIF Aczel-Alsina ordered weighted geometric (CIVIFAAOWG) and CIVIF Aczel-Alsina hybrid geometric (CIVIFAAHG) operators are proposed, and their well-known properties and particular cases are also detailly derived. Further, we derive the theory of the WASPAS method for CIVIF information and evaluate their positive and negative aspects. Additionally, we demonstrate the multi-attribute decision-making (MADM) strategy under the invented works. Finally, we express the supremacy and dominancy of the invented methods with the help of sensitive analysis and geometrical shown of the explored works.

## Introduction

MADM strategies aims to identify the best of a few relative other options or positioning choices as per their importance as far as the assessed objective. The techniques are utilized for choosing the most acceptable other option/arrangement, because there is no such option for which all rules’ esteems are awesome. MADM strategy is the sub-part of the decision-making technique that has been used in the region of discrete fields. However, it is massively difficult to apply the MADM technique to the phenomena of fuzzy sets rather than crisp sets. To achieve this idea in the real scenario, Zadeh (1965) explored the fuzzy set (FS), which only depends on the supporting grade (SG) $\overline{\overline{{\mathcal{M}}_{\overline{\overline{{\mathfrak{C}}_{C}}}}}}\in \left[ 0,1 \right] $.

In facilitating that sort of situation, FS suffers from an obvious deficiency for not describing the data in the shape of yes or no, not addressing expert opinion, namely, non-SG (NSG). To conquer this imperfection, Atanassov (1986) proposed the methodology of intuitionistic FS (IFS) with an SG and NSG. The well-known prominent of IFS is as followed: $0\leq \overline{\overline{{\mathcal{M}}_{\overline{\overline{R}}}}}+\overline{\overline{{\mathcal{N}}_{\overline{R}}}}\leq 1$. Interval-valued (IV) data is mostly utilized to depict the ambiguity and problematic occurrences, like the difference in temperature, the vacillation of stock cost, and the scope of circulatory strain. Besides, the IV information might be gotten from various areas or sources. For this, the IV intuitionistic FS (IVIFS), was stated by Atanassov & Gargov (1989) with SG $ \left[ {\overline{\overline{{\mathcal{M}}_{\overline{\overline{R}}}}}}^{L} \left( \widetilde {{x}_{E}} \right) ,{\overline{\overline{{\mathcal{M}}_{\overline{\overline{R}}}}}}^{U} \left( \widetilde {{x}_{E}} \right) \right] $ and NSG $ \left[ {\overline{\overline{{\mathcal{N}}_{\overline{\overline{R}}}}}}^{L} \left( \widetilde {{x}_{E}} \right) ,{\overline{\overline{{\mathcal{N}}_{\overline{\overline{R}}}}}}^{U} \left( \widetilde {{x}_{E}} \right) \right] $ such that $0\leq {\overline{\overline{{\mathcal{M}}_{\overline{\overline{R}}}}}}^{U} \left( \widetilde {{x}_{E}} \right) +{\overline{\overline{{\mathcal{N}}_{\overline{\overline{R}}}}}}^{U} \left( \widetilde {{x}_{E}} \right) \leq 1$.

Based on above discussions, we have obtained the result that the prevailing theories neglect to manage two-domination data in the shape of SG and NSG, and simultaneously neglect to survive with inconsistent and fluctuational at a provided phase of time. However, the data got from “medical research” such that the biometric and facial acknowledgment data set consistently changes with the entry of the time. Along these lines, Ramot et al. (2002) extended the scope of SG from a genuine subset to the unit circle of the complicated plane and henceforth established the principle of complex FS (CFS). The mathematical structure of SG in the circumstances of CFS is of the form $\overline{\overline{{\mathcal{M}}_{\overline{\overline{{\mathfrak{C}}_{C}}}}}} \left( \widetilde {{x}_{E}} \right) =\overline{\overline{{\mathcal{M}}_{\overline{\overline{R}}}}} \left( \widetilde {{x}_{E}} \right) {e}^{i2\pi \left( \overline{\overline{{\mathcal{M}}_{I}}} \left( \widetilde {{x}_{E}} \right) \right) }$ with $\overline{\overline{{\mathcal{M}}_{\overline{\overline{R}}}}} \left( \widetilde {{x}_{E}} \right) ,\overline{\overline{{\mathcal{M}}_{\overline{\overline{I}}}}} \left( \widetilde {{x}_{E}} \right) \in \left[ 0,1 \right] $. Since CFS restricts only up to SG and does not take into account NSG, Alkouri & Salleh (2012) produced the principle of complex IFS (CIFS) in the shape of SG $\overline{\overline{{\mathcal{M}}_{\overline{\overline{{\mathfrak{C}}_{C}}}}}} \left( \widetilde {{x}_{E}} \right) =\overline{\overline{{\mathcal{M}}_{\overline{\overline{R}}}}} \left( \widetilde {{x}_{E}} \right) {e}^{i2\pi \left( \overline{\overline{{\mathcal{M}}_{\overline{\overline{I}}}}} \left( \widetilde {{x}_{E}} \right) \right) }$ and NSG $\overline{\overline{{\mathcal{N}}_{\overline{\overline{{\mathfrak{C}}_{C}}}}}} \left( \widetilde {{x}_{E}} \right) =\overline{\overline{{\mathcal{N}}_{\overline{\overline{R}}}}} \left( \widetilde {{x}_{E}} \right) {e}^{i2\pi \left( \overline{\overline{{\mathcal{N}}_{\overline{\overline{I}}}}} \left( \widetilde {{x}_{E}} \right) \right) }$, with $0\leq \overline{\overline{{\mathcal{M}}_{\overline{\overline{R}}}}} \left( \widetilde {{x}_{E}} \right) +\overline{\overline{{\mathcal{N}}_{\overline{\overline{R}}}}} \left( \widetilde {{x}_{E}} \right) \leq 1$ and $0\leq \overline{\overline{{\mathcal{M}}_{\overline{\overline{I}}}}} \left( \widetilde {{x}_{E}} \right) +\overline{\overline{{\mathcal{N}}_{\overline{\overline{I}}}}} \left( \widetilde {{x}_{E}} \right) \leq 1$. Recently, Garg & Rani (2019a) studied the form of CIFS in the interval environment and proposed the mathematical structure of CIVIFS. Because of its strong ability in dealing with uncertain information, CIVIFS has been promoted in many ways, but the results in Aczel-Alsina operational laws still need to be enriched.

## Literature Review

Under the powerful characteristic of FS, many scholars have conducted a lot of extended research. For illustration, ordered weighted averaging aggregation operators (Yager, 1988), immediate probabilities (Yager, Engemann & Filev, 1995), modeling decision-making under immediate probabilities (Engemann, Filev & Yager, 1996), mixed uncertain satisfaction (Yager, 2017), aggregation function (Durante & Ricci, 2018), deviation-based aggregation (Decký, Mesiar & Stupňanová, 2018), generalized averaging aggregation operators (Beliakov et al., 2011; Liu et al., 2016; Yang & Yao, 2021), and analysis of fuzzy research under bibliometric indicators (Merigó, Gil-Lafuente & Yager, 2015).

Due to strong data-inclusive features, IFS and IVIFS have been extended in distinct regions, including bipolar soft sets (Mahmood, 2020), analysis of image quality under measures (Hassaballah & Ghareeb, 2017), decision-making framework (Gao et al., 2021; Zeng, Hu & Llopis-Albert, 2023), distance and similarity measures (Garg & Rani, 2021; Peng, Xiaohe & Jianbo, 2021), hybrid variable approach (Liu et al., 2021; Xue, Deng & Garg, 2021; Zhang et al., 2022), construction of shadowed sets (Yang & Yao, 2021), time-series mapping (Bas, Yolcu & Egrioglu, 2021), transportation problem (Bharati, 2021), and combined compromise solution approach (Alrasheedi et al., 2021; Su et al., 2023).

Due to its dominant structure, several scholars have shown their interest in CFS and applied it to diverse regions. For convenience, cross-entropy measures (Liu, Ali & Mahmood, 2020), complex fuzzy soft sets (CFSS) (Thirunavukarasu, Suresh & Ashokkumar, 2017), IV CFSS (Selvachandran & Singh, 2018; Dai, Bi & Hu, 2019), and complex multi-fuzzy soft sets (Al-Qudah & Hassan, 2019). Further, Garg & Rani (2019a); Garg & Rani (2020a) invented the CIVIFS and the advanced aggregation operators under CIFS. Garg & Rani (2020b) modified the theory of robust and geometric aggregation operators under CIFS. Garg & Rani (2019b) proposed the methodology of aggregation operators under generalized CIFS. Ali et al. (2021) combined the principle of CIFS and soft set and explored some aggregation operators. Especially, the statistical metrics evaluated by Menger (1942), Einstein aggregation operators for IFS invented by Wang & Liu (2012), Archimedean aggregation operators for IFS stated by Xia, Xu & Zhu (2012), Hamacher aggregation operators for interval-valued IFS presented by Liu (2013), and so on.

## Motivation and Main Contribution

CIVIFS theory is the modified version of the FS, IFS, IVIFS, CFS, and CIFS because of their valuable and dominant structure. Further, the theory of Aczel-Alsina is also very famous and reliable because it is the generation of the algebraic t-norm and t-conorm. Moreover, discovering the theory of aggregation operators in the presence of Aczel-Alsina information for managing CIVIF values is a very challenging task for new fuzzy scholars, because up to date no one can derive the theory of Aczel-Alsina aggregation operators for CIVIF values. Furthermore, deriving the theory of the WASPAS technique (Zavadskas et al., 2012) is also a very awkward and challenging task for fuzzy researchers.

Keeping the benefits of the above prevailing operators, the major contribution of this analysis is illustrated below:

(1) To initiate the Aczel-Alsina operational laws and their related results.
(2) To invent the principle of CIVIFAAWA, CIVIFAAOWA, CIVIFAAHA, CIVIFAAWG, CIVIFAAOWG, and CIVIFAAHG operators, and illustrated their well-known properties and results.
(3) To derive the theory of the WASPAS method for CIVIFSs.
(4) To demonstrate the MADM strategy under the invented works.
(5) To express the supremacy and dominancy of the invented works with the help of sensitive analysis and geometrical shown of the explored works.

Presentation of our analysis is implemented in the shape: Section 2 covers all the prevailing methodologies. In Section 3, we initiate the Aczel-Alsina operational laws and their related results. Section 4 produces the principle of CIVIFAAWA, CIVIFAAOWA, CIVIFAAHA, CIVIFAAWG, CIVIFAAOWG, and CIVIFAAHG operators, and illustrates their well-known properties. In Section 5, we derive the WASPAS method for CIVIFSs. In Section 6, we demonstrate the effectiveness of the MADM strategy under the invented works. The conclusion of this study is illustrated in Section 7.

Before starting the proposed work, all variables and indexes used in this study are defined in Table 1.

**Table 1:** Representation of the notation used in the proposed work.

Notation	meanings	Notation	meanings	Notation	Meanings
$\overline{\overline{{\mathfrak{C}}_{{C}_{ij}}}}$	Entry of matrix	$\overline{\overline{{\mathcal{M}}_{\overline{\overline{{\mathfrak{C}}_{C}}}}}} \left( \widetilde {{x}_{E}} \right) $	Complex Interval-valued truth grade	$\overline{\overline{{\mathcal{N}}_{\overline{\overline{{\mathfrak{C}}_{C}}}}}} \left( \widetilde {{x}_{E}} \right) $	Complex Interval-valued falsity grade
***W***_***j***_	Weight vector	${\overline{\overline{{\mathcal{M}}_{\overline{\overline{R}}}}}}^{L} \left( \widetilde {{x}_{E}} \right) $	Lower bound of real part in truth grade	${\overline{\overline{{\mathcal{N}}_{\overline{\overline{R}}}}}}^{L} \left( \widetilde {{x}_{E}} \right) $	Lower bound of real part in falsity grade
***S***_***i***_	Score value	${\overline{\overline{{\mathcal{M}}_{\overline{\overline{R}}}}}}^{U} \left( \widetilde {{x}_{E}} \right) $	upper bound of real part in truth grade	${\overline{\overline{{\mathcal{N}}_{\overline{\overline{R}}}}}}^{U} \left( \widetilde {{x}_{E}} \right) $	upper bound of real part in falsity grade
°F	Scaler	${\overline{\overline{{\mathcal{M}}_{\overline{\overline{I}}}}}}^{L} \left( \widetilde {{x}_{E}} \right) $	Lower bound of imaginary part in truth grade	${\overline{\overline{{\mathcal{N}}_{\overline{\overline{I}}}}}}^{L} \left( \widetilde {{x}_{E}} \right) $	Lower bound of imaginary part in falsity grade
$\widetilde {{\mathbb{X}}_{\mathbi{U}}}$	Universal set	${\overline{\overline{{\mathcal{M}}_{\overline{\overline{I}}}}}}^{U} \left( \widetilde {{x}_{E}} \right) $	upper bound of imaginary part in truth grade	${\overline{\overline{{\mathcal{N}}_{\overline{\overline{I}}}}}}^{U} \left( \widetilde {{x}_{E}} \right) $	upper bound of imaginary part in falsity grade
${\overline{\overline{{\mathfrak{R}}_{\overline{\overline{\mathfrak{C}}}}}}}_{\mathbi{C}} \left( \widetilde {{x}_{E}} \right) $	Complex Interval-valued neutral grade	${\overline{\overline{{\mathfrak{R}}_{\overline{\overline{R}}}}}}^{L} \left( \widetilde {{x}_{E}} \right) $	Lower bound of real part in neutral grade	${\overline{\overline{{\mathfrak{R}}_{\overline{\overline{I}}}}}}^{L} \left( \widetilde {{x}_{E}} \right) $	Lower bound of imaginary part in neutral grade
$\widetilde {{\mathfrak{x}}_{\mathbi{E}}}$	Element of universal set	${\overline{\overline{{\mathfrak{R}}_{\overline{\overline{R}}}}}}^{U} \left( \widetilde {{x}_{E}} \right) $	upper bound of real part in neutral grade	${\overline{\overline{{\mathfrak{R}}_{\overline{\overline{I}}}}}}^{U} \left( \widetilde {{x}_{E}} \right) $	upper bound of imaginary part in neutral grade

## Preliminaries

Here, we utilized the weighted sum model (WSM) and the weighted product model (WPM) to review the concept of the WASPAS method (Zavadskas et al., 2012). Moreover, the extended WASPAS method was derived from Zavadskas et al. (2013). Some valuable and effective steps of the WASPAS method are listed below:

***Step 1:*** The input data of the technique is represented in the form of a matrix of alternatives and attributes, which is based on the data received from the expert.

***Step 2:*** Normalize the decision matrix in the presence of the information in Eq. (1):

(1) \begin{eqnarray*}{\overline{\overline{{\mathfrak{C}}_{{C}_{ij}}}}}^{{}^{{^{\prime}}}}= \left\{ \begin{array}{@{}cc@{}} \displaystyle \frac{\overline{\overline{{\mathfrak{C}}_{{C}_{ij}}}}}{{\max }_{i}\overline{\overline{{\mathfrak{C}}_{{C}_{ij}}}}~} &\displaystyle if~i\in B\\ \displaystyle \frac{{\min }_{i}\overline{\overline{{\mathfrak{C}}_{{C}_{ij}}}}}{\overline{\overline{{\mathfrak{C}}_{{C}_{ij}}}}} &\displaystyle if~i\in C \end{array} \right. \end{eqnarray*}

where *B* represented the benefit types of data and *C* stated the cost type of criteria.

***Step 3:*** Compute WSM and WPM of each alternative:

(2) \begin{eqnarray*}WS{M}_{i}=\sum _{j=1}^{m}{W}_{j}{\overline{\overline{{\mathfrak{C}}_{{C}_{ij}}}}}^{{}^{{^{\prime}}}}\end{eqnarray*}

(3) \begin{eqnarray*}WP{M}_{i}=\prod _{j=1}^{m}{ \left( {\overline{\overline{{\mathfrak{C}}_{{C}_{ij}}}}}^{{}^{{^{\prime}}}} \right) }^{{W}_{j}}\end{eqnarray*}

***Step 4:*** Calculate the score value by using the theory of WSM and WPM information referring to the following way:

(4) \begin{eqnarray*}{S}_{i}=\textdegree F\ast WS{M}_{i}+ \left( 1-\textdegree F \right) WP{M}_{i}.\end{eqnarray*}

There exist some special cases: when °*F* = 1 in Eq. (4), *S*_*i*_ = *WSM*_*i*_; when °*F* = 0 , *S*_*i*_ = *WPM*_*i*_.

***Step 5:*** Deriving the best preference by the score value in Step 4.

Next, the algebraic theories of some prevailing principles like CIVIFSs, the concept of Aczel-Alsina t-norm and t-conorm will be discussed. Of note, the notation $\widetilde {{\mathbb{X}}_{U}}$, stated for universal sets.

**Definition 1:**(Garg & Rani, 2019a) The mathematical structure of CIVIFS $\overline{\overline{{\mathfrak{C}}_{C}}}$ is shown in the shape of:

(5) \begin{eqnarray*}\overline{\overline{{\mathfrak{C}}_{C}}}= \left\{ \left( \overline{\overline{{\mathcal{M}}_{\overline{\overline{{\mathfrak{C}}_{C}}}}}} \left( \widetilde {{x}_{E}} \right) ,\overline{\overline{{\mathcal{N}}_{\overline{\overline{{\mathfrak{C}}_{C}}}}}} \left( \widetilde {{x}_{E}} \right) \right) :\widetilde {{x}_{E}}\in \widetilde {{\mathbb{X}}_{U}} \right\} \end{eqnarray*}

where the term $\overline{\overline{{\mathcal{M}}_{\overline{\overline{{\mathfrak{C}}_{C}}}}}} \left( \widetilde {{x}_{E}} \right) = \left[ {\overline{\overline{{\mathcal{M}}_{\overline{\overline{R}}}}}}^{L} \left( \widetilde {{x}_{E}} \right) ,{\overline{\overline{{\mathcal{M}}_{\overline{\overline{R}}}}}}^{U} \left( \widetilde {{x}_{E}} \right) \right] {e}^{i2\pi \left( \left[ {\overline{\overline{{\mathcal{M}}_{\overline{\overline{I}}}}}}^{L} \left( \widetilde {{x}_{E}} \right) ,{\overline{\overline{{\mathcal{M}}_{\overline{\overline{I}}}}}}^{U} \left( \widetilde {{x}_{E}} \right) \right] \right) }$ and $\overline{\overline{{\mathcal{N}}_{\overline{\overline{{\mathfrak{C}}_{C}}}}}} \left( \widetilde {{x}_{E}} \right) = \left[ {\overline{\overline{{\mathcal{N}}_{\overline{\overline{R}}}}}}^{L} \left( \widetilde {{x}_{E}} \right) ,{\overline{\overline{{\mathcal{N}}_{\overline{\overline{R}}}}}}^{U} \left( \widetilde {{x}_{E}} \right) \right] {e}^{i2\pi \left( \left[ {\overline{\overline{{\mathcal{N}}_{\overline{\overline{I}}}}}}^{L} \left( \widetilde {{x}_{E}} \right) ,{\overline{\overline{{\mathcal{N}}_{\overline{\overline{I}}}}}}^{U} \left( \widetilde {{x}_{E}} \right) \right] \right) }$ indicate the TD and FD with $0\leq {\overline{\overline{{\mathcal{M}}_{\overline{\overline{R}}}}}}^{U} \left( \widetilde {{x}_{E}} \right) +{\overline{\overline{{\mathcal{N}}_{\overline{\overline{R}}}}}}^{U} \left( \widetilde {{x}_{E}} \right) \leq 1$ and $0\leq {\overline{\overline{{\mathcal{M}}_{\overline{\overline{I}}}}}}^{U} \left( \widetilde {{x}_{E}} \right) +{\overline{\overline{{\mathcal{N}}_{\overline{\overline{I}}}}}}^{U} \left( \widetilde {{x}_{E}} \right) \leq 1$. Moreover, $\overline{\overline{{\mathfrak{R}}_{\overline{\overline{{\mathfrak{C}}_{C}}}}}} \left( \widetilde {{x}_{E}} \right) = \left[ {\overline{\overline{{\mathfrak{R}}_{\overline{\overline{R}}}}}}^{L} \left( \widetilde {{x}_{E}} \right) ,{\overline{\overline{{\mathfrak{R}}_{\overline{\overline{R}}}}}}^{U} \left( \widetilde {{x}_{E}} \right) \right] {e}^{i2\pi \left( \left[ {\overline{\overline{{\mathfrak{R}}_{\overline{\overline{I}}}}}}^{L} \left( \widetilde {{x}_{E}} \right) ,{\overline{\overline{{\mathfrak{R}}_{\overline{\overline{I}}}}}}^{U} \left( \widetilde {{x}_{E}} \right) \right] \right) }= \left[ \left( 1- \left( {\overline{\overline{{\mathcal{M}}_{\overline{\overline{R}}}}}}^{L} \left( \widetilde {{x}_{E}} \right) +{\overline{\overline{{\mathcal{N}}_{\overline{\overline{R}}}}}}^{L} \left( \widetilde {{x}_{E}} \right) \right) \right) , \left( 1- \left( {\overline{\overline{{\mathcal{M}}_{\overline{\overline{R}}}}}}^{U} \left( \widetilde {{x}_{E}} \right) +{\overline{\overline{{\mathcal{N}}_{\overline{\overline{R}}}}}}^{U} \left( \widetilde {{x}_{E}} \right) \right) \right) \right] {e}^{i2\pi \left[ \left( 1- \left( {\overline{\overline{{\mathcal{M}}_{\overline{\overline{I}}}}}}^{L} \left( \widetilde {{x}_{E}} \right) +{\overline{\overline{{\mathcal{N}}_{\overline{\overline{I}}}}}}^{L} \left( \widetilde {{x}_{E}} \right) \right) \right) , \left( 1- \left( {\overline{\overline{{\mathcal{M}}_{\overline{\overline{I}}}}}}^{U} \left( \widetilde {{x}_{E}} \right) +{\overline{\overline{{\mathcal{N}}_{\overline{\overline{I}}}}}}^{U} \left( \widetilde {{x}_{E}} \right) \right) \right) \right] }$ states the neutral grade, and

$\overline{\overline{{\mathfrak{C}}_{{C}_{j}}}}= \left( \left[ {\overline{\overline{{\mathcal{M}}_{\overline{\overline{{R}_{j}}}}}}}^{L} \left( \widetilde {{x}_{E}} \right) ,{\overline{\overline{{\mathcal{M}}_{\overline{\overline{{R}_{j}}}}}}}^{U} \left( \widetilde {{x}_{E}} \right) \right] {e}^{i2\pi \left( \left[ {\overline{\overline{{\mathcal{M}}_{\overline{\overline{{I}_{j}}}}}}}^{L} \left( \widetilde {{x}_{E}} \right) ,{\overline{\overline{{\mathcal{M}}_{\overline{\overline{{I}_{j}}}}}}}^{U} \left( \widetilde {{x}_{E}} \right) \right] \right) }, \right. $

$ \left. \left[ {\overline{\overline{{\mathcal{N}}_{\overline{\overline{{R}_{j}}}}}}}^{L} \left( \widetilde {{x}_{E}} \right) ,{\overline{\overline{{\mathcal{N}}_{\overline{\overline{{R}_{j}}}}}}}^{U} \left( \widetilde {{x}_{E}} \right) \right] {e}^{i2\pi \left( \left[ {\overline{\overline{{\mathcal{N}}_{\overline{\overline{{I}_{j}}}}}}}^{L} \left( \widetilde {{x}_{E}} \right) ,{\overline{\overline{{\mathcal{N}}_{\overline{\overline{{I}_{j}}}}}}}^{U} \left( \widetilde {{x}_{E}} \right) \right] \right) } \right) $ denotes the complex interval-valued intuitionistic fuzzy number (CIVIFN).

**Definition 2:**(Garg & Rani, 2019a) Suppose there are two CIVIFNs

$\overline{\overline{{\mathfrak{C}}_{{C}_{j}}}}= \left( {\scriptsize \begin{array}{@{}c@{}} \displaystyle \left[ {\overline{\overline{{\mathcal{M}}_{\overline{\overline{{R}_{j}}}}}}}^{L} \left( \widetilde {{x}_{E}} \right) ,{\overline{\overline{{\mathcal{M}}_{\overline{\overline{{R}_{j}}}}}}}^{U} \left( \widetilde {{x}_{E}} \right) \right] {e}^{i2\pi \left( \left[ {\overline{\overline{{\mathcal{M}}_{\overline{\overline{{I}_{j}}}}}}}^{L} \left( \widetilde {{x}_{E}} \right) ,{\overline{\overline{{\mathcal{M}}_{\overline{\overline{{I}_{j}}}}}}}^{U} \left( \widetilde {{x}_{E}} \right) \right] \right) },\\ \displaystyle \left[ {\overline{\overline{{\mathcal{N}}_{\overline{\overline{{R}_{j}}}}}}}^{L} \left( \widetilde {{x}_{E}} \right) ,{\overline{\overline{{\mathcal{N}}_{\overline{\overline{{R}_{j}}}}}}}^{U} \left( \widetilde {{x}_{E}} \right) \right] {e}^{i2\pi \left( \left[ {\overline{\overline{{\mathcal{N}}_{\overline{\overline{{I}_{j}}}}}}}^{L} \left( \widetilde {{x}_{E}} \right) ,{\overline{\overline{{\mathcal{N}}_{\overline{\overline{{I}_{j}}}}}}}^{U} \left( \widetilde {{x}_{E}} \right) \right] \right) } \end{array}} \right) ,j=1,2$, then:

(6) \begin{eqnarray*}\overline{\overline{{\mathfrak{C}}_{{C}_{1}}}}\oplus \overline{\overline{{\mathfrak{C}}_{{C}_{2}}}}= \left( \begin{array}{@{}c@{}} \displaystyle \left[ {\overline{\overline{{\mathcal{M}}_{\overline{\overline{{R}_{1}}}}}}}^{L}+{\overline{\overline{{\mathcal{M}}_{\overline{\overline{{R}_{2}}}}}}}^{L}-{\overline{\overline{{\mathcal{M}}_{\overline{\overline{{R}_{1}}}}}}}^{L}{\overline{\overline{{\mathcal{M}}_{\overline{\overline{{R}_{2}}}}}}}^{L},{\overline{\overline{{\mathcal{M}}_{\overline{\overline{{R}_{1}}}}}}}^{U}+{\overline{\overline{{\mathcal{M}}_{\overline{\overline{{R}_{2}}}}}}}^{U}-{\overline{\overline{{\mathcal{M}}_{\overline{\overline{{R}_{1}}}}}}}^{U}{\overline{\overline{{\mathcal{M}}_{\overline{\overline{{R}_{2}}}}}}}^{U} \right] \\ \displaystyle {e}^{i2\pi \left[ {\overline{\overline{{\mathcal{M}}_{\overline{\overline{{I}_{1}}}}}}}^{L}+{\overline{\overline{{\mathcal{M}}_{\overline{\overline{{I}_{2}}}}}}}^{L}-{\overline{\overline{{\mathcal{M}}_{\overline{\overline{{I}_{1}}}}}}}^{L}{\overline{\overline{{\mathcal{M}}_{\overline{\overline{{I}_{2}}}}}}}^{L},{\overline{\overline{{\mathcal{M}}_{\overline{\overline{{I}_{1}}}}}}}^{U}+{\overline{\overline{{\mathcal{M}}_{\overline{\overline{{I}_{2}}}}}}}^{U}-{\overline{\overline{{\mathcal{M}}_{\overline{\overline{{I}_{1}}}}}}}^{U}{\overline{\overline{{\mathcal{M}}_{\overline{\overline{{I}_{2}}}}}}}^{U} \right] },\\ \displaystyle \left[ {\overline{\overline{{\mathcal{N}}_{\overline{\overline{{R}_{1}}}}}}}^{L}{\overline{\overline{{\mathcal{N}}_{\overline{\overline{{R}_{2}}}}}}}^{L},{\overline{\overline{{\mathcal{N}}_{\overline{\overline{{R}_{1}}}}}}}^{U}{\overline{\overline{{\mathcal{N}}_{\overline{\overline{{R}_{2}}}}}}}^{U} \right] {e}^{i2\pi \left[ {\overline{\overline{{\mathcal{N}}_{\overline{\overline{{I}_{1}}}}}}}^{L}{\overline{\overline{{\mathcal{N}}_{\overline{\overline{{I}_{2}}}}}}}^{L},{\overline{\overline{{\mathcal{N}}_{\overline{\overline{{I}_{1}}}}}}}^{U}{\overline{\overline{{\mathcal{N}}_{\overline{\overline{{I}_{2}}}}}}}^{U} \right] } \end{array} \right) \end{eqnarray*}

(7) \begin{eqnarray*}\overline{\overline{{\mathfrak{C}}_{{C}_{1}}}}\otimes \overline{\overline{{\mathfrak{C}}_{{C}_{2}}}}= \left( \begin{array}{@{}c@{}} \displaystyle \left[ {\overline{\overline{{\mathcal{M}}_{\overline{\overline{{R}_{1}}}}}}}^{L}{\overline{\overline{{\mathcal{M}}_{\overline{\overline{{R}_{2}}}}}}}^{L},{\overline{\overline{{\mathcal{M}}_{\overline{\overline{{R}_{1}}}}}}}^{U}{\overline{\overline{{\mathcal{M}}_{\overline{\overline{{R}_{2}}}}}}}^{U} \right] {e}^{i2\pi \left[ {\overline{\overline{{\mathcal{M}}_{\overline{\overline{{I}_{1}}}}}}}^{L}{\overline{\overline{{\mathcal{M}}_{\overline{\overline{{I}_{2}}}}}}}^{L},{\overline{\overline{{\mathcal{M}}_{\overline{\overline{{I}_{1}}}}}}}^{U}{\overline{\overline{{\mathcal{M}}_{\overline{\overline{{I}_{2}}}}}}}^{U} \right] },\\ \displaystyle \left[ {\overline{\overline{{\mathcal{N}}_{\overline{\overline{{R}_{1}}}}}}}^{L}+{\overline{\overline{{\mathcal{N}}_{\overline{\overline{{R}_{2}}}}}}}^{L}-{\overline{\overline{{\mathcal{N}}_{\overline{\overline{{R}_{1}}}}}}}^{L}{\overline{\overline{{\mathcal{N}}_{\overline{\overline{{R}_{2}}}}}}}^{L},{\overline{\overline{{\mathcal{N}}_{\overline{\overline{{R}_{1}}}}}}}^{U}+{\overline{\overline{{\mathcal{N}}_{\overline{\overline{{R}_{2}}}}}}}^{U}-{\overline{\overline{{\mathcal{N}}_{\overline{\overline{{R}_{1}}}}}}}^{U}{\overline{\overline{{\mathcal{N}}_{\overline{\overline{{R}_{2}}}}}}}^{U} \right] \\ \displaystyle {e}^{i2\pi \left[ {\overline{\overline{{\mathcal{N}}_{\overline{\overline{{I}_{1}}}}}}}^{L}+{\overline{\overline{{\mathcal{N}}_{\overline{\overline{{I}_{2}}}}}}}^{L}-{\overline{\overline{{\mathcal{N}}_{\overline{\overline{{I}_{1}}}}}}}^{L}{\overline{\overline{{\mathcal{N}}_{\overline{\overline{{I}_{2}}}}}}}^{L},{\overline{\overline{{\mathcal{N}}_{\overline{\overline{{I}_{1}}}}}}}^{U}+{\overline{\overline{{\mathcal{N}}_{\overline{\overline{{I}_{2}}}}}}}^{U}-{\overline{\overline{{\mathcal{N}}_{\overline{\overline{{I}_{1}}}}}}}^{U}{\overline{\overline{{\mathcal{N}}_{\overline{\overline{{I}_{2}}}}}}}^{U} \right] } \end{array} \right) \end{eqnarray*}

(8) \begin{eqnarray*}\overline{\overline{{\psi }_{S}}}\overline{\overline{{\mathfrak{C}}_{{C}_{1}}}}= \left( \begin{array}{@{}c@{}} \displaystyle \left[ \left( 1-{ \left( 1-{\overline{\overline{{\mathcal{M}}_{\overline{\overline{{R}_{1}}}}}}}^{L} \right) }^{\overline{\overline{{\psi }_{S}}}} \right) , \left( 1-{ \left( 1-{\overline{\overline{{\mathcal{M}}_{\overline{\overline{{R}_{1}}}}}}}^{U} \right) }^{\overline{\overline{{\psi }_{S}}}} \right) \right] {e}^{i2\pi \left[ \left( 1-{ \left( 1-{\overline{\overline{{\mathcal{M}}_{\overline{\overline{{I}_{1}}}}}}}^{L} \right) }^{\overline{\overline{{\psi }_{S}}}} \right) , \left( 1-{ \left( 1-{\overline{\overline{{\mathcal{M}}_{\overline{\overline{{I}_{1}}}}}}}^{U} \right) }^{\overline{\overline{{\psi }_{S}}}} \right) \right] },\\ \displaystyle \left[ {{\overline{\overline{{\mathcal{N}}_{\overline{\overline{{R}_{1}}}}}}}^{L}}^{\overline{{\psi }_{S}}},{{\overline{\overline{{\mathcal{N}}_{\overline{\overline{{R}_{1}}}}}}}^{U}}^{\overline{{\psi }_{S}}} \right] {e}^{i2\pi \left[ {{\overline{\overline{{\mathcal{N}}_{\overline{\overline{{I}_{1}}}}}}}^{L}}^{\overline{\overline{{\psi }_{S}}}},{{\overline{\overline{{\mathcal{N}}_{\overline{\overline{{I}_{1}}}}}}}^{U}}^{\overline{\overline{{\psi }_{S}}}} \right] } \end{array} \right) \end{eqnarray*}

(9) \begin{eqnarray*}{\overline{\overline{{\mathfrak{C}}_{{C}_{1}}}}}^{\overline{\overline{{\psi }_{S}}}}= \left( \begin{array}{@{}c@{}} \displaystyle \left[ {{\overline{\overline{{\mathcal{M}}_{\overline{\overline{{R}_{1}}}}}}}^{L}}^{\overline{\overline{{\psi }_{S}}}},{{\overline{\overline{{\mathcal{M}}_{\overline{\overline{{R}_{1}}}}}}}^{U}}^{\overline{\overline{{\psi }_{S}}}} \right] {e}^{i2\pi \left[ {{\overline{\overline{{\mathcal{M}}_{\overline{\overline{{I}_{1}}}}}}}^{L}}^{\overline{\overline{{\psi }_{S}}}},{{\overline{\overline{{\mathcal{M}}_{\overline{\overline{{I}_{1}}}}}}}^{U}}^{\overline{\overline{{\psi }_{S}}}} \right] },\\ \displaystyle \left[ \left( 1-{ \left( 1-{\overline{\overline{{\mathcal{N}}_{\overline{\overline{{R}_{1}}}}}}}^{L} \right) }^{\overline{\overline{{\psi }_{S}}}} \right) , \left( 1-{ \left( 1-{\overline{\overline{{\mathcal{N}}_{\overline{\overline{{R}_{1}}}}}}}^{U} \right) }^{\overline{\overline{{\psi }_{S}}}} \right) \right] {e}^{i2\pi \left[ \left( 1-{ \left( 1-{\overline{\overline{{\mathcal{N}}_{\overline{\overline{{I}_{1}}}}}}}^{L} \right) }^{\overline{\overline{{\psi }_{S}}}} \right) , \left( 1-{ \left( 1-{\overline{\overline{{\mathcal{N}}_{\overline{\overline{{I}_{1}}}}}}}^{U} \right) }^{\overline{\overline{{\psi }_{S}}}} \right) \right] } \end{array} \right) \end{eqnarray*}

**Definition 3:**(Garg & Rani, 2019b) By taking any two CIVIFNs

$\overline{\overline{{\mathfrak{C}}_{{C}_{j}}}}= \left( {\scriptsize \begin{array}{@{}c@{}} \displaystyle \left[ {\overline{\overline{{\mathcal{M}}_{\overline{\overline{{R}_{j}}}}}}}^{L} \left( \widetilde {{x}_{E}} \right) ,{\overline{\overline{{\mathcal{M}}_{\overline{\overline{{R}_{j}}}}}}}^{U} \left( \widetilde {{x}_{E}} \right) \right] {e}^{i2\pi \left( \left[ {\overline{\overline{{\mathcal{M}}_{\overline{\overline{{I}_{j}}}}}}}^{L} \left( \widetilde {{x}_{E}} \right) ,{\overline{\overline{{\mathcal{M}}_{\overline{\overline{{I}_{j}}}}}}}^{U} \left( \widetilde {{x}_{E}} \right) \right] \right) },\\ \displaystyle \left[ {\overline{\overline{{\mathcal{N}}_{\overline{\overline{{R}_{j}}}}}}}^{L} \left( \widetilde {{x}_{E}} \right) ,{\overline{\overline{{\mathcal{N}}_{\overline{\overline{{R}_{j}}}}}}}^{U} \left( \widetilde {{x}_{E}} \right) \right] {e}^{i2\pi \left( \left[ {\overline{\overline{{\mathcal{N}}_{\overline{\overline{{I}_{j}}}}}}}^{L} \left( \widetilde {{x}_{E}} \right) ,{\overline{\overline{{\mathcal{N}}_{\overline{\overline{{I}_{j}}}}}}}^{U} \left( \widetilde {{x}_{E}} \right) \right] \right) } \end{array}} \right) $, then the score value (SV) and accuracy value (AV) are determined by the following formulas:

(10) \begin{eqnarray*}\overline{\overline{{\mathcal{S}}_{SV}}} \left( \overline{\overline{{\mathfrak{C}}_{{C}_{1}}}} \right) = \frac{1}{4} \left( {\overline{\overline{{\mathcal{M}}_{\overline{\overline{{R}_{1}}}}}}}^{L}+{\overline{\overline{{\mathcal{M}}_{\overline{\overline{{I}_{1}}}}}}}^{L}-{\overline{\overline{{\mathcal{N}}_{\overline{\overline{{R}_{1}}}}}}}^{L}-{\overline{\overline{{\mathcal{N}}_{\overline{\overline{{I}_{1}}}}}}}^{L}+{\overline{\overline{{\mathcal{M}}_{\overline{\overline{{R}_{1}}}}}}}^{U}+{\overline{\overline{{\mathcal{M}}_{\overline{\overline{{I}_{1}}}}}}}^{U}-{\overline{\overline{{\mathcal{N}}_{\overline{\overline{{R}_{1}}}}}}}^{U}-{\overline{\overline{{\mathcal{N}}_{\overline{\overline{{I}_{1}}}}}}}^{U} \right) ,\end{eqnarray*}

(11) \begin{eqnarray*}\overline{\overline{{\mathcal{H}}_{AV}}} \left( \overline{\overline{{\mathfrak{C}}_{{C}_{1}}}} \right) = \frac{1}{4} \left( {\overline{\overline{{\mathcal{M}}_{\overline{\overline{{R}_{1}}}}}}}^{L}+{\overline{\overline{{\mathcal{M}}_{\overline{\overline{{I}_{1}}}}}}}^{L}+{\overline{\overline{{\mathcal{N}}_{\overline{\overline{{R}_{1}}}}}}}^{L}+{\overline{\overline{{\mathcal{N}}_{\overline{\overline{{I}_{1}}}}}}}^{L}+{\overline{\overline{{\mathcal{M}}_{\overline{\overline{{R}_{1}}}}}}}^{U}+{\overline{\overline{{\mathcal{M}}_{\overline{\overline{{I}_{1}}}}}}}^{U}+{\overline{\overline{{\mathcal{N}}_{\overline{\overline{{R}_{1}}}}}}}^{U}+{\overline{\overline{{\mathcal{N}}_{\overline{\overline{{I}_{1}}}}}}}^{U} \right) \end{eqnarray*}

**Definition 4:**(Garg & Rani, 2019a) By taking any two CIVIFNs $\overline{\overline{{\mathfrak{C}}_{{C}_{j}}}}= \left( {\scriptsize \begin{array}{@{}c@{}} \displaystyle \left[ {\overline{\overline{{\mathcal{M}}_{\overline{\overline{{R}_{j}}}}}}}^{L} \left( \widetilde {{x}_{E}} \right) ,{\overline{\overline{{\mathcal{M}}_{\overline{\overline{{R}_{j}}}}}}}^{U} \left( \widetilde {{x}_{E}} \right) \right] {e}^{i2\pi \left( \left[ {\overline{\overline{{\mathcal{M}}_{\overline{\overline{{I}_{j}}}}}}}^{L} \left( \widetilde {{x}_{E}} \right) ,{\overline{\overline{{\mathcal{M}}_{\overline{\overline{{I}_{j}}}}}}}^{U} \left( \widetilde {{x}_{E}} \right) \right] \right) },\\ \displaystyle \left[ {\overline{\overline{{\mathcal{N}}_{\overline{\overline{{R}_{j}}}}}}}^{L} \left( \widetilde {{x}_{E}} \right) ,{\overline{\overline{{\mathcal{N}}_{\overline{\overline{{R}_{j}}}}}}}^{U} \left( \widetilde {{x}_{E}} \right) \right] {e}^{i2\pi \left( \left[ {\overline{\overline{{\mathcal{N}}_{\overline{\overline{{I}_{j}}}}}}}^{L} \left( \widetilde {{x}_{E}} \right) ,{\overline{\overline{{\mathcal{N}}_{\overline{\overline{{I}_{j}}}}}}}^{U} \left( \widetilde {{x}_{E}} \right) \right] \right) } \end{array}} \right) $, *j* = 1, 2, then

(1) If $\overline{\overline{{\mathcal{S}}_{SV}}} \left( \overline{\overline{{\mathfrak{C}}_{{C}_{1}}}} \right) \gt \overline{\overline{{\mathcal{S}}_{SV}}} \left( \overline{\overline{{\mathfrak{C}}_{{C}_{2}}}} \right) ,~$ then $\overline{\overline{{\mathfrak{C}}_{{C}_{1}}}}\gt \overline{\overline{{\mathfrak{C}}_{{C}_{2}}}}$;
(2) If $\overline{\overline{{\mathcal{S}}_{SV}}} \left( \overline{\overline{{\mathfrak{C}}_{{C}_{1}}}} \right) \lt \overline{\overline{{\mathcal{S}}_{SV}}} \left( \overline{\overline{{\mathfrak{C}}_{{C}_{2}}}} \right) ,~~$ then $\overline{\overline{{\mathfrak{C}}_{{C}_{1}}}}\lt \overline{\overline{{\mathfrak{C}}_{{C}_{2}}}}$;
(3) If $\overline{\overline{{\mathcal{S}}_{SV}}} \left( \overline{\overline{{\mathfrak{C}}_{{C}_{1}}}} \right) =\overline{\overline{{\mathcal{S}}_{SV}}} \left( \overline{\overline{{\mathfrak{C}}_{{C}_{2}}}} \right) ,~~$ then
(i) If $\overline{\overline{{\mathcal{H}}_{AV}}} \left( \overline{\overline{{\mathfrak{C}}_{{C}_{1}}}} \right) \gt \overline{\overline{{\mathcal{H}}_{AV}}} \left( \overline{\overline{{\mathfrak{C}}_{{C}_{2}}}} \right) $, then $\overline{\overline{{\mathfrak{C}}_{{C}_{1}}}}\gt \overline{\overline{{\mathfrak{C}}_{{C}_{2}}}}$;
(ii) If $\overline{\overline{{\mathcal{H}}_{AV}}} \left( \overline{\overline{{\mathfrak{C}}_{{C}_{1}}}} \right) \lt \overline{\overline{{\mathcal{H}}_{AV}}} \left( \overline{\overline{{\mathfrak{C}}_{{C}_{2}}}} \right) $, then $\overline{\overline{{\mathfrak{C}}_{{C}_{1}}}}\lt \overline{\overline{{\mathfrak{C}}_{{C}_{2}}}}$;
(iii) If $\overline{\overline{{\mathcal{H}}_{AV}}} \left( \overline{\overline{{\mathfrak{C}}_{{C}_{1}}}} \right) =\overline{\overline{{\mathcal{H}}_{AV}}} \left( \overline{\overline{{\mathfrak{C}}_{{C}_{2}}}} \right) $, then $\overline{\overline{{\mathfrak{C}}_{{C}_{1}}}}=\overline{\overline{{\mathfrak{C}}_{{C}_{2}}}}$*.*

**Definition 5:**(Klement & Mesiar, 1997) Suppose $\overline{\overline{{\mathbb{T}}_{TN}}}: \left[ 0,1 \right] \times \left[ 0,1 \right] \rightarrow \left[ 0,1 \right] $ states a TN, then

(1) $\overline{\overline{{\mathbb{T}}_{TN}}} \left( \widetilde {{x}_{E}},{\widetilde {{x}_{E}}}^{{}^{{^{\prime}}}} \right) =\overline{\overline{{\mathbb{T}}_{TN}}} \left( {\widetilde {{x}_{E}}}^{{}^{{^{\prime}}}},\widetilde {{x}_{E}} \right) ,\widetilde {{x}_{E}},{\widetilde {{x}_{E}}}^{{}^{{^{\prime}}}}\in \left[ 0,1 \right] $;
(2) $\overline{\overline{{\mathbb{T}}_{TN}}} \left( \widetilde {{x}_{E}},{\widetilde {{x}_{E}}}^{{}^{{^{\prime}}}} \right) \leq \overline{\overline{{\mathbb{T}}_{TN}}} \left( \widetilde {{x}_{E}},{\widetilde {{x}_{E}}}^{{}^{{^{\prime}}{^{\prime}}}} \right) $, if ${\widetilde {{x}_{E}}}^{{}^{{^{\prime}}}}\leq {\widetilde {{x}_{E}}}^{{}^{{^{\prime}}{^{\prime}}}}$;
(3) $\overline{\overline{{\mathbb{T}}_{TN}}} \left( \widetilde {{x}_{E}},\overline{\overline{{\mathbb{T}}_{TN}}} \left( {\widetilde {{x}_{E}}}^{{}^{{^{\prime}}}},{\widetilde {{x}_{E}}}^{{}^{{^{\prime}}{^{\prime}}}} \right) \right) =\overline{\overline{{\mathbb{T}}_{TN}}} \left( \overline{\overline{{\mathbb{T}}_{TN}}} \left( \widetilde {{x}_{E}},{\widetilde {{x}_{E}}}^{{}^{{^{\prime}}}} \right) ,{\widetilde {{x}_{E}}}^{{}^{{^{\prime}}{^{\prime}}}} \right) $;
(4) $\overline{\overline{{\mathbb{T}}_{TN}}} \left( \widetilde {{x}_{E}},1 \right) =\widetilde {{x}_{E}}$.

**Definition 6:**(Klement & Mesiar, 1997) Suppose $\overline{\overline{{\mathbb{S}}_{TN}}}: \left[ 0,1 \right] \times \left[ 0,1 \right] \rightarrow \left[ 0,1 \right] $ states a TCN, then

(1) $\overline{\overline{{\mathbb{S}}_{TN}}} \left( \widetilde {{x}_{E}},{\widetilde {{x}_{E}}}^{{}^{{^{\prime}}}} \right) =\overline{\overline{{\mathbb{S}}_{TN}}} \left( {\widetilde {{x}_{E}}}^{{}^{{^{\prime}}}},\widetilde {{x}_{E}} \right) ,\widetilde {{x}_{E}},{\widetilde {{x}_{E}}}^{{}^{{^{\prime}}}}\in \left[ 0,1 \right] $;
(2) $\overline{\overline{{\mathbb{S}}_{TN}}} \left( \widetilde {{x}_{E}},{\widetilde {{x}_{E}}}^{{}^{{^{\prime}}}} \right) \leq \overline{\overline{{\mathbb{S}}_{TN}}} \left( \widetilde {{x}_{E}},{\widetilde {{x}_{E}}}^{{}^{{^{\prime}}{^{\prime}}}} \right) $, if ${\widetilde {{x}_{E}}}^{{}^{{^{\prime}}}}\leq {\widetilde {{x}_{E}}}^{{}^{{^{\prime}}{^{\prime}}}}$;
(3) $\overline{\overline{{\mathbb{S}}_{TN}}} \left( \widetilde {{x}_{E}},\overline{\overline{{\mathbb{S}}_{TN}}} \left( {\widetilde {{x}_{E}}}^{{}^{{^{\prime}}}},{\widetilde {{x}_{E}}}^{{}^{{^{\prime}}{^{\prime}}}} \right) \right) =\overline{\overline{{\mathbb{S}}_{TN}}} \left( \overline{\overline{{\mathbb{S}}_{TN}}} \left( \widetilde {{x}_{E}},{\widetilde {{x}_{E}}}^{{}^{{^{\prime}}}} \right) ,{\widetilde {{x}_{E}}}^{{}^{{^{\prime}}{^{\prime}}}} \right) $;
(4) $\overline{\overline{{\mathbb{S}}_{TN}}} \left( \widetilde {{x}_{E}},0 \right) =\widetilde {{x}_{E}}$.

**Definition 7:**(Aczél & Alsina, 1982) Suppose ${ \left( {\overline{\overline{{\mathbb{T}}_{TN}}}}_{A}^{\psi } \right) }_{\psi \in \left[ 0,\infty \right] }$ states the Aczel-Alsina TN, its expression is listed as follows:

(12) \begin{eqnarray*}{\overline{\overline{{\mathbb{T}}_{TN}}}}_{A}^{\psi } \left( \widetilde {{x}_{E}},{\widetilde {{x}_{E}}}^{{}^{{^{\prime}}}} \right) = \left\{ \begin{array}{@{}cc@{}} \displaystyle \overline{\overline{{\mathbb{T}}_{TN}}} \left( \widetilde {{x}_{E}},{\widetilde {{x}_{E}}}^{{}^{{^{\prime}}}} \right) &\displaystyle if~\psi =0\\ \displaystyle min \left( \widetilde {{x}_{E}},{\widetilde {{x}_{E}}}^{{}^{{^{\prime}}}} \right) &\displaystyle if~\psi =\infty \\ \displaystyle {e}^{-{ \left( { \left( -log\widetilde {{x}_{E}} \right) }^{\psi }+{ \left( -log{\widetilde {{x}_{E}}}^{{}^{{^{\prime}}}} \right) }^{\psi } \right) }^{ \frac{1}{\psi } }}&\displaystyle otherwise \end{array} \right. \end{eqnarray*}

**Definition 8:**(Aczél & Alsina, 1982) Suppose ${ \left( {\overline{\overline{{\mathbb{S}}_{TN}}}}_{A}^{\psi } \right) }_{\psi \in \left[ 0,\infty \right] }$ states the Aczel-Alsina TCN, the detailed expression is shown as follows:

(13) \begin{eqnarray*}{\overline{\overline{{\mathbb{S}}_{TN}}}}_{A}^{\psi } \left( \widetilde {{x}_{E}},{\widetilde {{x}_{E}}}^{{}^{{^{\prime}}}} \right) = \left\{ \begin{array}{@{}cc@{}} \displaystyle \overline{\overline{{\mathbb{S}}_{TN}}} \left( \widetilde {{x}_{E}},{\widetilde {{x}_{E}}}^{{}^{{^{\prime}}}} \right) &\displaystyle if~\psi =0\\ \displaystyle max \left( \widetilde {{x}_{E}},{\widetilde {{x}_{E}}}^{{}^{{^{\prime}}}} \right) &\displaystyle if~\psi =\infty \\ \displaystyle 1-{e}^{-{ \left( { \left( -\text{log} \left( 1-\widetilde {{x}_{E}} \right) ~ \right) }^{\psi }+{ \left( -\text{log} \left( 1-{\widetilde {{x}_{E}}}^{{}^{{^{\prime}}}} \right) ~ \right) }^{\psi } \right) }^{ \frac{1}{\psi } }}&\displaystyle otherwise \end{array} \right. \end{eqnarray*}

## Aczel-Alsina Operational laws for CIVIFSs

This section mainly introduces the Aczel-Alsina operational laws for CIVIFS that keeps the benefits of the IV information and explores these elementary properties.

**Definition 9:** For CIVIFNs $\overline{\overline{{\mathfrak{C}}_{{C}_{j}}}}= \left( {\scriptsize \begin{array}{@{}c@{}} \displaystyle \left[ {\overline{\overline{{\mathcal{M}}_{\overline{\overline{\overline{{R}_{j}}}}}}}}^{L} \left( \widetilde {{x}_{E}} \right) ,{\overline{\overline{{\mathcal{M}}_{\overline{\overline{{R}_{j}}}}}}}^{U} \left( \widetilde {{x}_{E}} \right) \right] {e}^{i2\pi \left( \left[ {\overline{\overline{{\mathcal{M}}_{\overline{\overline{{I}_{j}}}}}}}^{L} \left( \widetilde {{x}_{E}} \right) ,{\overline{\overline{{\mathcal{M}}_{\overline{\overline{{I}_{j}}}}}}}^{U} \left( \widetilde {{x}_{E}} \right) \right] \right) },\\ \displaystyle \left[ {\overline{\overline{{\mathcal{N}}_{\overline{\overline{{R}_{j}}}}}}}^{L} \left( \widetilde {{x}_{E}} \right) ,{\overline{\overline{{\mathcal{N}}_{\overline{\overline{{R}_{j}}}}}}}^{U} \left( \widetilde {{x}_{E}} \right) \right] {e}^{i2\pi \left( \left[ {\overline{\overline{{\mathcal{N}}_{\overline{\overline{{I}_{j}}}}}}}^{L} \left( \widetilde {{x}_{E}} \right) ,{\overline{\overline{{\mathcal{N}}_{\overline{\overline{{I}_{j}}}}}}}^{U} \left( \widetilde {{x}_{E}} \right) \right] \right) } \end{array}} \right) ,j=1,2$, then

(14) \begin{eqnarray*}\overline{\overline{{\mathfrak{C}}_{{C}_{1}}}}\oplus \overline{\overline{{\mathfrak{C}}_{{C}_{2}}}}= \left( \begin{array}{@{}c@{}} \displaystyle \left[ {\overline{\overline{{\mathbb{S}}_{TN}}}}_{A}^{\psi } \left( {\overline{\overline{{\mathcal{M}}_{\overline{{\overline{R}}_{1}}}}}}^{L},{\overline{\overline{{\mathcal{M}}_{\overline{{\overline{R}}_{2}}}}}}^{L} \right) ,{\overline{\overline{{\mathbb{S}}_{TN}}}}_{A}^{\psi } \left( {\overline{\overline{{\mathcal{M}}_{\overline{{\overline{R}}_{1}}}}}}^{U},{\overline{\overline{{\mathcal{M}}_{\overline{{\overline{R}}_{2}}}}}}^{U} \right) \right] {e}^{i2\pi \left[ {\overline{\overline{{\mathbb{S}}_{TN}}}}_{A}^{\psi } \left( {\overline{\overline{{\mathcal{M}}_{\overline{{\overline{I}}_{1}}}}}}^{L},{\overline{\overline{{\mathcal{M}}_{\overline{{\overline{I}}_{2}}}}}}^{L} \right) ,{\overline{\overline{{\mathbb{S}}_{TN}}}}_{A}^{\psi } \left( {\overline{\overline{{\mathcal{M}}_{\overline{{\overline{I}}_{1}}}}}}^{U},{\overline{\overline{{\mathcal{M}}_{\overline{{\overline{I}}_{2}}}}}}^{U} \right) \right] },\\ \displaystyle \left[ {\overline{\overline{{\mathbb{T}}_{TN}}}}_{A}^{\psi } \left( {\overline{\overline{{\mathcal{N}}_{\overline{{\overline{R}}_{1}}}}}}^{L},{\overline{\overline{{\mathcal{N}}_{\overline{{\overline{R}}_{2}}}}}}^{L} \right) ,{\overline{\overline{{\mathbb{T}}_{TN}}}}_{A}^{\psi } \left( {\overline{\overline{{\mathcal{N}}_{\overline{{\overline{R}}_{1}}}}}}^{U},{\overline{\overline{{\mathcal{N}}_{\overline{{\overline{R}}_{2}}}}}}^{U} \right) \right] {e}^{i2\pi \left[ {\overline{\overline{{\mathbb{T}}_{TN}}}}_{A}^{\psi } \left( {\overline{\overline{{\mathcal{N}}_{\overline{{\overline{I}}_{1}}}}}}^{L},{\overline{\overline{{\mathcal{N}}_{\overline{{\overline{I}}_{2}}}}}}^{L} \right) ,{\overline{\overline{{\mathbb{T}}_{TN}}}}_{A}^{\psi } \left( {\overline{\overline{{\mathcal{N}}_{\overline{{\overline{I}}_{1}}}}}}^{U},{\overline{\overline{{\mathcal{N}}_{\overline{{\overline{I}}_{2}}}}}}^{U} \right) \right] } \end{array} \right) \end{eqnarray*}

(15) \begin{eqnarray*}\overline{\overline{{\mathfrak{C}}_{{C}_{1}}}}\otimes \overline{\overline{{\mathfrak{C}}_{{C}_{2}}}}= \left( \begin{array}{@{}c@{}} \displaystyle \left[ {\overline{\overline{{\mathbb{T}}_{TN}}}}_{A}^{\psi } \left( {\overline{\overline{{\mathcal{M}}_{\overline{{\overline{R}}_{1}}}}}}^{L},{\overline{\overline{{\mathcal{M}}_{\overline{{\overline{R}}_{2}}}}}}^{L} \right) ,{\overline{\overline{{\mathbb{T}}_{TN}}}}_{A}^{\psi } \left( {\overline{\overline{{\mathcal{M}}_{\overline{{\overline{R}}_{1}}}}}}^{U},{\overline{\overline{{\mathcal{M}}_{\overline{{\overline{R}}_{2}}}}}}^{U} \right) \right] {e}^{i2\pi \left[ {\overline{\overline{{\mathbb{T}}_{TN}}}}_{A}^{\psi } \left( {\overline{\overline{{\mathcal{M}}_{\overline{{\overline{I}}_{1}}}}}}^{L},{\overline{\overline{{\mathcal{M}}_{\overline{{\overline{I}}_{2}}}}}}^{L} \right) ,{\overline{\overline{{\mathbb{T}}_{TN}}}}_{A}^{\psi } \left( {\overline{\overline{{\mathcal{M}}_{\overline{{\overline{I}}_{1}}}}}}^{U},{\overline{\overline{{\mathcal{M}}_{\overline{{\overline{I}}_{2}}}}}}^{U} \right) \right] },\\ \displaystyle \left[ {\overline{\overline{{\mathbb{S}}_{TN}}}}_{A}^{\psi } \left( {\overline{\overline{{\mathcal{N}}_{\overline{{\overline{R}}_{1}}}}}}^{L},{\overline{\overline{{\mathcal{N}}_{\overline{{\overline{R}}_{2}}}}}}^{L} \right) ,{\overline{\overline{{\mathbb{S}}_{TN}}}}_{A}^{\psi } \left( {\overline{\overline{{\mathcal{N}}_{\overline{{\overline{R}}_{1}}}}}}^{U},{\overline{\overline{{\mathcal{N}}_{\overline{{\overline{R}}_{2}}}}}}^{U} \right) \right] {e}^{i2\pi \left[ {\overline{\overline{{\mathbb{S}}_{TN}}}}_{A}^{\psi } \left( {\overline{\overline{{\mathcal{N}}_{\overline{{\overline{I}}_{1}}}}}}^{L},{\overline{\overline{{\mathcal{N}}_{\overline{{\overline{I}}_{2}}}}}}^{L} \right) ,{\overline{\overline{{\mathbb{S}}_{TN}}}}_{A}^{\psi } \left( {\overline{\overline{{\mathcal{N}}_{\overline{{\overline{I}}_{1}}}}}}^{U},{\overline{\overline{{\mathcal{N}}_{\overline{{\overline{I}}_{2}}}}}}^{U} \right) \right] } \end{array} \right) \end{eqnarray*}

**Definition 10:** For any two CIVIFNs $\overline{\overline{{\mathfrak{C}}_{{C}_{j}}}}= \left( {\scriptsize \begin{array}{@{}c@{}} \displaystyle \left[ {\overline{\overline{{\mathcal{M}}_{\overline{\overline{{R}_{j}}}}}}}^{L} \left( \widetilde {{x}_{E}} \right) ,{\overline{\overline{{\mathcal{M}}_{\overline{\overline{{R}_{j}}}}}}}^{U} \left( \widetilde {{x}_{E}} \right) \right] {e}^{i2\pi \left( \left[ {\overline{\overline{{\mathcal{M}}_{\overline{\overline{{I}_{j}}}}}}}^{L} \left( \widetilde {{x}_{E}} \right) ,{\overline{\overline{{\mathcal{M}}_{\overline{\overline{{I}_{j}}}}}}}^{U} \left( \widetilde {{x}_{E}} \right) \right] \right) },\\ \displaystyle \left[ {\overline{\overline{{\mathcal{N}}_{\overline{\overline{{R}_{j}}}}}}}^{L} \left( \widetilde {{x}_{E}} \right) ,{\overline{\overline{{\mathcal{N}}_{\overline{\overline{{R}_{j}}}}}}}^{U} \left( \widetilde {{x}_{E}} \right) \right] {e}^{i2\pi \left( \left[ {\overline{\overline{{\mathcal{N}}_{\overline{\overline{{I}_{j}}}}}}}^{L} \left( \widetilde {{x}_{E}} \right) ,{\overline{\overline{{\mathcal{N}}_{\overline{\overline{{I}_{j}}}}}}}^{U} \left( \widetilde {{x}_{E}} \right) \right] \right) } \end{array}} \right) ,j=1,2$, then the following mathematical formulas hold:

(16) \begin{eqnarray*}\overline{\overline{{\mathfrak{C}}_{{C}_{1}}}}\oplus \overline{\overline{{\mathfrak{C}}_{{C}_{2}}}}\nonumber\\\displaystyle = \left( \begin{array}{@{}c@{}} \displaystyle \left[ \left( 1-{e}^{-{ \left( { \left( -\text{log} \left( 1-{\overline{\overline{{\mathcal{M}}_{\overline{{\overline{R}}_{1}}}}}}^{L} \right) ~ \right) }^{\psi }+{ \left( -\text{log} \left( 1-{\overline{\overline{{\mathcal{M}}_{\overline{{\overline{R}}_{2}}}}}}^{L} \right) ~ \right) }^{\psi } \right) }^{ \frac{1}{\psi } }} \right) , \left( 1-{e}^{-{ \left( { \left( -\text{log} \left( 1-{\overline{\overline{{\mathcal{M}}_{\overline{{\overline{R}}_{1}}}}}}^{U} \right) ~ \right) }^{\psi }+{ \left( -\text{log} \left( 1-{\overline{\overline{{\mathcal{M}}_{\overline{{\overline{R}}_{2}}}}}}^{U} \right) ~ \right) }^{\psi } \right) }^{ \frac{1}{\psi } }} \right) \right] \\ \displaystyle {e}^{i2\pi \left[ \left( 1-{e}^{-{ \left( { \left( -\text{log} \left( 1-{\overline{\overline{{\mathcal{M}}_{\overline{{\overline{R}}_{1}}}}}}^{L} \right) ~ \right) }^{\psi }+{ \left( -\text{log} \left( 1-{\overline{\overline{{\mathcal{M}}_{\overline{{\overline{R}}_{2}}}}}}^{L} \right) ~ \right) }^{\psi } \right) }^{ \frac{1}{\psi } }} \right) , \left( 1-{e}^{-{ \left( { \left( -\text{log} \left( 1-{\overline{\overline{{\mathcal{M}}_{\overline{{\overline{R}}_{1}}}}}}^{U} \right) ~ \right) }^{\psi }+{ \left( -\text{log} \left( 1-{\overline{\overline{{\mathcal{M}}_{\overline{{\overline{R}}_{2}}}}}}^{U} \right) ~ \right) }^{\psi } \right) }^{ \frac{1}{\psi } }} \right) \right] },\\ \displaystyle \left[ \left( {e}^{-{ \left( { \left( -\text{log} \left( {\overline{\overline{{\mathcal{N}}_{\overline{{\overline{R}}_{1}}}}}}^{L} \right) ~ \right) }^{\psi }+{ \left( -\text{log} \left( {\overline{\overline{{\mathcal{N}}_{\overline{{\overline{R}}_{2}}}}}}^{L} \right) ~ \right) }^{\psi } \right) }^{ \frac{1}{\psi } }} \right) , \left( {e}^{-{ \left( { \left( -\text{log} \left( {\overline{\overline{{\mathcal{N}}_{\overline{{\overline{R}}_{1}}}}}}^{U} \right) ~ \right) }^{\psi }+{ \left( -\text{log} \left( {\overline{\overline{{\mathcal{N}}_{\overline{{\overline{R}}_{2}}}}}}^{U} \right) ~ \right) }^{\psi } \right) }^{ \frac{1}{\psi } }} \right) \right] \\ \displaystyle {e}^{i2\pi \left[ \left( {e}^{-{ \left( { \left( -\text{log} \left( {\overline{\overline{{\mathcal{N}}_{\overline{{\overline{I}}_{1}}}}}}^{L} \right) ~ \right) }^{\psi }+{ \left( -\text{log} \left( {\overline{\overline{{\mathcal{N}}_{\overline{{\overline{I}}_{2}}}}}}^{L} \right) ~ \right) }^{\psi } \right) }^{ \frac{1}{\psi } }} \right) , \left( {e}^{-{ \left( { \left( -\text{log} \left( {\overline{\overline{{\mathcal{N}}_{\overline{{\overline{I}}_{1}}}}}}^{U} \right) ~ \right) }^{\psi }+{ \left( -\text{log} \left( {\overline{\overline{{\mathcal{N}}_{\overline{{\overline{I}}_{2}}}}}}^{U} \right) ~ \right) }^{\psi } \right) }^{ \frac{1}{\psi } }} \right) \right] } \end{array} \right) \end{eqnarray*}

(17) \begin{eqnarray*}\overline{\overline{{\mathfrak{C}}_{{C}_{1}}}}\otimes \overline{\overline{{\mathfrak{C}}_{{C}_{2}}}}\nonumber\\\displaystyle = \left( \begin{array}{@{}c@{}} \displaystyle \left[ \left( {e}^{-{ \left( { \left( -\text{log} \left( {\overline{\overline{{\mathcal{M}}_{\overline{{\overline{R}}_{1}}}}}}^{L} \right) ~ \right) }^{\psi }+{ \left( -\text{log} \left( {\overline{\overline{{\mathcal{M}}_{\overline{{\overline{R}}_{2}}}}}}^{L} \right) ~ \right) }^{\psi } \right) }^{ \frac{1}{\psi } }} \right) , \left( {e}^{-{ \left( { \left( -\text{log} \left( {\overline{\overline{{\mathcal{M}}_{\overline{{\overline{R}}_{1}}}}}}^{U} \right) ~ \right) }^{\psi }+{ \left( -\text{log} \left( {\overline{\overline{{\mathcal{M}}_{\overline{{\overline{R}}_{2}}}}}}^{U} \right) ~ \right) }^{\psi } \right) }^{ \frac{1}{\psi } }} \right) \right] \\ \displaystyle {e}^{i2\pi \left[ \left( {e}^{-{ \left( { \left( -\text{log} \left( {\overline{\overline{{\mathcal{M}}_{\overline{{\overline{I}}_{1}}}}}}^{L} \right) ~ \right) }^{\psi }+{ \left( -\text{log} \left( {\overline{\overline{{\mathcal{M}}_{\overline{{\overline{I}}_{2}}}}}}^{L} \right) ~ \right) }^{\psi } \right) }^{ \frac{1}{\psi } }} \right) , \left( {e}^{-{ \left( { \left( -\text{log} \left( {\overline{\overline{{\mathcal{M}}_{\overline{{\overline{I}}_{1}}}}}}^{U} \right) ~ \right) }^{\psi }+{ \left( -\text{log} \left( {\overline{\overline{{\mathcal{M}}_{\overline{{\overline{I}}_{2}}}}}}^{U} \right) ~ \right) }^{\psi } \right) }^{ \frac{1}{\psi } }} \right) \right] },\\ \displaystyle \left[ \left( 1-{e}^{-{ \left( { \left( -\text{log} \left( 1-{\overline{\overline{{\mathcal{N}}_{\overline{{\overline{R}}_{1}}}}}}^{L} \right) ~ \right) }^{\psi }+{ \left( -\text{log} \left( 1-{\overline{\overline{{\mathcal{N}}_{\overline{{\overline{R}}_{2}}}}}}^{L} \right) ~ \right) }^{\psi } \right) }^{ \frac{1}{\psi } }} \right) , \left( 1-{e}^{-{ \left( { \left( -\text{log} \left( 1-{\overline{\overline{{\mathcal{N}}_{\overline{{\overline{R}}_{1}}}}}}^{U} \right) ~ \right) }^{\psi }+{ \left( -\text{log} \left( 1-{\overline{\overline{{\mathcal{N}}_{\overline{{\overline{R}}_{2}}}}}}^{U} \right) ~ \right) }^{\psi } \right) }^{ \frac{1}{\psi } }} \right) \right] \\ \displaystyle {e}^{i2\pi \left[ \left( 1-{e}^{-{ \left( { \left( -\text{log} \left( 1-{\overline{\overline{{\mathcal{N}}_{\overline{{\overline{R}}_{1}}}}}}^{L} \right) ~ \right) }^{\psi }+{ \left( -\text{log} \left( 1-{\overline{\overline{{\mathcal{N}}_{\overline{{\overline{R}}_{2}}}}}}^{L} \right) ~ \right) }^{\psi } \right) }^{ \frac{1}{\psi } }} \right) , \left( 1-{e}^{-{ \left( { \left( -\text{log} \left( 1-{\overline{\overline{{\mathcal{N}}_{\overline{{\overline{R}}_{1}}}}}}^{U} \right) ~ \right) }^{\psi }+{ \left( -\text{log} \left( 1-{\overline{\overline{{\mathcal{N}}_{\overline{{\overline{R}}_{2}}}}}}^{U} \right) ~ \right) }^{\psi } \right) }^{ \frac{1}{\psi } }} \right) \right] } \end{array} \right) \end{eqnarray*}

(18) \begin{eqnarray*}\overline{\overline{{\theta }_{S}}\overline{\overline{{\mathfrak{C}}_{{C}_{1}}}}}= \left( \begin{array}{@{}c@{}} \displaystyle \left[ \left( 1-{e}^{-{ \left( \overline{\overline{{\theta }_{S}}}{ \left( -\text{log} \left( 1-{\overline{\overline{{\mathcal{M}}_{\overline{{\overline{R}}_{1}}}}}}^{L} \right) ~ \right) }^{\psi } \right) }^{ \frac{1}{\psi } }} \right) , \left( 1-{e}^{-{ \left( \overline{\overline{{\theta }_{S}}}{ \left( -\text{log} \left( 1-{\overline{\overline{{\mathcal{M}}_{\overline{{\overline{R}}_{1}}}}}}^{U} \right) ~ \right) }^{\psi } \right) }^{ \frac{1}{\psi } }} \right) \right] \\ \displaystyle {e}^{i2\pi \left[ \left( 1-{e}^{-{ \left( \overline{\overline{{\theta }_{S}}}{ \left( -\text{log} \left( 1-{\overline{\overline{{\mathcal{M}}_{\overline{{\overline{I}}_{1}}}}}}^{L} \right) ~ \right) }^{\psi } \right) }^{ \frac{1}{\psi } }} \right) , \left( 1-{e}^{-{ \left( \overline{\overline{{\theta }_{S}}}{ \left( -\text{log} \left( 1-{\overline{\overline{{\mathcal{M}}_{\overline{{\overline{I}}_{1}}}}}}^{U} \right) ~ \right) }^{\psi } \right) }^{ \frac{1}{\psi } }} \right) \right] },\\ \displaystyle \left[ \left( {e}^{-{ \left( \overline{\overline{{\theta }_{S}}}{ \left( -\text{log} \left( {\overline{\overline{{\mathcal{N}}_{\overline{{\overline{R}}_{1}}}}}}^{L} \right) ~ \right) }^{\psi } \right) }^{ \frac{1}{\psi } }} \right) , \left( {e}^{-{ \left( \overline{\overline{{\theta }_{S}}}{ \left( -\text{log} \left( {\overline{\overline{{\mathcal{N}}_{\overline{{\overline{R}}_{1}}}}}}^{U} \right) ~ \right) }^{\psi } \right) }^{ \frac{1}{\psi } }} \right) \right] \\ \displaystyle {e}^{i2\pi \left[ \left( {e}^{-{ \left( \overline{\overline{{\theta }_{S}}}{ \left( -\text{log} \left( {\overline{\overline{{\mathcal{N}}_{\overline{{\overline{I}}_{1}}}}}}^{L} \right) ~ \right) }^{\psi } \right) }^{ \frac{1}{\psi } }} \right) , \left( {e}^{-{ \left( \overline{\overline{{\theta }_{S}}}{ \left( -\text{log} \left( {\overline{\overline{{\mathcal{N}}_{\overline{{\overline{I}}_{1}}}}}}^{U} \right) ~ \right) }^{\psi } \right) }^{ \frac{1}{\psi } }} \right) \right] } \end{array} \right) \end{eqnarray*}

(19) \begin{eqnarray*}{\overline{\overline{{\mathfrak{C}}_{{C}_{1}}}}}^{\overline{\overline{{\theta }_{S}}}}= \left( \begin{array}{@{}c@{}} \displaystyle \left[ \left( {e}^{-{ \left( \overline{\overline{{\theta }_{S}}}{ \left( -\text{log} \left( {\overline{\overline{{\mathcal{M}}_{\overline{{\overline{R}}_{1}}}}}}^{L} \right) ~ \right) }^{\psi } \right) }^{ \frac{1}{\psi } }} \right) , \left( {e}^{-{ \left( \overline{\overline{{\theta }_{S}}}{ \left( -\text{log} \left( {\overline{\overline{{\mathcal{M}}_{\overline{{\overline{R}}_{1}}}}}}^{U} \right) ~ \right) }^{\psi } \right) }^{ \frac{1}{\psi } }} \right) \right] \\ \displaystyle {e}^{i2\pi \left[ \left( {e}^{-{ \left( \overline{\overline{{\theta }_{S}}}{ \left( -\text{log} \left( {\overline{\overline{{\mathcal{M}}_{\overline{{\overline{I}}_{1}}}}}}^{L} \right) ~ \right) }^{\psi } \right) }^{ \frac{1}{\psi } }} \right) , \left( {e}^{-{ \left( \overline{\overline{{\theta }_{S}}}{ \left( -\text{log} \left( {\overline{\overline{{\mathcal{M}}_{\overline{{\overline{I}}_{1}}}}}}^{U} \right) ~ \right) }^{\psi } \right) }^{ \frac{1}{\psi } }} \right) \right] },\\ \displaystyle \left[ \left( 1-{e}^{-{ \left( \overline{\overline{{\theta }_{S}}}{ \left( -\text{log} \left( 1-{\overline{\overline{{\mathcal{N}}_{\overline{\overline{{R}_{1}}}}}}}^{L} \right) ~ \right) }^{\psi } \right) }^{ \frac{1}{\psi } }} \right) , \left( 1-{e}^{-{ \left( \overline{\overline{{\theta }_{S}}}{ \left( -\text{log} \left( 1-{\overline{\overline{{\mathcal{N}}_{\overline{\overline{{R}_{1}}}}}}}^{U} \right) ~ \right) }^{\psi } \right) }^{ \frac{1}{\psi } }} \right) \right] \\ \displaystyle {e}^{i2\pi \left[ \left( 1-{e}^{-{ \left( \overline{\overline{{\theta }_{S}}}{ \left( -\text{log} \left( 1-{\overline{\overline{{\mathcal{N}}_{\overline{\overline{{I}_{1}}}}}}}^{L} \right) ~ \right) }^{\psi } \right) }^{ \frac{1}{\psi } }} \right) , \left( 1-{e}^{-{ \left( \overline{\overline{{\theta }_{S}}}{ \left( -\text{log} \left( 1-{\overline{\overline{{\mathcal{N}}_{\overline{\overline{{I}_{1}}}}}}}^{U} \right) ~ \right) }^{\psi } \right) }^{ \frac{1}{\psi } }} \right) \right] } \end{array} \right) \end{eqnarray*}

**Theorem 1:** For any two CIVIFNs $\overline{\overline{{\mathfrak{C}}_{{C}_{j}}}}= \left( {\scriptsize \begin{array}{@{}c@{}} \displaystyle \left[ {\overline{\overline{{\mathcal{M}}_{\overline{\overline{{R}_{j}}}}}}}^{L} \left( \widetilde {{x}_{E}} \right) ,{\overline{\overline{{\mathcal{M}}_{\overline{\overline{{R}_{j}}}}}}}^{U} \left( \widetilde {{x}_{E}} \right) \right] {e}^{i2\pi \left( \left[ {\overline{\overline{{\mathcal{M}}_{\overline{\overline{{I}_{j}}}}}}}^{L} \left( \widetilde {{x}_{E}} \right) ,{\overline{\overline{{\mathcal{M}}_{\overline{\overline{{I}_{j}}}}}}}^{U} \left( \widetilde {{x}_{E}} \right) \right] \right) },\\ \displaystyle \left[ {\overline{\overline{{\mathcal{N}}_{\overline{\overline{{R}_{j}}}}}}}^{L} \left( \widetilde {{x}_{E}} \right) ,{\overline{\overline{{\mathcal{N}}_{\overline{\overline{{R}_{j}}}}}}}^{U} \left( \widetilde {{x}_{E}} \right) \right] {e}^{i2\pi \left( \left[ {\overline{\overline{{\mathcal{N}}_{\overline{\overline{{I}_{j}}}}}}}^{L} \left( \widetilde {{x}_{E}} \right) ,{\overline{\overline{{\mathcal{N}}_{\overline{\overline{{I}_{j}}}}}}}^{U} \left( \widetilde {{x}_{E}} \right) \right] \right) } \end{array}} \right) ,j=1,2$, then we can derive the subsequent mathematic properties:

(1) $\overline{\overline{{\mathfrak{C}}_{{C}_{1}}}}\oplus \overline{\overline{{\mathfrak{C}}_{{C}_{2}}}}=\overline{\overline{{\mathfrak{C}}_{{C}_{2}}}}\oplus \overline{\overline{{\mathfrak{C}}_{{C}_{1}}}}$;
(2) $\overline{\overline{{\mathfrak{C}}_{{C}_{1}}}}\otimes \overline{\overline{{\mathfrak{C}}_{{C}_{2}}}}=\overline{\overline{{\mathfrak{C}}_{{C}_{2}}}}\otimes \overline{\overline{{\mathfrak{C}}_{{C}_{1}}}}$;
(3) $\overline{\overline{{\theta }_{S}}} \left( \overline{\overline{{\mathfrak{C}}_{{C}_{1}}}}\oplus \overline{\overline{{\mathfrak{C}}_{{C}_{2}}}} \right) =\overline{{\overline{\theta }}_{S}\overline{\overline{{\mathfrak{C}}_{{C}_{1}}}}}\oplus \overline{\overline{{\theta }_{S}}}\overline{\overline{{\mathfrak{C}}_{{C}_{2}}}}$;
(4) $ \left( \overline{{\theta }_{{S}_{1}}}+\overline{{\theta }_{{S}_{1}}} \right) \overline{\overline{{\mathfrak{C}}_{{C}_{1}}}}=\overline{{\theta }_{{S}_{1}}}\overline{\overline{{\mathfrak{C}}_{{C}_{1}}}}\oplus \overline{{\theta }_{{S}_{2}}}\overline{\overline{{\mathfrak{C}}_{{C}_{1}}}}$;
(5) ${ \left( \overline{\overline{{\mathfrak{C}}_{{C}_{1}}}}\otimes \overline{\overline{{\mathfrak{C}}_{{C}_{2}}}} \right) }^{\overline{{\theta }_{S}}}={\overline{\overline{{\mathfrak{C}}_{{C}_{1}}}}}^{\overline{{\theta }_{S}}}\otimes {\overline{\overline{{\mathfrak{C}}_{{C}_{2}}}}}^{\overline{{\theta }_{S}}}$;
(6) ${\overline{\overline{{\mathfrak{C}}_{{C}_{1}}}}}^{\overline{\overline{{\theta }_{{S}_{1}}}}}\otimes {\overline{\overline{{\mathfrak{C}}_{{C}_{1}}}}}^{\overline{\overline{{\theta }_{{S}_{1}}}}}={\overline{\overline{{\mathfrak{C}}_{{C}_{1}}}}}^{\overline{\overline{{\theta }_{{S}_{1}}}}+\overline{\overline{{\theta }_{{S}_{2}}}}}$.

**Proof:** Next we present the proofs of properties (1), (3) and (5), as we can similarly complete the proofs of properties (2), (4) and (6).

(1) On the basis of Eqs. (16) to (19), then we calculate $\overline{\overline{{\mathfrak{C}}_{{C}_{1}}}}\oplus \overline{\overline{{\mathfrak{C}}_{{C}_{2}}}}$:
  \begin{eqnarray*}\overline{\overline{{\mathfrak{C}}_{{C}_{1}}}}\oplus \overline{\overline{{\mathfrak{C}}_{{C}_{2}}}}\nonumber\\\displaystyle = \left( \begin{array}{@{}c@{}} \displaystyle \left[ \left( 1-{e}^{-{ \left( { \left( -\text{log} \left( 1-{\overline{\overline{{\mathcal{M}}_{\overline{\overline{{R}_{1}}}}}}}^{L} \right) ~ \right) }^{\psi }+{ \left( -\text{log} \left( 1-{\overline{\overline{{\mathcal{M}}_{\overline{\overline{{R}_{2}}}}}}}^{L} \right) ~ \right) }^{\psi } \right) }^{ \frac{1}{\psi } }} \right) , \left( 1-{e}^{-{ \left( { \left( -\text{log} \left( 1-{\overline{\overline{{\mathcal{M}}_{\overline{\overline{{R}_{1}}}}}}}^{U} \right) ~ \right) }^{\psi }+{ \left( -\text{log} \left( 1-{\overline{\overline{{\mathcal{M}}_{\overline{\overline{{R}_{2}}}}}}}^{U} \right) ~ \right) }^{\psi } \right) }^{ \frac{1}{\psi } }} \right) \right] \\ \displaystyle {e}^{i2\pi \left[ \left( 1-{e}^{-{ \left( { \left( -\text{log} \left( 1-{\overline{\overline{{\mathcal{M}}_{\overline{\overline{{R}_{1}}}}}}}^{L} \right) ~ \right) }^{\psi }+{ \left( -\text{log} \left( 1-{\overline{\overline{{\mathcal{M}}_{\overline{\overline{{R}_{2}}}}}}}^{L} \right) ~ \right) }^{\psi } \right) }^{ \frac{1}{\psi } }} \right) , \left( 1-{e}^{-{ \left( { \left( -\text{log} \left( 1-{\overline{\overline{{\mathcal{M}}_{\overline{\overline{{R}_{1}}}}}}}^{U} \right) ~ \right) }^{\psi }+{ \left( -\text{log} \left( 1-{\overline{\overline{{\mathcal{M}}_{\overline{\overline{{R}_{2}}}}}}}^{U} \right) ~ \right) }^{\psi } \right) }^{ \frac{1}{\psi } }} \right) \right] },\\ \displaystyle \left[ \left( {e}^{-{ \left( { \left( -\text{log} \left( {\overline{\overline{{\mathcal{N}}_{\overline{\overline{{R}_{1}}}}}}}^{L} \right) ~ \right) }^{\psi }+{ \left( -\text{log} \left( {\overline{{\mathcal{N}}_{\overline{{R}_{2}}}}}^{L} \right) ~ \right) }^{\psi } \right) }^{ \frac{1}{\psi } }} \right) , \left( {e}^{-{ \left( { \left( -\text{log} \left( {\overline{\overline{{\mathcal{N}}_{\overline{\overline{{R}_{1}}}}}}}^{U} \right) ~ \right) }^{\psi }+{ \left( -\text{log} \left( {\overline{{\mathcal{N}}_{\overline{{R}_{2}}}}}^{U} \right) ~ \right) }^{\psi } \right) }^{ \frac{1}{\psi } }} \right) \right] \\ \displaystyle {e}^{i2\pi \left[ \left( {e}^{-{ \left( { \left( -\text{log} \left( {\overline{\overline{{\mathcal{N}}_{\overline{\overline{{I}_{1}}}}}}}^{L} \right) ~ \right) }^{\psi }+{ \left( -\text{log} \left( {\overline{\overline{{\mathcal{N}}_{\overline{\overline{{I}_{2}}}}}}}^{L} \right) ~ \right) }^{\psi } \right) }^{ \frac{1}{\psi } }} \right) , \left( {e}^{-{ \left( { \left( -\text{log} \left( {\overline{\overline{{\mathcal{N}}_{\overline{\overline{{I}_{1}}}}}}}^{U} \right) ~ \right) }^{\psi }+{ \left( -\text{log} \left( {\overline{\overline{{\mathcal{N}}_{\overline{\overline{{I}_{2}}}}}}}^{U} \right) ~ \right) }^{\psi } \right) }^{ \frac{1}{\psi } }} \right) \right] } \end{array} \right) \end{eqnarray*}

\begin{eqnarray*}= \left( \begin{array}{@{}c@{}} \displaystyle \left[ \left( 1-{e}^{-{ \left( { \left( -\text{log} \left( 1-{\overline{\overline{{\mathcal{M}}_{\overline{\overline{{R}_{2}}}}}}}^{L} \right) ~ \right) }^{\psi }+{ \left( -\text{log} \left( 1-{\overline{\overline{{\mathcal{M}}_{\overline{\overline{{R}_{1}}}}}}}^{L} \right) ~ \right) }^{\psi } \right) }^{ \frac{1}{\psi } }} \right) , \left( 1-{e}^{-{ \left( { \left( -\text{log} \left( 1-{\overline{\overline{{\mathcal{M}}_{\overline{\overline{{R}_{2}}}}}}}^{U} \right) ~ \right) }^{\psi }+{ \left( -\text{log} \left( 1-{\overline{\overline{{\mathcal{M}}_{\overline{\overline{{R}_{1}}}}}}}^{U} \right) ~ \right) }^{\psi } \right) }^{ \frac{1}{\psi } }} \right) \right] \\ \displaystyle {e}^{i2\pi \left[ \left( 1-{e}^{-{ \left( { \left( -\text{log} \left( 1-{\overline{\overline{{\mathcal{M}}_{\overline{\overline{{R}_{2}}}}}}}^{L} \right) ~ \right) }^{\psi }+{ \left( -\text{log} \left( 1-{\overline{\overline{{\mathcal{M}}_{\overline{\overline{{R}_{1}}}}}}}^{L} \right) ~ \right) }^{\psi } \right) }^{ \frac{1}{\psi } }} \right) , \left( 1-{e}^{-{ \left( { \left( -\text{log} \left( 1-{\overline{\overline{{\mathcal{M}}_{\overline{\overline{{R}_{2}}}}}}}^{U} \right) ~ \right) }^{\psi }+{ \left( -\text{log} \left( 1-{\overline{\overline{{\mathcal{M}}_{\overline{\overline{{R}_{1}}}}}}}^{U} \right) ~ \right) }^{\psi } \right) }^{ \frac{1}{\psi } }} \right) \right] },\\ \displaystyle \left[ \left( {e}^{-{ \left( { \left( -\text{log} \left( {\overline{{\mathcal{N}}_{\overline{{R}_{2}}}}}^{L} \right) ~ \right) }^{\psi }+{ \left( -\text{log} \left( {\overline{\overline{{\mathcal{N}}_{\overline{\overline{{R}_{1}}}}}}}^{L} \right) ~ \right) }^{\psi } \right) }^{ \frac{1}{\psi } }} \right) , \left( {e}^{-{ \left( { \left( -\text{log} \left( {\overline{{\mathcal{N}}_{\overline{{R}_{2}}}}}^{U} \right) ~ \right) }^{\psi }+{ \left( -\text{log} \left( {\overline{\overline{{\mathcal{N}}_{\overline{\overline{{R}_{1}}}}}}}^{U} \right) ~ \right) }^{\psi } \right) }^{ \frac{1}{\psi } }} \right) \right] \\ \displaystyle {e}^{i2\pi \left[ \left( {e}^{-{ \left( { \left( -\text{log} \left( {\overline{\overline{{\mathcal{N}}_{\overline{\overline{{I}_{2}}}}}}}^{L} \right) ~ \right) }^{\psi }+{ \left( -\text{log} \left( {\overline{\overline{{\mathcal{N}}_{\overline{\overline{{I}_{1}}}}}}}^{L} \right) ~ \right) }^{\psi } \right) }^{ \frac{1}{\psi } }} \right) , \left( {e}^{-{ \left( { \left( -\text{log} \left( {\overline{\overline{{\mathcal{N}}_{\overline{\overline{{I}_{2}}}}}}}^{U} \right) ~ \right) }^{\psi }+{ \left( -\text{log} \left( {\overline{\overline{{\mathcal{N}}_{\overline{\overline{{I}_{1}}}}}}}^{U} \right) ~ \right) }^{\psi } \right) }^{ \frac{1}{\psi } }} \right) \right] } \end{array} \right) \end{eqnarray*}

\begin{eqnarray*}=\overline{\overline{{\mathfrak{C}}_{{C}_{2}}}}\oplus \overline{\overline{{\mathfrak{C}}_{{C}_{1}}}}. \end{eqnarray*}

Hence, we investigated $\overline{\overline{{\mathfrak{C}}_{{C}_{1}}}}\oplus \overline{\overline{{\mathfrak{C}}_{{C}_{2}}}}=\overline{\overline{{\mathfrak{C}}_{{C}_{2}}}}\oplus \overline{\overline{{\mathfrak{C}}_{{C}_{1}}}}$, the property (1) is proved.

(2) Suppose $\overline{\overline{{\theta }_{S}}} \left( \overline{\overline{{\mathfrak{C}}_{{C}_{1}}}}\oplus \overline{\overline{{\mathfrak{C}}_{{C}_{2}}}} \right) $, then
  \begin{eqnarray*}\overline{\overline{{\theta }_{S}}} \left( \overline{\overline{{\mathfrak{C}}_{{C}_{1}}}}\oplus \overline{\overline{{\mathfrak{C}}_{{C}_{2}}}} \right) \nonumber\\\displaystyle =\overline{\overline{{\theta }_{S}}} \left( \begin{array}{@{}c@{}} \displaystyle \left[ \left( 1-{e}^{-{ \left( { \left( -\text{log} \left( 1-{\overline{\overline{{\mathcal{M}}_{\overline{\overline{{R}_{1}}}}}}}^{L} \right) ~ \right) }^{\psi }+{ \left( -\text{log} \left( 1-{\overline{\overline{{\mathcal{M}}_{\overline{\overline{{R}_{2}}}}}}}^{L} \right) ~ \right) }^{\psi } \right) }^{ \frac{1}{\psi } }} \right) , \left( 1-{e}^{-{ \left( { \left( -\text{log} \left( 1-{\overline{\overline{{\mathcal{M}}_{\overline{\overline{{R}_{1}}}}}}}^{U} \right) ~ \right) }^{\psi }+{ \left( -\text{log} \left( 1-{\overline{\overline{{\mathcal{M}}_{\overline{\overline{{R}_{2}}}}}}}^{U} \right) ~ \right) }^{\psi } \right) }^{ \frac{1}{\psi } }} \right) \right] \\ \displaystyle {e}^{i2\pi \left[ \left( 1-{e}^{-{ \left( { \left( -\text{log} \left( 1-{\overline{\overline{{\mathcal{M}}_{\overline{\overline{{R}_{1}}}}}}}^{L} \right) ~ \right) }^{\psi }+{ \left( -\text{log} \left( 1-{\overline{\overline{{\mathcal{M}}_{\overline{\overline{{R}_{2}}}}}}}^{L} \right) ~ \right) }^{\psi } \right) }^{ \frac{1}{\psi } }} \right) , \left( 1-{e}^{-{ \left( { \left( -\text{log} \left( 1-{\overline{\overline{{\mathcal{M}}_{\overline{\overline{{R}_{1}}}}}}}^{U} \right) ~ \right) }^{\psi }+{ \left( -\text{log} \left( 1-{\overline{\overline{{\mathcal{M}}_{\overline{\overline{{R}_{2}}}}}}}^{U} \right) ~ \right) }^{\psi } \right) }^{ \frac{1}{\psi } }} \right) \right] },\\ \displaystyle \left[ \left( {e}^{-{ \left( { \left( -\text{log} \left( {\overline{\overline{{\mathcal{N}}_{\overline{\overline{{R}_{1}}}}}}}^{L} \right) ~ \right) }^{\psi }+{ \left( -\text{log} \left( {\overline{\overline{{\mathcal{N}}_{\overline{\overline{{R}_{2}}}}}}}^{L} \right) ~ \right) }^{\psi } \right) }^{ \frac{1}{\psi } }} \right) , \left( {e}^{-{ \left( { \left( -\text{log} \left( {\overline{\overline{{\mathcal{N}}_{\overline{\overline{{R}_{1}}}}}}}^{U} \right) ~ \right) }^{\psi }+{ \left( -\text{log} \left( {\overline{\overline{{\mathcal{N}}_{\overline{\overline{{R}_{2}}}}}}}^{U} \right) ~ \right) }^{\psi } \right) }^{ \frac{1}{\psi } }} \right) \right] \\ \displaystyle {e}^{i2\pi \left[ \left( {e}^{-{ \left( { \left( -\text{log} \left( {\overline{\overline{{\mathcal{N}}_{\overline{\overline{{I}_{1}}}}}}}^{L} \right) ~ \right) }^{\psi }+{ \left( -\text{log} \left( {\overline{\overline{{\mathcal{N}}_{\overline{\overline{{I}_{2}}}}}}}^{L} \right) ~ \right) }^{\psi } \right) }^{ \frac{1}{\psi } }} \right) , \left( {e}^{-{ \left( { \left( -\text{log} \left( {\overline{\overline{{\mathcal{N}}_{\overline{\overline{{I}_{1}}}}}}}^{U} \right) ~ \right) }^{\psi }+{ \left( -\text{log} \left( {\overline{\overline{{\mathcal{N}}_{\overline{\overline{{I}_{2}}}}}}}^{U} \right) ~ \right) }^{\psi } \right) }^{ \frac{1}{\psi } }} \right) \right] } \end{array} \right) \end{eqnarray*}

\begin{eqnarray*}= \left( \begin{array}{@{}c@{}} \displaystyle \left[ \left( 1-{e}^{-{ \left( \overline{\overline{{\theta }_{S}}}{ \left( -\text{log} \left( 1-{\overline{\overline{{\mathcal{M}}_{\overline{\overline{{R}_{1}}}}}}}^{L} \right) ~ \right) }^{\psi }+{ \left( -\text{log} \left( 1-{\overline{\overline{{\mathcal{M}}_{\overline{\overline{{R}_{2}}}}}}}^{L} \right) ~ \right) }^{\psi } \right) }^{ \frac{1}{\psi } }} \right) , \left( 1-{e}^{-{ \left( \overline{\overline{{\theta }_{S}}}{ \left( -\text{log} \left( 1-{\overline{\overline{{\mathcal{M}}_{\overline{\overline{{R}_{1}}}}}}}^{U} \right) ~ \right) }^{\psi }+{ \left( -\text{log} \left( 1-{\overline{\overline{{\mathcal{M}}_{\overline{\overline{{R}_{2}}}}}}}^{U} \right) ~ \right) }^{\psi } \right) }^{ \frac{1}{\psi } }} \right) \right] \\ \displaystyle {e}^{i2\pi \left[ \left( 1-{e}^{-{ \left( \overline{\overline{{\theta }_{S}}}{ \left( -\text{log} \left( 1-{\overline{\overline{{\mathcal{M}}_{\overline{\overline{{R}_{1}}}}}}}^{L} \right) ~ \right) }^{\psi }+{ \left( -\text{log} \left( 1-{\overline{\overline{{\mathcal{M}}_{\overline{\overline{{R}_{2}}}}}}}^{L} \right) ~ \right) }^{\psi } \right) }^{ \frac{1}{\psi } }} \right) , \left( 1-{e}^{-{ \left( \overline{\overline{{\theta }_{S}}}{ \left( -\text{log} \left( 1-{\overline{\overline{{\mathcal{M}}_{\overline{\overline{{R}_{1}}}}}}}^{U} \right) ~ \right) }^{\psi }+{ \left( -\text{log} \left( 1-{\overline{\overline{{\mathcal{M}}_{\overline{\overline{{R}_{2}}}}}}}^{U} \right) ~ \right) }^{\psi } \right) }^{ \frac{1}{\psi } }} \right) \right] },\\ \displaystyle \left[ \left( {e}^{-{ \left( \overline{\overline{{\theta }_{S}}}{ \left( -\text{log} \left( {\overline{\overline{{\mathcal{N}}_{\overline{\overline{{R}_{1}}}}}}}^{L} \right) ~ \right) }^{\psi }+{ \left( -\text{log} \left( {\overline{{\mathcal{N}}_{\overline{{R}_{2}}}}}^{L} \right) ~ \right) }^{\psi } \right) }^{ \frac{1}{\psi } }} \right) , \left( {e}^{-{ \left( \overline{\overline{{\theta }_{S}}}{ \left( -\text{log} \left( {\overline{\overline{{\mathcal{N}}_{\overline{\overline{{R}_{1}}}}}}}^{U} \right) ~ \right) }^{\psi }+{ \left( -\text{log} \left( {\overline{{\mathcal{N}}_{\overline{{R}_{2}}}}}^{U} \right) ~ \right) }^{\psi } \right) }^{ \frac{1}{\psi } }} \right) \right] \\ \displaystyle {e}^{i2\pi \left[ \left( {e}^{-{ \left( \overline{\overline{{\theta }_{S}}}{ \left( -\text{log} \left( {\overline{\overline{{\mathcal{N}}_{\overline{\overline{{I}_{1}}}}}}}^{L} \right) ~ \right) }^{\psi }+{ \left( -\text{log} \left( {\overline{\overline{{\mathcal{N}}_{\overline{\overline{{I}_{2}}}}}}}^{L} \right) ~ \right) }^{\psi } \right) }^{ \frac{1}{\psi } }} \right) , \left( {e}^{-{ \left( \overline{\overline{{\theta }_{S}}}{ \left( -\text{log} \left( {\overline{\overline{{\mathcal{N}}_{\overline{\overline{{I}_{1}}}}}}}^{U} \right) ~ \right) }^{\psi }+{ \left( -\text{log} \left( {\overline{\overline{{\mathcal{N}}_{\overline{\overline{{I}_{2}}}}}}}^{U} \right) ~ \right) }^{\psi } \right) }^{ \frac{1}{\psi } }} \right) \right] } \end{array} \right) \end{eqnarray*}

\begin{eqnarray*}= \left( \begin{array}{@{}c@{}} \displaystyle \left[ \left( 1-{e}^{-{ \left( \overline{\overline{{\theta }_{S}}}{ \left( -\text{log} \left( 1-{\overline{\overline{{\mathcal{M}}_{\overline{\overline{{R}_{1}}}}}}}^{L} \right) ~ \right) }^{\psi } \right) }^{ \frac{1}{\psi } }} \right) , \left( 1-{e}^{-{ \left( \overline{\overline{{\theta }_{S}}}{ \left( -\text{log} \left( 1-{\overline{\overline{{\mathcal{M}}_{\overline{\overline{{R}_{1}}}}}}}^{U} \right) ~ \right) }^{\psi } \right) }^{ \frac{1}{\psi } }} \right) \right] \\ \displaystyle {e}^{i2\pi \left[ \left( 1-{e}^{-{ \left( \overline{\overline{{\theta }_{S}}}{ \left( -\text{log} \left( 1-{\overline{\overline{{\mathcal{M}}_{\overline{\overline{{I}_{1}}}}}}}^{L} \right) ~ \right) }^{\psi } \right) }^{ \frac{1}{\psi } }} \right) , \left( 1-{e}^{-{ \left( \overline{\overline{{\theta }_{S}}}{ \left( -\text{log} \left( 1-{\overline{\overline{{\mathcal{M}}_{\overline{\overline{{I}_{1}}}}}}}^{U} \right) ~ \right) }^{\psi } \right) }^{ \frac{1}{\psi } }} \right) \right] },\\ \displaystyle \left[ \left( {e}^{-{ \left( \overline{\overline{{\theta }_{S}}}{ \left( -\text{log} \left( {\overline{\overline{{\mathcal{N}}_{\overline{\overline{{R}_{1}}}}}}}^{L} \right) ~ \right) }^{\psi } \right) }^{ \frac{1}{\psi } }} \right) , \left( {e}^{-{ \left( \overline{\overline{{\theta }_{S}}}{ \left( -\text{log} \left( {\overline{\overline{{\mathcal{N}}_{\overline{\overline{{R}_{1}}}}}}}^{U} \right) ~ \right) }^{\psi } \right) }^{ \frac{1}{\psi } }} \right) \right] \\ \displaystyle {e}^{i2\pi \left[ \left( {e}^{-{ \left( \overline{\overline{{\theta }_{S}}}{ \left( -\text{log} \left( {\overline{\overline{{\mathcal{N}}_{\overline{\overline{{I}_{1}}}}}}}^{L} \right) ~ \right) }^{\psi } \right) }^{ \frac{1}{\psi } }} \right) , \left( {e}^{-{ \left( \overline{\overline{{\theta }_{S}}}{ \left( -\text{log} \left( {\overline{\overline{{\mathcal{N}}_{\overline{\overline{{I}_{1}}}}}}}^{U} \right) ~ \right) }^{\psi } \right) }^{ \frac{1}{\psi } }} \right) \right] } \end{array} \right) \end{eqnarray*}

\begin{eqnarray*}\oplus \left( \begin{array}{@{}c@{}} \displaystyle \left[ \left( 1-{e}^{-{ \left( \overline{\overline{{\theta }_{S}}}{ \left( -\text{log} \left( 1-{\overline{\overline{{\mathcal{M}}_{\overline{\overline{{R}_{2}}}}}}}^{L} \right) ~ \right) }^{\psi } \right) }^{ \frac{1}{\psi } }} \right) , \left( 1-{e}^{-{ \left( \overline{\overline{{\theta }_{S}}}{ \left( -\text{log} \left( 1-{\overline{\overline{{\mathcal{M}}_{\overline{\overline{{R}_{2}}}}}}}^{U} \right) ~ \right) }^{\psi } \right) }^{ \frac{1}{\psi } }} \right) \right] \\ \displaystyle {e}^{i2\pi \left[ \left( 1-{e}^{-{ \left( \overline{\overline{{\theta }_{S}}}{ \left( -\text{log} \left( 1-{\overline{\overline{{\mathcal{M}}_{\overline{\overline{{I}_{2}}}}}}}^{L} \right) ~ \right) }^{\psi } \right) }^{ \frac{1}{\psi } }} \right) , \left( 1-{e}^{-{ \left( \overline{\overline{{\theta }_{S}}}{ \left( -\text{log} \left( 1-{\overline{\overline{{\mathcal{M}}_{\overline{\overline{{I}_{2}}}}}}}^{U} \right) ~ \right) }^{\psi } \right) }^{ \frac{1}{\psi } }} \right) \right] },\\ \displaystyle \left[ \left( {e}^{-{ \left( \overline{\overline{{\theta }_{S}}}{ \left( -\text{log} \left( {\overline{\overline{{\mathcal{N}}_{\overline{\overline{{R}_{2}}}}}}}^{L} \right) ~ \right) }^{\psi } \right) }^{ \frac{1}{\psi } }} \right) , \left( {e}^{-{ \left( \overline{\overline{{\theta }_{S}}}{ \left( -\text{log} \left( {\overline{\overline{{\mathcal{N}}_{\overline{\overline{{R}_{2}}}}}}}^{U} \right) ~ \right) }^{\psi } \right) }^{ \frac{1}{\psi } }} \right) \right] \\ \displaystyle {e}^{i2\pi \left[ \left( {e}^{-{ \left( \overline{\overline{{\theta }_{S}}}{ \left( -\text{log} \left( {\overline{\overline{{\mathcal{N}}_{\overline{\overline{{I}_{2}}}}}}}^{L} \right) ~ \right) }^{\psi } \right) }^{ \frac{1}{\psi } }} \right) , \left( {e}^{-{ \left( \overline{\overline{{\theta }_{S}}}{ \left( -\text{log} \left( {\overline{\overline{{\mathcal{N}}_{\overline{\overline{{I}_{2}}}}}}}^{U} \right) ~ \right) }^{\psi } \right) }^{ \frac{1}{\psi } }} \right) \right] } \end{array} \right) \end{eqnarray*}

\begin{eqnarray*}=\overline{\overline{{\theta }_{S}}}\overline{\overline{{\mathfrak{C}}_{{C}_{1}}}}\oplus \overline{\overline{{\theta }_{S}}}\overline{\overline{{\mathfrak{C}}_{{C}_{2}}}}. \end{eqnarray*}

Hence, $\overline{{\theta }_{S}} \left( \overline{\overline{{\mathfrak{C}}_{{C}_{1}}}}\oplus \overline{\overline{{\mathfrak{C}}_{{C}_{2}}}} \right) =\overline{\overline{{\theta }_{S}}}\overline{\overline{{\mathfrak{C}}_{{C}_{1}}}}\oplus \overline{\overline{{\theta }_{S}}}\overline{\overline{{\mathfrak{C}}_{{C}_{2}}}}$, the property (3) is proved.

(3) Suppose ${ \left( \overline{\overline{{\mathfrak{C}}_{{C}_{1}}}}\otimes \overline{\overline{{\mathfrak{C}}_{{C}_{2}}}} \right) }^{\overline{\overline{{\theta }_{S}}}}$, then
  \begin{eqnarray*}{ \left( \overline{\overline{{\mathfrak{C}}_{{C}_{1}}}}\otimes \overline{\overline{{\mathfrak{C}}_{{C}_{2}}}} \right) }^{\overline{\overline{{\theta }_{S}}}}\nonumber\\\displaystyle ={ \left( \begin{array}{@{}c@{}} \displaystyle \left[ \left( {e}^{-{ \left( { \left( -\text{log} \left( {\overline{\overline{{\mathcal{M}}_{\overline{\overline{{R}_{1}}}}}}}^{L} \right) ~ \right) }^{\psi }+{ \left( -\text{log} \left( {\overline{\overline{{\mathcal{M}}_{\overline{\overline{{R}_{2}}}}}}}^{L} \right) ~ \right) }^{\psi } \right) }^{ \frac{1}{\psi } }} \right) , \left( {e}^{-{ \left( { \left( -\text{log} \left( {\overline{\overline{{\mathcal{M}}_{\overline{\overline{{R}_{1}}}}}}}^{U} \right) ~ \right) }^{\psi }+{ \left( -\text{log} \left( {\overline{\overline{{\mathcal{M}}_{\overline{\overline{{R}_{2}}}}}}}^{U} \right) ~ \right) }^{\psi } \right) }^{ \frac{1}{\psi } }} \right) \right] \\ \displaystyle {e}^{i2\pi \left[ \left( {e}^{-{ \left( { \left( -\text{log} \left( {\overline{\overline{{\mathcal{M}}_{\overline{\overline{{I}_{1}}}}}}}^{L} \right) ~ \right) }^{\psi }+{ \left( -\text{log} \left( {\overline{\overline{{\mathcal{M}}_{\overline{\overline{{I}_{2}}}}}}}^{L} \right) ~ \right) }^{\psi } \right) }^{ \frac{1}{\psi } }} \right) , \left( {e}^{-{ \left( { \left( -\text{log} \left( {\overline{\overline{{\mathcal{M}}_{\overline{\overline{{I}_{1}}}}}}}^{U} \right) ~ \right) }^{\psi }+{ \left( -\text{log} \left( {\overline{\overline{{\mathcal{M}}_{\overline{\overline{{I}_{2}}}}}}}^{U} \right) ~ \right) }^{\psi } \right) }^{ \frac{1}{\psi } }} \right) \right] },\\ \displaystyle \left[ \left( 1-{e}^{-{ \left( { \left( -\text{log} \left( 1-{\overline{\overline{{\mathcal{N}}_{\overline{\overline{{R}_{1}}}}}}}^{L} \right) ~ \right) }^{\psi }+{ \left( -\text{log} \left( 1-{\overline{\overline{{\mathcal{N}}_{\overline{\overline{{R}_{2}}}}}}}^{L} \right) ~ \right) }^{\psi } \right) }^{ \frac{1}{\psi } }} \right) , \left( 1-{e}^{-{ \left( { \left( -\text{log} \left( 1-{\overline{\overline{{\mathcal{N}}_{\overline{\overline{{R}_{1}}}}}}}^{U} \right) ~ \right) }^{\psi }+{ \left( -\text{log} \left( 1-{\overline{\overline{{\mathcal{N}}_{\overline{\overline{{R}_{2}}}}}}}^{U} \right) ~ \right) }^{\psi } \right) }^{ \frac{1}{\psi } }} \right) \right] \\ \displaystyle {e}^{i2\pi \left[ \left( 1-{e}^{-{ \left( { \left( -\text{log} \left( 1-{\overline{\overline{{\mathcal{N}}_{\overline{\overline{{R}_{1}}}}}}}^{L} \right) ~ \right) }^{\psi }+{ \left( -\text{log} \left( 1-{\overline{\overline{{\mathcal{N}}_{\overline{\overline{{R}_{2}}}}}}}^{L} \right) ~ \right) }^{\psi } \right) }^{ \frac{1}{\psi } }} \right) , \left( 1-{e}^{-{ \left( { \left( -\text{log} \left( 1-{\overline{\overline{{\mathcal{N}}_{\overline{\overline{{R}_{1}}}}}}}^{U} \right) ~ \right) }^{\psi }+{ \left( -\text{log} \left( 1-{\overline{\overline{{\mathcal{N}}_{\overline{\overline{{R}_{2}}}}}}}^{U} \right) ~ \right) }^{\psi } \right) }^{ \frac{1}{\psi } }} \right) \right] } \end{array} \right) }^{\overline{\overline{{\theta }_{S}}}} \end{eqnarray*}
  \begin{eqnarray*}= \left( \begin{array}{@{}c@{}} \displaystyle \left[ \left( {e}^{-{ \left( \overline{\overline{{\theta }_{S}}}{ \left( -\text{log} \left( {\overline{\overline{{\mathcal{M}}_{\overline{\overline{{R}_{1}}}}}}}^{L} \right) ~ \right) }^{\psi } \right) }^{ \frac{1}{\psi } }} \right) , \left( {e}^{-{ \left( \overline{\overline{{\theta }_{S}}}{ \left( -\text{log} \left( {\overline{\overline{{\mathcal{M}}_{\overline{\overline{{R}_{1}}}}}}}^{U} \right) ~ \right) }^{\psi } \right) }^{ \frac{1}{\psi } }} \right) \right] \\ \displaystyle {e}^{i2\pi \left[ \left( {e}^{-{ \left( \overline{\overline{{\theta }_{S}}}{ \left( -\text{log} \left( {\overline{\overline{{\mathcal{M}}_{\overline{\overline{{I}_{1}}}}}}}^{L} \right) ~ \right) }^{\psi } \right) }^{ \frac{1}{\psi } }} \right) , \left( {e}^{-{ \left( \overline{\overline{{\theta }_{S}}}{ \left( -\text{log} \left( {\overline{\overline{{\mathcal{M}}_{\overline{\overline{{I}_{1}}}}}}}^{U} \right) ~ \right) }^{\psi } \right) }^{ \frac{1}{\psi } }} \right) \right] },\\ \displaystyle \left[ \left( 1-{e}^{-{ \left( \overline{\overline{{\theta }_{S}}}{ \left( -\text{log} \left( 1-{\overline{\overline{{\mathcal{N}}_{\overline{\overline{{R}_{1}}}}}}}^{L} \right) ~ \right) }^{\psi } \right) }^{ \frac{1}{\psi } }} \right) , \left( 1-{e}^{-{ \left( \overline{\overline{{\theta }_{S}}}{ \left( -\text{log} \left( 1-{\overline{\overline{{\mathcal{N}}_{\overline{\overline{{R}_{1}}}}}}}^{U} \right) ~ \right) }^{\psi } \right) }^{ \frac{1}{\psi } }} \right) \right] \\ \displaystyle {e}^{i2\pi \left[ \left( 1-{e}^{-{ \left( \overline{\overline{{\theta }_{S}}}{ \left( -\text{log} \left( 1-{\overline{\overline{{\mathcal{N}}_{\overline{\overline{{I}_{1}}}}}}}^{L} \right) ~ \right) }^{\psi } \right) }^{ \frac{1}{\psi } }} \right) , \left( 1-{e}^{-{ \left( \overline{\overline{{\theta }_{S}}}{ \left( -\text{log} \left( 1-{\overline{\overline{{\mathcal{N}}_{\overline{\overline{{I}_{1}}}}}}}^{U} \right) ~ \right) }^{\psi } \right) }^{ \frac{1}{\psi } }} \right) \right] } \end{array} \right) \end{eqnarray*}
  \begin{eqnarray*}\otimes \left( \begin{array}{@{}c@{}} \displaystyle \left[ \left( {e}^{-{ \left( \overline{\overline{{\theta }_{S}}}{ \left( -\text{log} \left( {\overline{\overline{{\mathcal{M}}_{\overline{\overline{{R}_{2}}}}}}}^{L} \right) ~ \right) }^{\psi } \right) }^{ \frac{1}{\psi } }} \right) , \left( {e}^{-{ \left( \overline{\overline{{\theta }_{S}}}{ \left( -\text{log} \left( {\overline{\overline{{\mathcal{M}}_{\overline{\overline{{R}_{2}}}}}}}^{U~} \right) ~ \right) }^{\psi } \right) }^{ \frac{1}{\psi } }} \right) \right] \\ \displaystyle {e}^{i2\pi \left[ \left( {e}^{-{ \left( \overline{\overline{{\theta }_{S}}}{ \left( -\text{log} \left( {\overline{\overline{{\mathcal{M}}_{\overline{\overline{{I}_{2}}}}}}}^{L} \right) ~ \right) }^{\psi } \right) }^{ \frac{1}{\psi } }} \right) , \left( {e}^{-{ \left( \overline{\overline{{\theta }_{S}}}{ \left( -\text{log} \left( {\overline{\overline{{\mathcal{M}}_{\overline{\overline{{I}_{2}}}}}}}^{U} \right) ~ \right) }^{\psi } \right) }^{ \frac{1}{\psi } }} \right) \right] },\\ \displaystyle \left[ \left( 1-{e}^{-{ \left( \overline{\overline{{\theta }_{S}}}{ \left( -\text{log} \left( 1-{\overline{\overline{{\mathcal{N}}_{\overline{\overline{{R}_{2}}}}}}}^{L} \right) ~ \right) }^{\psi } \right) }^{ \frac{1}{\psi } }} \right) , \left( 1-{e}^{-{ \left( \overline{\overline{{\theta }_{S}}}{ \left( -\text{log} \left( 1-{\overline{\overline{{\mathcal{N}}_{\overline{\overline{{R}_{2}}}}}}}^{U} \right) ~ \right) }^{\psi } \right) }^{ \frac{1}{\psi } }} \right) \right] \\ \displaystyle {e}^{i2\pi \left[ \left( 1-{e}^{-{ \left( \overline{\overline{{\theta }_{S}}}{ \left( -\text{log} \left( 1-{\overline{\overline{{\mathcal{N}}_{\overline{\overline{{I}_{2}}}}}}}^{L} \right) ~ \right) }^{\psi } \right) }^{ \frac{1}{\psi } }} \right) , \left( 1-{e}^{-{ \left( \overline{\overline{{\theta }_{S}}}{ \left( -\text{log} \left( 1-{\overline{\overline{{\mathcal{N}}_{\overline{\overline{{I}_{2}}}}}}}^{U} \right) ~ \right) }^{\psi } \right) }^{ \frac{1}{\psi } }} \right) \right] } \end{array} \right) \end{eqnarray*}
  \begin{eqnarray*}={\overline{\overline{{\mathfrak{C}}_{{C}_{1}}}}}^{\overline{\overline{{\theta }_{S}}}}\otimes {\overline{\overline{{\mathfrak{C}}_{{C}_{2}}}}}^{\overline{\overline{{\theta }_{S}}}} \end{eqnarray*}

Hence, ${ \left( \overline{\overline{{\mathfrak{C}}_{{C}_{1}}}}\otimes \overline{\overline{{\mathfrak{C}}_{{C}_{2}}}} \right) }^{\overline{\overline{{\theta }_{S}}}}={\overline{\overline{{\mathfrak{C}}_{{C}_{1}}}}}^{\overline{\overline{{\theta }_{S}}}}\otimes {\overline{\overline{{\mathfrak{C}}_{{C}_{2}}}}}^{\overline{\overline{{\theta }_{S}}}}$, and the property (5) is proved.

## Aczel-Alsina Aggregation Operators for CIVIFS

This section proposes a group of aggregation operators by utilizing the Aczel-Alsina operational laws for CIVIFSs such that CIVIFAAWA, CIVIFAAOWA, CIVIFAAHA, CIVIFAAWG, CIVIFAAOWG, and CIVIFAAHG operators, and illustrates their well-known properties.

**Definition 11:** For CIVIFNs $\overline{\overline{{\mathfrak{C}}_{{C}_{j}}}}= \left( {\scriptsize \begin{array}{@{}c@{}} \displaystyle \left[ {\overline{\overline{{\mathcal{M}}_{\overline{\overline{{R}_{j}}}}}}}^{L} \left( \widetilde {{x}_{E}} \right) ,{\overline{\overline{{\mathcal{M}}_{\overline{\overline{{R}_{j}}}}}}}^{U} \left( \widetilde {{x}_{E}} \right) \right] {e}^{i2\pi \left( \left[ {\overline{\overline{{\mathcal{M}}_{\overline{\overline{{I}_{j}}}}}}}^{L} \left( \widetilde {{x}_{E}} \right) ,{\overline{\overline{{\mathcal{M}}_{\overline{\overline{{I}_{j}}}}}}}^{U} \left( \widetilde {{x}_{E}} \right) \right] \right) },\\ \displaystyle \left[ {\overline{\overline{{\mathcal{N}}_{\overline{\overline{{R}_{j}}}}}}}^{L} \left( \widetilde {{x}_{E}} \right) ,{\overline{\overline{{\mathcal{N}}_{\overline{\overline{{R}_{j}}}}}}}^{U} \left( \widetilde {{x}_{E}} \right) \right] {e}^{i2\pi \left( \left[ {\overline{\overline{{\mathcal{N}}_{\overline{\overline{{I}_{j}}}}}}}^{L} \left( \widetilde {{x}_{E}} \right) ,{\overline{\overline{{\mathcal{N}}_{\overline{\overline{{I}_{j}}}}}}}^{U} \left( \widetilde {{x}_{E}} \right) \right] \right) } \end{array}} \right) ,j=1,\ldots ,n$, then the CIVIFAAWA operator is interpreted as:

(20) \begin{eqnarray*}CIVIFAAWA \left( \overline{\overline{{\mathfrak{C}}_{{C}_{1}}}},\overline{\overline{{\mathfrak{C}}_{{C}_{2}}}},\ldots ,\overline{\overline{{\mathfrak{C}}_{{C}_{n}}}} \right) =\overline{\overline{{\mathfrak{W}}_{1}}}\overline{\overline{{\mathfrak{C}}_{{C}_{1}}}}\oplus \overline{\overline{{\mathfrak{W}}_{2}}}\overline{\overline{{\mathfrak{C}}_{{C}_{2}}}}\oplus \cdots \oplus \overline{\overline{{\mathfrak{W}}_{n}}}\overline{\overline{{\mathfrak{C}}_{{C}_{n}}}}={\mathop{\oplus \nolimits }\nolimits }_{j=1}^{n} \left( \overline{\overline{{\mathfrak{W}}_{j}}}\overline{\overline{{\mathfrak{C}}_{{C}_{j}}}} \right) \end{eqnarray*}

where $\overline{\overline{\mathfrak{W}}}={ \left( {\overline{\overline{\mathfrak{W}}}}_{1},{\overline{\overline{\mathfrak{W}}}}_{2},\ldots ,{\overline{\overline{\mathfrak{W}}}}_{n} \right) }^{T}$means the weight of *C*_*j*_, with a rule ${\mathop{\sum }\nolimits }_{j=1}^{n}{\overline{\overline{\mathfrak{W}}}}_{j}=1$.

**Theorem 2:** For CIVIFNs $\overline{\overline{{\mathfrak{C}}_{{C}_{j}}}}= \left( {\scriptsize \begin{array}{@{}c@{}} \displaystyle \left[ {\overline{\overline{{\mathcal{M}}_{\overline{\overline{{R}_{j}}}}}}}^{L} \left( \widetilde {{x}_{E}} \right) ,{\overline{\overline{{\mathcal{M}}_{\overline{\overline{{R}_{j}}}}}}}^{U} \left( \widetilde {{x}_{E}} \right) \right] {e}^{i2\pi \left( \left[ {\overline{\overline{{\mathcal{M}}_{\overline{\overline{{I}_{j}}}}}}}^{L} \left( \widetilde {{x}_{E}} \right) ,{\overline{\overline{{\mathcal{M}}_{\overline{\overline{{I}_{j}}}}}}}^{U} \left( \widetilde {{x}_{E}} \right) \right] \right) },\\ \displaystyle \left[ {\overline{\overline{{\mathcal{N}}_{\overline{\overline{{R}_{j}}}}}}}^{L} \left( \widetilde {{x}_{E}} \right) ,{\overline{\overline{{\mathcal{N}}_{\overline{\overline{{R}_{j}}}}}}}^{U} \left( \widetilde {{x}_{E}} \right) \right] {e}^{i2\pi \left( \left[ {\overline{\overline{{\mathcal{N}}_{\overline{\overline{{I}_{j}}}}}}}^{L} \left( \widetilde {{x}_{E}} \right) ,{\overline{\overline{{\mathcal{N}}_{\overline{\overline{{I}_{j}}}}}}}^{U} \left( \widetilde {{x}_{E}} \right) \right] \right) } \end{array}} \right) ,j=1,\ldots ,n$, then by using Eq. (20), we elaborate

\begin{eqnarray*}CIVIFAAWA \left( \overline{\overline{{\mathfrak{C}}_{{C}_{1}}}},\overline{\overline{{\mathfrak{C}}_{{C}_{2}}}},\ldots ,\overline{\overline{{\mathfrak{C}}_{{C}_{n}}}} \right) \end{eqnarray*}

(21) \begin{eqnarray*}= \left( \begin{array}{@{}c@{}} \displaystyle \left[ \left( 1-{e}^{-{ \left( \sum _{j=1}^{n}\overline{\overline{{\mathfrak{W}}_{j}}}{ \left( -\text{log} \left( 1-{\overline{\overline{{\mathcal{M}}_{\overline{\overline{{R}_{j}}}}}}}^{L} \right) ~ \right) }^{\psi } \right) }^{ \frac{1}{\psi } }} \right) , \left( 1-{e}^{-{ \left( \sum _{j=1}^{n}\overline{\overline{{\mathfrak{W}}_{j}}}{ \left( -\text{log} \left( 1-{\overline{\overline{{\mathcal{M}}_{\overline{\overline{{R}_{j}}}}}}}^{U} \right) ~ \right) }^{\psi } \right) }^{ \frac{1}{\psi } }} \right) \right] \\ \displaystyle {e}^{i2\pi \left[ \left( 1-{e}^{-{ \left( \sum _{j=1}^{n}\overline{\overline{{\mathfrak{W}}_{j}}}{ \left( -\text{log} \left( 1-{\overline{\overline{{\mathcal{M}}_{\overline{\overline{{I}_{j}}}}}}}^{L} \right) ~ \right) }^{\psi } \right) }^{ \frac{1}{\psi } }} \right) , \left( 1-{e}^{-{ \left( \sum _{j=1}^{n}\overline{\overline{{\mathfrak{W}}_{j}}}{ \left( -\text{log} \left( 1-{\overline{\overline{{\mathcal{M}}_{\overline{\overline{{I}_{j}}}}}}}^{U} \right) ~ \right) }^{\psi } \right) }^{ \frac{1}{\psi } }} \right) \right] },\\ \displaystyle \left[ \left( {e}^{-{ \left( \sum _{j=1}^{n}\overline{\overline{{\mathfrak{W}}_{j}}}{ \left( -\text{log} \left( {\overline{\overline{{\mathcal{N}}_{\overline{\overline{{R}_{j}}}}}}}^{L} \right) ~ \right) }^{\psi } \right) }^{ \frac{1}{\psi } }} \right) , \left( {e}^{-{ \left( \sum _{j=1}^{n}\overline{\overline{{\mathfrak{W}}_{j}}}{ \left( -\text{log} \left( {\overline{\overline{{\mathcal{N}}_{\overline{\overline{{R}_{j}}}}}}}^{U} \right) ~ \right) }^{\psi } \right) }^{ \frac{1}{\psi } }} \right) \right] \\ \displaystyle {e}^{i2\pi \left[ \left( {e}^{-{ \left( \sum _{j=1}^{n}\overline{\overline{{\mathfrak{W}}_{j}}}{ \left( -\text{log} \left( {\overline{\overline{{\mathcal{N}}_{\overline{\overline{{I}_{j}}}}}}}^{L} \right) ~ \right) }^{\psi } \right) }^{ \frac{1}{\psi } }} \right) , \left( {e}^{-{ \left( \sum _{j=1}^{n}\overline{\overline{{\mathfrak{W}}_{j}}}{ \left( -\text{log} \left( {\overline{\overline{{\mathcal{N}}_{\overline{\overline{{I}_{j}}}}}}}^{U} \right) ~ \right) }^{\psi } \right) }^{ \frac{1}{\psi } }} \right) \right] } \end{array} \right) \end{eqnarray*}

Furthermore, we derive the theory of idempotency, boundedness, and monotonicity for the information in Eq. (21).

**Property 1:** For CIVIFNs $\overline{\overline{{\mathfrak{C}}_{{C}_{j}}}}= \left( {\scriptsize \begin{array}{@{}c@{}} \displaystyle \left[ {\overline{\overline{{\mathcal{M}}_{\overline{\overline{{R}_{j}}}}}}}^{L} \left( \widetilde {{x}_{E}} \right) ,{\overline{\overline{{\mathcal{M}}_{\overline{\overline{{R}_{j}}}}}}}^{U} \left( \widetilde {{x}_{E}} \right) \right] {e}^{i2\pi \left( \left[ {\overline{\overline{{\mathcal{M}}_{\overline{\overline{{I}_{j}}}}}}}^{L} \left( \widetilde {{x}_{E}} \right) ,{\overline{\overline{{\mathcal{M}}_{\overline{\overline{{I}_{j}}}}}}}^{U} \left( \widetilde {{x}_{E}} \right) \right] \right) },\\ \displaystyle \left[ {\overline{\overline{{\mathcal{N}}_{\overline{\overline{{R}_{j}}}}}}}^{L} \left( \widetilde {{x}_{E}} \right) ,{\overline{\overline{{\mathcal{N}}_{\overline{\overline{{R}_{j}}}}}}}^{U} \left( \widetilde {{x}_{E}} \right) \right] {e}^{i2\pi \left( \left[ {\overline{\overline{{\mathcal{N}}_{\overline{\overline{{I}_{j}}}}}}}^{L} \left( \widetilde {{x}_{E}} \right) ,{\overline{\overline{{\mathcal{N}}_{\overline{\overline{{I}_{j}}}}}}}^{U} \left( \widetilde {{x}_{E}} \right) \right] \right) } \end{array}} \right) ,j=1,2,\ldots ,n$, if $\overline{\overline{{\mathfrak{C}}_{{C}_{j}}}}=\overline{\overline{\mathfrak{C}}}$, then the detailed expression is shown as:

(22) \begin{eqnarray*}CIVIFAAWA \left( \overline{\overline{{\mathfrak{C}}_{{C}_{1}}}},\overline{\overline{{\mathfrak{C}}_{{C}_{2}}}},\ldots ,\overline{\overline{{\mathfrak{C}}_{{C}_{n}}}} \right) =\overline{\overline{\mathfrak{C}}}\end{eqnarray*}

**Proof:** Suppose $\overline{\overline{{\mathfrak{C}}_{{C}_{j}}}}=\overline{\overline{\mathfrak{C}}}= \left( \left[ {\overline{\overline{{\mathcal{M}}_{\overline{\overline{R}}}}}}^{L},{\overline{\overline{{\mathcal{M}}_{\overline{\overline{R}}}}}}^{U} \right] {e}^{i2\pi \left( \left[ {\overline{\overline{{\mathcal{M}}_{\overline{\overline{I}}}}}}^{L},{\overline{\overline{{\mathcal{M}}_{\overline{\overline{I}}}}}}^{U} \right] \right) }, \left[ {\overline{\overline{{\mathcal{N}}_{\overline{\overline{R}}}}}}^{L},{\overline{\overline{{\mathcal{N}}_{\overline{\overline{R}}}}}}^{U} \right] {e}^{i2\pi \left( \left[ {\overline{\overline{{\mathcal{N}}_{\overline{\overline{I}}}}}}^{L},{\overline{\overline{{\mathcal{N}}_{\overline{\overline{I}}}}}}^{U} \right] \right) } \right) $, then

\begin{eqnarray*}CIVIFAAWA \left( \overline{\overline{{\mathfrak{C}}_{{C}_{1}}}},\overline{\overline{{\mathfrak{C}}_{{C}_{2}}}},\ldots ,\overline{\overline{{\mathfrak{C}}_{{C}_{n}}}} \right) = \left( \begin{array}{@{}c@{}} \displaystyle \left[ \left( 1-{e}^{-{ \left( \sum _{j=1}^{n}\overline{\overline{{\mathfrak{W}}_{j}}}{ \left( -\text{log} \left( 1-{\overline{\overline{{\mathcal{M}}_{\overline{\overline{{R}_{j}}}}}}}^{L} \right) ~ \right) }^{\psi } \right) }^{ \frac{1}{\psi } }} \right) , \left( 1-{e}^{-{ \left( \sum _{j=1}^{n}\overline{\overline{{\mathfrak{W}}_{j}}}{ \left( -\text{log} \left( 1-{\overline{\overline{{\mathcal{M}}_{\overline{\overline{{R}_{j}}}}}}}^{U} \right) ~ \right) }^{\psi } \right) }^{ \frac{1}{\psi } }} \right) \right] \\ \displaystyle {e}^{i2\pi \left[ \left( 1-{e}^{-{ \left( \sum _{j=1}^{n}\overline{\overline{{\mathfrak{W}}_{j}}}{ \left( -\text{log} \left( 1-{\overline{\overline{{\mathcal{M}}_{\overline{\overline{{I}_{j}}}}}}}^{L} \right) ~ \right) }^{\psi } \right) }^{ \frac{1}{\psi } }} \right) , \left( 1-{e}^{-{ \left( \sum _{j=1}^{n}\overline{\overline{{\mathfrak{W}}_{j}}}{ \left( -\text{log} \left( 1-{\overline{\overline{{\mathcal{M}}_{\overline{\overline{{I}_{j}}}}}}}^{U} \right) ~ \right) }^{\psi } \right) }^{ \frac{1}{\psi } }} \right) \right] },\\ \displaystyle \left[ \left( {e}^{-{ \left( \sum _{j=1}^{n}\overline{\overline{{\mathfrak{W}}_{j}}}{ \left( -\text{log} \left( {\overline{\overline{{\mathcal{N}}_{\overline{\overline{{R}_{j}}}}}}}^{L} \right) ~ \right) }^{\psi } \right) }^{ \frac{1}{\psi } }} \right) , \left( {e}^{-{ \left( \sum _{j=1}^{n}\overline{\overline{{\mathfrak{W}}_{j}}}{ \left( -\text{log} \left( {\overline{\overline{{\mathcal{N}}_{\overline{\overline{{R}_{j}}}}}}}^{U} \right) ~ \right) }^{\psi } \right) }^{ \frac{1}{\psi } }} \right) \right] \\ \displaystyle {e}^{i2\pi \left[ \left( {e}^{-{ \left( \sum _{j=1}^{n}\overline{\overline{{\mathfrak{W}}_{j}}}{ \left( -\text{log} \left( {\overline{\overline{{\mathcal{N}}_{\overline{\overline{{I}_{j}}}}}}}^{L} \right) ~ \right) }^{\psi } \right) }^{ \frac{1}{\psi } }} \right) , \left( {e}^{-{ \left( \sum _{j=1}^{n}\overline{\overline{{\mathfrak{W}}_{j}}}{ \left( -\text{log} \left( {\overline{\overline{{\mathcal{N}}_{\overline{\overline{{I}_{j}}}}}}}^{U} \right) ~ \right) }^{\psi } \right) }^{ \frac{1}{\psi } }} \right) \right] } \end{array} \right) \end{eqnarray*}

\begin{eqnarray*}= \left( \begin{array}{@{}c@{}} \displaystyle \left[ \left( 1-{e}^{-{ \left( { \left( -\text{log} \left( 1-{\overline{\overline{{\mathcal{M}}_{\overline{\overline{R}}}}}}^{L} \right) ~ \right) }^{\psi } \right) }^{ \frac{1}{\psi } }} \right) , \left( 1-{e}^{-{ \left( { \left( -\text{log} \left( 1-{\overline{\overline{{\mathcal{M}}_{\overline{\overline{R}}}}}}^{U} \right) ~ \right) }^{\psi } \right) }^{ \frac{1}{\psi } }} \right) \right] \\ \displaystyle {e}^{i2\pi \left[ \left( 1-{e}^{-{ \left( { \left( -\text{log} \left( 1-{\overline{\overline{{\mathcal{M}}_{\overline{\overline{I}}}}}}^{L} \right) ~ \right) }^{\psi } \right) }^{ \frac{1}{\psi } }} \right) , \left( 1-{e}^{-{ \left( { \left( -\text{log} \left( 1-{\overline{\overline{{\mathcal{M}}_{\overline{\overline{I}}}}}}^{U} \right) ~ \right) }^{\psi } \right) }^{ \frac{1}{\psi } }} \right) \right] },\\ \displaystyle \left[ \left( {e}^{-{ \left( { \left( -\text{log} \left( {\overline{\overline{{\mathcal{N}}_{\overline{\overline{R}}}}}}^{L} \right) ~ \right) }^{\psi } \right) }^{ \frac{1}{\psi } }} \right) , \left( {e}^{-{ \left( { \left( -\text{log} \left( {\overline{\overline{{\mathcal{N}}_{\overline{\overline{R}}}}}}^{U} \right) ~ \right) }^{\psi } \right) }^{ \frac{1}{\psi } }} \right) \right] \\ \displaystyle {e}^{i2\pi \left[ \left( {e}^{-{ \left( { \left( -\text{log} \left( {\overline{\overline{{\mathcal{N}}_{\overline{\overline{I}}}}}}^{L} \right) ~ \right) }^{\psi } \right) }^{ \frac{1}{\psi } }} \right) , \left( {e}^{-{ \left( { \left( -\text{log} \left( {\overline{\overline{{\mathcal{N}}_{\overline{\overline{I}}}}}}^{U} \right) ~ \right) }^{\psi } \right) }^{ \frac{1}{\psi } }} \right) \right] } \end{array} \right) \end{eqnarray*}

\begin{eqnarray*}= \left( \begin{array}{@{}c@{}} \displaystyle \left[ \left( 1-{e}^{\text{log} \left( 1-{\overline{\overline{{\mathcal{M}}_{\overline{\overline{R}}}}}}^{L} \right) ~} \right) , \left( 1-{e}^{\text{log} \left( 1-{\overline{\overline{{\mathcal{M}}_{\overline{\overline{R}}}}}}^{U} \right) ~} \right) \right] {e}^{i2\pi \left[ \left( 1-{e}^{\text{log} \left( 1-{\overline{\overline{{\mathcal{M}}_{\overline{\overline{I}}}}}}^{L} \right) ~} \right) , \left( 1-{e}^{\text{log} \left( 1-{\overline{\overline{{\mathcal{M}}_{\overline{\overline{I}}}}}}^{U} \right) ~} \right) \right] },\\ \displaystyle \left[ \left( {e}^{\text{log} \left( {\overline{\overline{{\mathcal{N}}_{\overline{\overline{R}}}}}}^{L} \right) ~} \right) , \left( {e}^{\text{log} \left( {\overline{\overline{{\mathcal{N}}_{\overline{\overline{R}}}}}}^{U} \right) ~} \right) \right] {e}^{i2\pi \left[ \left( {e}^{\text{log} \left( {\overline{\overline{{\mathcal{N}}_{\overline{\overline{I}}}}}}^{L} \right) ~} \right) , \left( {e}^{\text{log} \left( {\overline{\overline{{\mathcal{N}}_{\overline{\overline{I}}}}}}^{U} \right) ~} \right) \right] } \end{array} \right) \end{eqnarray*}

\begin{eqnarray*}= \left( \left[ {\overline{\overline{{\mathcal{M}}_{\overline{\overline{R}}}}}}^{L},{\overline{\overline{{\mathcal{M}}_{\overline{\overline{R}}}}}}^{U} \right] {e}^{i2\pi \left( \left[ {\overline{\overline{{\mathcal{M}}_{\overline{\overline{I}}}}}}^{L},{\overline{\overline{{\mathcal{M}}_{\overline{\overline{I}}}}}}^{U} \right] \right) }, \left[ {\overline{\overline{{\mathcal{N}}_{\overline{\overline{R}}}}}}^{L},{\overline{\overline{{\mathcal{N}}_{\overline{\overline{R}}}}}}^{U} \right] {e}^{i2\pi \left( \left[ {\overline{\overline{{\mathcal{N}}_{\overline{\overline{I}}}}}}^{L},{\overline{\overline{{\mathcal{N}}_{\overline{\overline{I}}}}}}^{U} \right] \right) } \right) . \end{eqnarray*}

**Property 2:** For CIVIFNs $\overline{\overline{{\mathfrak{C}}_{{C}_{j}}}}= \left( {\scriptsize \begin{array}{@{}c@{}} \displaystyle \left[ {\overline{\overline{{\mathcal{M}}_{\overline{\overline{{R}_{j}}}}}}}^{L} \left( \widetilde {{x}_{E}} \right) ,{\overline{\overline{{\mathcal{M}}_{\overline{\overline{{R}_{j}}}}}}}^{U} \left( \widetilde {{x}_{E}} \right) \right] {e}^{i2\pi \left( \left[ {\overline{\overline{{\mathcal{M}}_{\overline{\overline{{I}_{j}}}}}}}^{L} \left( \widetilde {{x}_{E}} \right) ,{\overline{\overline{{\mathcal{M}}_{\overline{\overline{{I}_{j}}}}}}}^{U} \left( \widetilde {{x}_{E}} \right) \right] \right) },\\ \displaystyle \left[ {\overline{\overline{{\mathcal{N}}_{\overline{\overline{{R}_{j}}}}}}}^{L} \left( \widetilde {{x}_{E}} \right) ,{\overline{\overline{{\mathcal{N}}_{\overline{\overline{{R}_{j}}}}}}}^{U} \left( \widetilde {{x}_{E}} \right) \right] {e}^{i2\pi \left( \left[ {\overline{\overline{{\mathcal{N}}_{\overline{\overline{{I}_{j}}}}}}}^{L} \left( \widetilde {{x}_{E}} \right) ,{\overline{\overline{{\mathcal{N}}_{\overline{\overline{{I}_{j}}}}}}}^{U} \left( \widetilde {{x}_{E}} \right) \right] \right) } \end{array}} \right) ,j=1,2,\ldots ,n$, if $\overline{\overline{{\mathfrak{C}}^{-}}}= \left( \left[ {\min }_{j}{\overline{\overline{{\mathcal{M}}_{\overline{\overline{{R}_{j}}}}}}}^{L}~,{\min }_{j}{\overline{\overline{{\mathcal{M}}_{\overline{\overline{{R}_{j}}}}}}}^{U}~ \right] {e}^{i2\pi \left[ {\min }_{j}{\overline{\overline{{\mathcal{M}}_{\overline{\overline{{I}_{j}}}}}}}^{L}~,{\min }_{j}{\overline{\overline{{\mathcal{M}}_{\overline{\overline{{I}_{j}}}}}}}^{U}~ \right] } \right. $,

$ \left. \left[ {\max }_{j}{\overline{\overline{{\mathcal{N}}_{\overline{\overline{{R}_{j}}}}}}}^{L}~,{\max }_{j}{\overline{\overline{{\mathcal{N}}_{\overline{\overline{{R}_{j}}}}}}}^{U}~ \right] {e}^{i2\pi \left[ {\max }_{j}{\overline{\overline{{\mathcal{N}}_{\overline{\overline{{I}_{j}}}}}}}^{L}~,{\max }_{j}{\overline{\overline{{\mathcal{N}}_{\overline{\overline{{I}_{j}}}}}}}^{U}~ \right] } \right) $ and ${\overline{\overline{\mathfrak{C}}}}^{+}= \left( \left[ {\max }_{j}{\overline{\overline{{\mathcal{M}}_{\overline{\overline{{R}_{j}}}}}}}^{L}~,{\max }_{j}{\overline{\overline{{\mathcal{M}}_{\overline{\overline{{R}_{j}}}}}}}^{U}~ \right] {e}^{i2\pi \left[ {\max }_{j}{\overline{\overline{{\mathcal{M}}_{\overline{\overline{{I}_{j}}}}}}}^{L}~,{\max }_{j}{\overline{\overline{{\mathcal{M}}_{\overline{\overline{{I}_{j}}}}}}}^{U}~ \right] }, \left[ {\min }_{j}{\overline{\overline{{\mathcal{N}}_{\overline{\overline{{R}_{j}}}}}}}^{L}~,{\min }_{j}{\overline{\overline{{\mathcal{N}}_{\overline{\overline{{R}_{j}}}}}}}^{U}~ \right] \right. $

$ \left. {e}^{i2\pi \left[ {\min }_{j}{\overline{\overline{{\mathcal{N}}_{\overline{\overline{{I}_{j}}}}}}}^{L}~,{\min }_{j}{\overline{\overline{{\mathcal{N}}_{\overline{\overline{{I}_{j}}}}}}}^{U}~ \right] } \right) $, then

\begin{eqnarray*}{\overline{\overline{\mathfrak{C}}}}^{-}\leq CIVIFAAWA \left( \overline{\overline{{\mathfrak{C}}_{{C}_{1}}}},\overline{\overline{{\mathfrak{C}}_{{C}_{2}}}},\ldots ,\overline{\overline{{\mathfrak{C}}_{{C}_{n}}}} \right) \leq {\overline{\overline{\mathfrak{C}}}}^{+} \end{eqnarray*}

**Proof:** Suppose $\overline{\overline{{\mathfrak{C}}_{{C}_{j}}}}= \left( {\scriptsize \begin{array}{@{}c@{}} \displaystyle \left[ {\overline{\overline{{\mathcal{M}}_{\overline{\overline{{R}_{j}}}}}}}^{L} \left( \widetilde {{x}_{E}} \right) ,{\overline{\overline{{\mathcal{M}}_{\overline{\overline{{R}_{j}}}}}}}^{U} \left( \widetilde {{x}_{E}} \right) \right] {e}^{i2\pi \left( \left[ {\overline{\overline{{\mathcal{M}}_{\overline{\overline{{I}_{j}}}}}}}^{L} \left( \widetilde {{x}_{E}} \right) ,{\overline{\overline{{\mathcal{M}}_{\overline{\overline{{I}_{j}}}}}}}^{U} \left( \widetilde {{x}_{E}} \right) \right] \right) },\\ \displaystyle \left[ {\overline{\overline{{\mathcal{N}}_{\overline{\overline{{R}_{j}}}}}}}^{L} \left( \widetilde {{x}_{E}} \right) ,{\overline{\overline{{\mathcal{N}}_{\overline{\overline{{R}_{j}}}}}}}^{U} \left( \widetilde {{x}_{E}} \right) \right] {e}^{i2\pi \left( \left[ {\overline{\overline{{\mathcal{N}}_{\overline{\overline{{I}_{j}}}}}}}^{L} \left( \widetilde {{x}_{E}} \right) ,{\overline{\overline{{\mathcal{N}}_{\overline{\overline{{I}_{j}}}}}}}^{U} \left( \widetilde {{x}_{E}} \right) \right] \right) } \end{array}} \right) $, if

${\overline{\overline{\mathfrak{C}}}}^{-}= \left( \left[ {\min }_{j}{\overline{\overline{{\mathcal{M}}_{\overline{\overline{{R}_{j}}}}}}}^{L}~,{\min }_{j}{\overline{\overline{{\mathcal{M}}_{\overline{\overline{{R}_{j}}}}}}}^{U}~ \right] {e}^{i2\pi \left[ {\min }_{j}{\overline{\overline{{\mathcal{M}}_{\overline{\overline{{I}_{j}}}}}}}^{L}~,{\min }_{j}{\overline{\overline{{\mathcal{M}}_{\overline{\overline{{I}_{j}}}}}}}^{U}~ \right] }, \left[ {\max }_{j}{\overline{\overline{{\mathcal{N}}_{\overline{\overline{{R}_{j}}}}}}}^{L}~,{\max }_{j}{\overline{\overline{{\mathcal{N}}_{\overline{\overline{{R}_{j}}}}}}}^{U}~ \right] \right. $

$ \left. {e}^{i2\pi \left[ {\max }_{j}{\overline{\overline{{\mathcal{N}}_{\overline{\overline{{I}_{j}}}}}}}^{L}~,{\max }_{j}{\overline{\overline{{\mathcal{N}}_{\overline{\overline{{I}_{j}}}}}}}^{U}~ \right] } \right) $ and

${\overline{\overline{\mathfrak{C}}}}^{+}= \left( \left[ {\max }_{j}{\overline{\overline{{\mathcal{M}}_{\overline{\overline{{R}_{j}}}}}}}^{L}~,{\max }_{j}{\overline{\overline{{\mathcal{M}}_{\overline{\overline{{R}_{j}}}}}}}^{U}~ \right] {e}^{i2\pi \left[ {\max }_{j}{\overline{\overline{{\mathcal{M}}_{\overline{\overline{{I}_{j}}}}}}}^{L}~,{\max }_{j}{\overline{\overline{{\mathcal{M}}_{\overline{\overline{{I}_{j}}}}}}}^{U}~ \right] }, \left[ {\min }_{j}{\overline{\overline{{\mathcal{N}}_{\overline{\overline{{R}_{j}}}}}}}^{L}~,{\min }_{j}{\overline{\overline{{\mathcal{N}}_{\overline{\overline{{R}_{j}}}}}}}^{U}~ \right] \right. $

$ \left. {e}^{i2\pi \left[ {\min }_{j}{\overline{\overline{{\mathcal{N}}_{\overline{\overline{{I}_{j}}}}}}}^{L}~,{\min }_{j}{\overline{\overline{{\mathcal{N}}_{\overline{\overline{{I}_{j}}}}}}}^{U}~ \right] } \right) $, by using inequality, we have

\begin{eqnarray*}1-{e}^{-{ \left( \sum _{j=1}^{n}\overline{\overline{{\mathfrak{W}}_{j}}}{ \left( -\text{log} \left( 1-{{\overline{\overline{{\mathcal{M}}_{\overline{\overline{{R}_{j}}}}}}}^{-}}^{L} \right) ~ \right) }^{\psi } \right) }^{ \frac{1}{\psi } }}\leq 1-{e}^{-{ \left( \sum _{j=1}^{n}\overline{\overline{{\mathfrak{W}}_{j}}}{ \left( -\text{log} \left( 1-{\overline{\overline{{\mathcal{M}}_{\overline{\overline{{R}_{j}}}}}}}^{L} \right) ~ \right) }^{\psi } \right) }^{ \frac{1}{\psi } }}\leq 1-{e}^{-{ \left( \sum _{j=1}^{n}\overline{\overline{{\mathfrak{W}}_{j}}}{ \left( -\text{log} \left( 1-{{\overline{\overline{{\mathcal{M}}_{\overline{\overline{{R}_{j}}}}}}}^{+}}^{L} \right) ~ \right) }^{\psi } \right) }^{ \frac{1}{\psi } }} \end{eqnarray*}

\begin{eqnarray*}1-{e}^{-{ \left( \sum _{j=1}^{n}\overline{\overline{{\mathfrak{W}}_{j}}}{ \left( -\text{log} \left( 1-{{\overline{\overline{{\mathcal{M}}_{\overline{\overline{{I}_{j}}}}}}}^{-}}^{L} \right) ~ \right) }^{\psi } \right) }^{ \frac{1}{\psi } }}\leq 1-{e}^{-{ \left( \sum _{j=1}^{n}\overline{\overline{{\mathfrak{W}}_{j}}}{ \left( -\text{log} \left( 1-{\overline{\overline{{\mathcal{M}}_{\overline{\overline{{I}_{j}}}}}}}^{L} \right) ~ \right) }^{\psi } \right) }^{ \frac{1}{\psi } }}\leq 1-{e}^{-{ \left( \sum _{j=1}^{n}\overline{\overline{{\mathfrak{W}}_{j}}}{ \left( -\text{log} \left( 1-{{\overline{\overline{{\mathcal{M}}_{\overline{\overline{{I}_{j}}}}}}}^{+}}^{L} \right) ~ \right) }^{\psi } \right) }^{ \frac{1}{\psi } }} \end{eqnarray*}

\begin{eqnarray*}{e}^{-{ \left( \sum _{j=1}^{n}\overline{\overline{{\mathfrak{W}}_{j}}}{ \left( -\text{log} \left( {{\overline{\overline{{\mathcal{M}}_{\overline{\overline{{R}_{j}}}}}}}^{-}}^{L} \right) ~ \right) }^{\psi } \right) }^{ \frac{1}{\psi } }}\leq {e}^{-{ \left( \sum _{j=1}^{n}\overline{\overline{{\mathfrak{W}}_{j}}}{ \left( -\text{log} \left( {\overline{\overline{{\mathcal{M}}_{\overline{\overline{{R}_{j}}}}}}}^{L} \right) ~ \right) }^{\psi } \right) }^{ \frac{1}{\psi } }}\leq {e}^{-{ \left( \sum _{j=1}^{n}\overline{\overline{{\mathfrak{W}}_{j}}}{ \left( -\text{log} \left( {{\overline{\overline{{\mathcal{M}}_{\overline{\overline{{R}_{j}}}}}}}^{+}}^{L} \right) ~ \right) }^{\psi } \right) }^{ \frac{1}{\psi } }} \end{eqnarray*}

\begin{eqnarray*}{e}^{-{ \left( \sum _{j=1}^{n}\overline{\overline{{\mathfrak{W}}_{j}}}{ \left( -\text{log} \left( {{\overline{\overline{{\mathcal{M}}_{\overline{\overline{{I}_{j}}}}}}}^{-}}^{L} \right) ~ \right) }^{\psi } \right) }^{ \frac{1}{\psi } }}\leq {e}^{-{ \left( \sum _{j=1}^{n}\overline{\overline{{\mathfrak{W}}_{j}}}{ \left( -\text{log} \left( {\overline{\overline{{\mathcal{M}}_{\overline{\overline{{I}_{j}}}}}}}^{L} \right) ~ \right) }^{\psi } \right) }^{ \frac{1}{\psi } }}\leq {e}^{-{ \left( \sum _{j=1}^{n}\overline{\overline{{\mathfrak{W}}_{j}}}{ \left( -\text{log} \left( {{\overline{\overline{{\mathcal{M}}_{\overline{\overline{{I}_{j}}}}}}}^{+}}^{L} \right) ~ \right) }^{\psi } \right) }^{ \frac{1}{\psi } }} \end{eqnarray*}

Similarly, for the upper part, we have

\begin{eqnarray*}{e}^{-{ \left( \sum _{j=1}^{n}\overline{\overline{{\mathfrak{W}}_{j}}}{ \left( -\text{log} \left( {{\overline{\overline{{\mathcal{M}}_{\overline{\overline{{I}_{j}}}}}}}^{-}}^{U} \right) ~ \right) }^{\psi } \right) }^{ \frac{1}{\psi } }}\leq {e}^{-{ \left( \sum _{j=1}^{n}\overline{\overline{{\mathfrak{W}}_{j}}}{ \left( -\text{log} \left( {\overline{\overline{{\mathcal{M}}_{\overline{\overline{{I}_{j}}}}}}}^{U} \right) ~ \right) }^{\psi } \right) }^{ \frac{1}{\psi } }}\leq {e}^{-{ \left( \sum _{j=1}^{n}\overline{\overline{{\mathfrak{W}}_{j}}}{ \left( -\text{log} \left( {{\overline{\overline{{\mathcal{M}}_{\overline{\overline{{I}_{j}}}}}}}^{+}}^{U} \right) ~ \right) }^{\psi } \right) }^{ \frac{1}{\psi } }} \end{eqnarray*}

Therefore, we obtained

\begin{eqnarray*}\overline{\overline{{\mathfrak{C}}^{-}}}\leq CIVIFAAWA \left( \overline{\overline{{\mathfrak{C}}_{{C}_{1}}}},\overline{\overline{{\mathfrak{C}}_{{C}_{2}}}},\ldots ,\overline{\overline{{\mathfrak{C}}_{{C}_{n}}}} \right) \leq \overline{\overline{{\mathfrak{C}}^{+}}}. \end{eqnarray*}

**Property 3:** For CIVIFNs $\overline{\overline{{\mathfrak{C}}_{{C}_{j}}}}= \left( {\scriptsize \begin{array}{@{}c@{}} \displaystyle \left[ {\overline{\overline{{\mathcal{M}}_{\overline{\overline{{R}_{j}}}}}}}^{L} \left( \widetilde {{x}_{E}} \right) ,{\overline{\overline{{\mathcal{M}}_{\overline{\overline{{R}_{j}}}}}}}^{U} \left( \widetilde {{x}_{E}} \right) \right] {e}^{i2\pi \left( \left[ {\overline{\overline{{\mathcal{M}}_{\overline{\overline{{I}_{j}}}}}}}^{L} \left( \widetilde {{x}_{E}} \right) ,{\overline{\overline{{\mathcal{M}}_{\overline{\overline{{I}_{j}}}}}}}^{U} \left( \widetilde {{x}_{E}} \right) \right] \right) },\\ \displaystyle \left[ {\overline{\overline{{\mathcal{N}}_{\overline{\overline{{R}_{j}}}}}}}^{L} \left( \widetilde {{x}_{E}} \right) ,{\overline{\overline{{\mathcal{N}}_{\overline{\overline{{R}_{j}}}}}}}^{U} \left( \widetilde {{x}_{E}} \right) \right] {e}^{i2\pi \left( \left[ {\overline{\overline{{\mathcal{N}}_{\overline{\overline{{I}_{j}}}}}}}^{L} \left( \widetilde {{x}_{E}} \right) ,{\overline{\overline{{\mathcal{N}}_{\overline{\overline{{I}_{j}}}}}}}^{U} \left( \widetilde {{x}_{E}} \right) \right] \right) } \end{array}} \right) ,j=1,\ldots ,n$, if $\overline{\overline{{\mathfrak{C}}_{{C}_{j}}}}\leq {\overline{\overline{{\mathfrak{C}}_{{C}_{j}}}}}^{{}^{{^{\prime}}}}$, then

\begin{eqnarray*}CIVIFAAWA \left( \overline{\overline{{\mathfrak{C}}_{{C}_{1}}}},\overline{\overline{{\mathfrak{C}}_{{C}_{2}}}},\ldots ,\overline{\overline{{\mathfrak{C}}_{{C}_{n}}}} \right) \leq CIVIFAAWA \left( {\overline{\overline{{\mathfrak{C}}_{{C}_{1}}}}}^{{}^{{^{\prime}}}},{\overline{\overline{{\mathfrak{C}}_{{C}_{2}}}}}^{{}^{{^{\prime}}}},\ldots ,{\overline{\overline{{\mathfrak{C}}_{{C}_{n}}}}}^{{}^{{^{\prime}}}} \right) \end{eqnarray*}

**Definition 12:** For CIVIFNs $\overline{\overline{{\mathfrak{C}}_{{C}_{j}}}}= \left( {\scriptsize \begin{array}{@{}c@{}} \displaystyle \left[ {\overline{\overline{{\mathcal{M}}_{\overline{\overline{{R}_{j}}}}}}}^{L} \left( \widetilde {{x}_{E}} \right) ,{\overline{\overline{{\mathcal{M}}_{\overline{\overline{{R}_{j}}}}}}}^{U} \left( \widetilde {{x}_{E}} \right) \right] {e}^{i2\pi \left( \left[ {\overline{\overline{{\mathcal{M}}_{\overline{\overline{{I}_{j}}}}}}}^{L} \left( \widetilde {{x}_{E}} \right) ,{\overline{\overline{{\mathcal{M}}_{\overline{\overline{{I}_{j}}}}}}}^{U} \left( \widetilde {{x}_{E}} \right) \right] \right) },\\ \displaystyle \left[ {\overline{\overline{{\mathcal{N}}_{\overline{\overline{{R}_{j}}}}}}}^{L} \left( \widetilde {{x}_{E}} \right) ,{\overline{\overline{{\mathcal{N}}_{\overline{\overline{{R}_{j}}}}}}}^{U} \left( \widetilde {{x}_{E}} \right) \right] {e}^{i2\pi \left( \left[ {\overline{\overline{{\mathcal{N}}_{\overline{\overline{{I}_{j}}}}}}}^{L} \left( \widetilde {{x}_{E}} \right) ,{\overline{\overline{{\mathcal{N}}_{\overline{\overline{{I}_{j}}}}}}}^{U} \left( \widetilde {{x}_{E}} \right) \right] \right) } \end{array}} \right) ,j=1,\ldots ,n$, then the CIVIFAAOWA operator is invented by:

(25) \begin{eqnarray*}CIVIFAAOWA \left( \overline{\overline{{\mathfrak{C}}_{{C}_{1}}}},\overline{\overline{{\mathfrak{C}}_{{C}_{2}}}},\ldots ,\overline{\overline{{\mathfrak{C}}_{{C}_{n}}}} \right) =\overline{\overline{{\mathfrak{W}}_{1}}}\overline{\overline{{\mathfrak{C}}_{{C}_{\varphi \left( 1 \right) }}}}\oplus \overline{\overline{{\mathfrak{W}}_{2}}}\overline{\overline{{\mathfrak{C}}_{{C}_{\varphi \left( 2 \right) }}}}\oplus \cdots \oplus \overline{\overline{{\mathfrak{W}}_{n}}}\overline{\overline{{\mathfrak{C}}_{{C}_{\varphi \left( n \right) }}}}={\mathop{\oplus \nolimits }\nolimits }_{j=1}^{n} \left( \overline{\overline{{\mathfrak{W}}_{j}}}\overline{\overline{{\mathfrak{C}}_{{C}_{\varphi \left( j \right) }}}} \right) \end{eqnarray*}

where $\overline{\overline{\mathfrak{W}}}={ \left( \overline{\overline{{\mathfrak{W}}_{1}}},\overline{\overline{{\mathfrak{W}}_{2}}},\ldots ,\overline{\overline{{\mathfrak{W}}_{n}}} \right) }^{T}$ indicates the weight of *C*_*j*_, with ${\mathop{\sum }\nolimits }_{j=1}^{n}\overline{\overline{{\mathfrak{W}}_{j}}}=1$, with parameter $\varphi \left( 1 \right) ,\varphi \left( 2 \right) ,\ldots ,\varphi \left( n \right) $ based on $\overline{\overline{{\mathfrak{C}}_{{C}_{\varphi \left( j \right) }}}}\leq \overline{\overline{{\mathfrak{C}}_{{C}_{\varphi \left( j-1 \right) }}}}$.

**Theorem 2:** For CIVIFNs $\overline{\overline{{\mathfrak{C}}_{{C}_{j}}}}= \left( {\scriptsize \begin{array}{@{}c@{}} \displaystyle \left[ {\overline{\overline{{\mathcal{M}}_{\overline{\overline{{R}_{j}}}}}}}^{L} \left( \widetilde {{x}_{E}} \right) ,{\overline{\overline{{\mathcal{M}}_{\overline{\overline{{R}_{j}}}}}}}^{U} \left( \widetilde {{x}_{E}} \right) \right] {e}^{i2\pi \left( \left[ {\overline{\overline{{\mathcal{M}}_{\overline{\overline{{I}_{j}}}}}}}^{L} \left( \widetilde {{x}_{E}} \right) ,{\overline{\overline{{\mathcal{M}}_{\overline{\overline{{I}_{j}}}}}}}^{U} \left( \widetilde {{x}_{E}} \right) \right] \right) },\\ \displaystyle \left[ {\overline{\overline{{\mathcal{N}}_{\overline{\overline{{R}_{j}}}}}}}^{L} \left( \widetilde {{x}_{E}} \right) ,{\overline{\overline{{\mathcal{N}}_{\overline{\overline{{R}_{j}}}}}}}^{U} \left( \widetilde {{x}_{E}} \right) \right] {e}^{i2\pi \left( \left[ {\overline{\overline{{\mathcal{N}}_{\overline{\overline{{I}_{j}}}}}}}^{L} \left( \widetilde {{x}_{E}} \right) ,{\overline{\overline{{\mathcal{N}}_{\overline{\overline{{I}_{j}}}}}}}^{U} \left( \widetilde {{x}_{E}} \right) \right] \right) } \end{array}} \right) ,j=1,\ldots ,n$, then by using Eq. (25), we elaborate

(26) \begin{eqnarray*}CIVIFAAOWA \left( \overline{\overline{{\mathfrak{C}}_{{C}_{1}}}},\overline{\overline{{\mathfrak{C}}_{{C}_{2}}}},\ldots ,\overline{\overline{{\mathfrak{C}}_{{C}_{n}}}} \right) \nonumber\\\displaystyle = \left( \begin{array}{@{}c@{}} \displaystyle \left[ \left( 1-{e}^{-{ \left( \sum _{j=1}^{n}\overline{\overline{{\mathfrak{W}}_{j}}}{ \left( -\text{log} \left( 1-{\overline{\overline{{\mathcal{M}}_{\overline{\overline{{R}_{\varphi \left( j \right) }}}}}}}^{L} \right) ~ \right) }^{\psi } \right) }^{ \frac{1}{\psi } }} \right) , \left( 1-{e}^{-{ \left( \sum _{j=1}^{n}\overline{\overline{{\mathfrak{W}}_{j}}}{ \left( -\text{log} \left( 1-{\overline{\overline{{\mathcal{M}}_{\overline{\overline{{R}_{\varphi \left( j \right) }}}}}}}^{U} \right) ~ \right) }^{\psi } \right) }^{ \frac{1}{\psi } }} \right) \right] \\ \displaystyle {e}^{i2\pi \left[ \left( 1-{e}^{-{ \left( \sum _{j=1}^{n}\overline{\overline{{\mathfrak{W}}_{j}}}{ \left( -\text{log} \left( 1-{\overline{\overline{{\mathcal{M}}_{\overline{\overline{{I}_{\varphi \left( j \right) }}}}}}}^{L} \right) ~ \right) }^{\psi } \right) }^{ \frac{1}{\psi } }} \right) , \left( 1-{e}^{-{ \left( \sum _{j=1}^{n}\overline{\overline{{\mathfrak{W}}_{j}}}{ \left( -\text{log} \left( 1-{\overline{\overline{{\mathcal{M}}_{\overline{\overline{{I}_{\varphi \left( j \right) }}}}}}}^{U} \right) ~ \right) }^{\psi } \right) }^{ \frac{1}{\psi } }} \right) \right] },\\ \displaystyle \left[ \left( {e}^{-{ \left( \sum _{j=1}^{n}\overline{\overline{{\mathfrak{W}}_{j}}}{ \left( -\text{log} \left( {\overline{\overline{{\mathcal{N}}_{\overline{\overline{{R}_{\varphi \left( j \right) }}}}}}}^{L} \right) ~ \right) }^{\psi } \right) }^{ \frac{1}{\psi } }} \right) , \left( {e}^{-{ \left( \sum _{j=1}^{n}\overline{\overline{{\mathfrak{W}}_{j}}}{ \left( -\text{log} \left( {\overline{\overline{{\mathcal{N}}_{\overline{\overline{{R}_{\varphi \left( j \right) }}}}}}}^{U} \right) ~ \right) }^{\psi } \right) }^{ \frac{1}{\psi } }} \right) \right] \\ \displaystyle {e}^{i2\pi \left[ \left( {e}^{-{ \left( \sum _{j=1}^{n}\overline{\overline{{\mathfrak{W}}_{j}}}{ \left( -\text{log} \left( {\overline{\overline{{\mathcal{N}}_{\overline{\overline{{I}_{\varphi \left( j \right) }}}}}}}^{L} \right) ~ \right) }^{\psi } \right) }^{ \frac{1}{\psi } }} \right) , \left( {e}^{-{ \left( \sum _{j=1}^{n}\overline{\overline{{\mathfrak{W}}_{j}}}{ \left( -\text{log} \left( {\overline{\overline{{\mathcal{N}}_{\overline{\overline{{I}_{\varphi \left( j \right) }}}}}}}^{U} \right) ~ \right) }^{\psi } \right) }^{ \frac{1}{\psi } }} \right) \right] } \end{array} \right) \end{eqnarray*}

**Idempotency-Property 4:** By taking CIVIFNs $\overline{\overline{{\mathfrak{C}}_{{C}_{j}}}}= \left( {\scriptsize \begin{array}{@{}c@{}} \displaystyle \left[ {\overline{\overline{{\mathcal{M}}_{\overline{\overline{{R}_{j}}}}}}}^{L} \left( \widetilde {{x}_{E}} \right) ,{\overline{\overline{{\mathcal{M}}_{\overline{\overline{{R}_{j}}}}}}}^{U} \left( \widetilde {{x}_{E}} \right) \right] {e}^{i2\pi \left( \left[ {\overline{\overline{{\mathcal{M}}_{\overline{\overline{{I}_{j}}}}}}}^{L} \left( \widetilde {{x}_{E}} \right) ,{\overline{\overline{{\mathcal{M}}_{\overline{\overline{{I}_{j}}}}}}}^{U} \left( \widetilde {{x}_{E}} \right) \right] \right) },\\ \displaystyle \left[ {\overline{\overline{{\mathcal{N}}_{\overline{\overline{{R}_{j}}}}}}}^{L} \left( \widetilde {{x}_{E}} \right) ,{\overline{\overline{{\mathcal{N}}_{\overline{\overline{{R}_{j}}}}}}}^{U} \left( \widetilde {{x}_{E}} \right) \right] {e}^{i2\pi \left( \left[ {\overline{\overline{{\mathcal{N}}_{\overline{\overline{{I}_{j}}}}}}}^{L} \left( \widetilde {{x}_{E}} \right) ,{\overline{\overline{{\mathcal{N}}_{\overline{\overline{{I}_{j}}}}}}}^{U} \left( \widetilde {{x}_{E}} \right) \right] \right) } \end{array}} \right) ,j=1,2,\ldots ,n$, if $\overline{\overline{{\mathfrak{C}}_{{C}_{j}}}}=\overline{\overline{\mathfrak{C}}}$, then

(27) \begin{eqnarray*}CIVIFAAOWA \left( \overline{\overline{{\mathfrak{C}}_{{C}_{1}}}},\overline{\overline{{\mathfrak{C}}_{{C}_{2}}}},\ldots ,\overline{\overline{{\mathfrak{C}}_{{C}_{n}}}} \right) =\overline{\overline{\mathfrak{C}}}\end{eqnarray*}

**Monotonicity-Property 5:** By taking CIVIFNs $\overline{\overline{{\mathfrak{C}}_{{C}_{j}}}}= \left( {\scriptsize \begin{array}{@{}c@{}} \displaystyle \left[ {\overline{\overline{{\mathcal{M}}_{\overline{\overline{{R}_{j}}}}}}}^{L} \left( \widetilde {{x}_{E}} \right) ,{\overline{\overline{{\mathcal{M}}_{\overline{\overline{{R}_{j}}}}}}}^{U} \left( \widetilde {{x}_{E}} \right) \right] {e}^{i2\pi \left( \left[ {\overline{\overline{{\mathcal{M}}_{\overline{\overline{{I}_{j}}}}}}}^{L} \left( \widetilde {{x}_{E}} \right) ,{\overline{\overline{{\mathcal{M}}_{\overline{\overline{{I}_{j}}}}}}}^{U} \left( \widetilde {{x}_{E}} \right) \right] \right) },\\ \displaystyle \left[ {\overline{\overline{{\mathcal{N}}_{\overline{\overline{{R}_{j}}}}}}}^{L} \left( \widetilde {{x}_{E}} \right) ,{\overline{\overline{{\mathcal{N}}_{\overline{\overline{{R}_{j}}}}}}}^{U} \left( \widetilde {{x}_{E}} \right) \right] {e}^{i2\pi \left( \left[ {\overline{\overline{{\mathcal{N}}_{\overline{\overline{{I}_{j}}}}}}}^{L} \left( \widetilde {{x}_{E}} \right) ,{\overline{\overline{{\mathcal{N}}_{\overline{\overline{{I}_{j}}}}}}}^{U} \left( \widetilde {{x}_{E}} \right) \right] \right) } \end{array}} \right) ,j=1,2,\ldots ,n$, if ${\overline{\overline{\mathfrak{C}}}}^{-}= \left( \left[ {\min }_{j}{\overline{\overline{{\mathcal{M}}_{\overline{\overline{{R}_{j}}}}}}}^{L}~,{\min }_{j}{\overline{\overline{{\mathcal{M}}_{\overline{\overline{{R}_{j}}}}}}}^{U}~ \right] {e}^{i2\pi \left[ {\min }_{j}{\overline{\overline{{\mathcal{M}}_{\overline{\overline{{I}_{j}}}}}}}^{L}~,{\min }_{j}{\overline{\overline{{\mathcal{M}}_{\overline{\overline{{I}_{j}}}}}}}^{U}~ \right] } \right. $,

$ \left. \left[ {\max }_{j}{\overline{\overline{{\mathcal{N}}_{\overline{\overline{{R}_{j}}}}}}}^{L}~,{\max }_{j}{\overline{\overline{{\mathcal{N}}_{\overline{\overline{{R}_{j}}}}}}}^{U}~ \right] {e}^{i2\pi \left[ {\max }_{j}{\overline{\overline{{\mathcal{N}}_{\overline{\overline{{I}_{j}}}}}}}^{L}~,{\max }_{j}{\overline{\overline{{\mathcal{N}}_{\overline{\overline{{I}_{j}}}}}}}^{U}~ \right] } \right) $ and

${\overline{\overline{\mathfrak{C}}}}^{+}= \left( \left[ {\max }_{j}{\overline{\overline{{\mathcal{M}}_{\overline{\overline{{R}_{j}}}}}}}^{L}~,{\max }_{j}{\overline{\overline{{\mathcal{M}}_{\overline{\overline{{R}_{j}}}}}}}^{U}~ \right] {e}^{i2\pi \left[ {\max }_{j}{\overline{\overline{{\mathcal{M}}_{\overline{\overline{{I}_{j}}}}}}}^{L}~,{\max }_{j}{\overline{\overline{{\mathcal{M}}_{\overline{\overline{{I}_{j}}}}}}}^{U}~ \right] } \right. $,

$ \left. \left[ {\min }_{j}{\overline{\overline{{\mathcal{N}}_{\overline{\overline{{R}_{j}}}}}}}^{L}~,{\min }_{j}{\overline{\overline{{\mathcal{N}}_{\overline{\overline{{R}_{j}}}}}}}^{U}~ \right] {e}^{i2\pi \left[ {\min }_{j}{\overline{\overline{{\mathcal{N}}_{\overline{\overline{{I}_{j}}}}}}}^{L}~,{\min }_{j}{\overline{\overline{{\mathcal{N}}_{\overline{\overline{{I}_{j}}}}}}}^{U}~ \right] } \right) $, then

(28) \begin{eqnarray*}{\overline{\overline{\mathfrak{C}}}}^{-}\leq CIVIFAAOWA \left( \overline{\overline{{\mathfrak{C}}_{{C}_{1}}}},\overline{\overline{{\mathfrak{C}}_{{C}_{2}}}},\ldots ,\overline{\overline{{\mathfrak{C}}_{{C}_{n}}}} \right) \leq {\overline{\overline{\mathfrak{C}}}}^{+}\end{eqnarray*}

**Boundedness-Property 6:** By taking CIVIFNs

$\overline{\overline{{\mathfrak{C}}_{{C}_{j}}}}= \left( {\scriptsize \begin{array}{@{}c@{}} \displaystyle \left[ {\overline{\overline{{\mathcal{M}}_{\overline{\overline{{R}_{j}}}}}}}^{L} \left( \widetilde {{x}_{E}} \right) ,{\overline{\overline{{\mathcal{M}}_{\overline{\overline{{R}_{j}}}}}}}^{U} \left( \widetilde {{x}_{E}} \right) \right] {e}^{i2\pi \left( \left[ {\overline{\overline{{\mathcal{M}}_{\overline{\overline{{I}_{j}}}}}}}^{L} \left( \widetilde {{x}_{E}} \right) ,{\overline{\overline{{\mathcal{M}}_{\overline{\overline{{I}_{j}}}}}}}^{U} \left( \widetilde {{x}_{E}} \right) \right] \right) },\\ \displaystyle \left[ {\overline{\overline{{\mathcal{N}}_{\overline{\overline{{R}_{j}}}}}}}^{L} \left( \widetilde {{x}_{E}} \right) ,{\overline{\overline{{\mathcal{N}}_{\overline{\overline{{R}_{j}}}}}}}^{U} \left( \widetilde {{x}_{E}} \right) \right] {e}^{i2\pi \left( \left[ {\overline{\overline{{\mathcal{N}}_{\overline{\overline{{I}_{j}}}}}}}^{L} \left( \widetilde {{x}_{E}} \right) ,{\overline{\overline{{\mathcal{N}}_{\overline{\overline{{I}_{j}}}}}}}^{U} \left( \widetilde {{x}_{E}} \right) \right] \right) } \end{array}} \right) ,j=1,2,\ldots ,n$, if $\overline{\overline{{\mathfrak{C}}_{{C}_{j}}}}\leq {\overline{\overline{{\mathfrak{C}}_{{C}_{j}}}}}^{{}^{{^{\prime}}}}$, then

(29) \begin{eqnarray*}CIVIFAAOWA \left( \overline{\overline{{\mathfrak{C}}_{{C}_{1}}}},\overline{\overline{{\mathfrak{C}}_{{C}_{2}}}},\ldots ,\overline{\overline{{\mathfrak{C}}_{{C}_{n}}}} \right) \leq CIVIFAAOWA \left( {\overline{\overline{{\mathfrak{C}}_{{C}_{1}}}}}^{{}^{{^{\prime}}}},{\overline{\overline{{\mathfrak{C}}_{{C}_{2}}}}}^{{}^{{^{\prime}}}},\ldots ,{\overline{\overline{{\mathfrak{C}}_{{C}_{n}}}}}^{{}^{{^{\prime}}}} \right) \end{eqnarray*}

**Definition 13:** For CIVIFNs $\overline{\overline{{\mathfrak{C}}_{{C}_{j}}}}= \left( {\scriptsize \begin{array}{@{}c@{}} \displaystyle \left[ {\overline{\overline{{\mathcal{M}}_{\overline{\overline{{R}_{j}}}}}}}^{L} \left( \widetilde {{x}_{E}} \right) ,{\overline{\overline{{\mathcal{M}}_{\overline{\overline{{R}_{j}}}}}}}^{U} \left( \widetilde {{x}_{E}} \right) \right] {e}^{i2\pi \left( \left[ {\overline{\overline{{\mathcal{M}}_{\overline{\overline{{I}_{j}}}}}}}^{L} \left( \widetilde {{x}_{E}} \right) ,{\overline{\overline{{\mathcal{M}}_{\overline{\overline{{I}_{j}}}}}}}^{U} \left( \widetilde {{x}_{E}} \right) \right] \right) },\\ \displaystyle \left[ {\overline{\overline{{\mathcal{N}}_{\overline{\overline{{R}_{j}}}}}}}^{L} \left( \widetilde {{x}_{E}} \right) ,{\overline{\overline{{\mathcal{N}}_{\overline{\overline{{R}_{j}}}}}}}^{U} \left( \widetilde {{x}_{E}} \right) \right] {e}^{i2\pi \left( \left[ {\overline{\overline{{\mathcal{N}}_{\overline{\overline{{I}_{j}}}}}}}^{L} \left( \widetilde {{x}_{E}} \right) ,{\overline{\overline{{\mathcal{N}}_{\overline{\overline{{I}_{j}}}}}}}^{U} \left( \widetilde {{x}_{E}} \right) \right] \right) } \end{array}} \right) ,j=1,\ldots ,n$, then the CIVIFAAHA operator is invented by:

(30) \begin{eqnarray*}CIVIFAAHA \left( \overline{\overline{{\mathfrak{C}}_{{C}_{1}}}},\overline{\overline{{\mathfrak{C}}_{{C}_{2}}}},\ldots ,\overline{\overline{{\mathfrak{C}}_{{C}_{n}}}} \right) \nonumber\\\displaystyle =\overline{\overline{{\mathfrak{W}}_{1}}}\overline{\overline{\dot {{\mathfrak{C}}_{{C}_{\varphi \left( 1 \right) }}}}}\oplus \overline{\overline{{\mathfrak{W}}_{2}}}\overline{\overline{\dot {{\mathfrak{C}}_{{C}_{\varphi \left( 2 \right) }}}}}\oplus \cdots \oplus \overline{\overline{{\mathfrak{W}}_{n}}}\overline{\overline{\dot {{\mathfrak{C}}_{{C}_{\varphi \left( n \right) }}}}}={\mathop{\oplus \nolimits }\nolimits }_{j=1}^{n} \left( \overline{\overline{{\mathfrak{W}}_{j}}}\overline{\overline{\dot {{\mathfrak{C}}_{{C}_{\varphi \left( j \right) }}}}} \right) \end{eqnarray*}

**Theorem 3:** For CIVIFNs $\overline{\overline{{\mathfrak{C}}_{{C}_{j}}}}= \left( {\scriptsize \begin{array}{@{}c@{}} \displaystyle \left[ {\overline{\overline{{\mathcal{M}}_{\overline{\overline{{R}_{j}}}}}}}^{L} \left( \widetilde {{x}_{E}} \right) ,{\overline{\overline{{\mathcal{M}}_{\overline{\overline{{R}_{j}}}}}}}^{U} \left( \widetilde {{x}_{E}} \right) \right] {e}^{i2\pi \left( \left[ {\overline{\overline{{\mathcal{M}}_{\overline{\overline{{I}_{j}}}}}}}^{L} \left( \widetilde {{x}_{E}} \right) ,{\overline{\overline{{\mathcal{M}}_{\overline{\overline{{I}_{j}}}}}}}^{U} \left( \widetilde {{x}_{E}} \right) \right] \right) },\\ \displaystyle \left[ {\overline{\overline{{\mathcal{N}}_{\overline{\overline{{R}_{j}}}}}}}^{L} \left( \widetilde {{x}_{E}} \right) ,{\overline{\overline{{\mathcal{N}}_{\overline{\overline{{R}_{j}}}}}}}^{U} \left( \widetilde {{x}_{E}} \right) \right] {e}^{i2\pi \left( \left[ {\overline{\overline{{\mathcal{N}}_{\overline{\overline{{I}_{j}}}}}}}^{L} \left( \widetilde {{x}_{E}} \right) ,{\overline{\overline{{\mathcal{N}}_{\overline{\overline{{I}_{j}}}}}}}^{U} \left( \widetilde {{x}_{E}} \right) \right] \right) } \end{array}} \right) ,j=1,\ldots ,n$, then by using Eq. (30), we elaborate

(31) \begin{eqnarray*}CIVIFAAHA \left( \overline{\overline{{\mathfrak{C}}_{{C}_{1}}}},\overline{\overline{{\mathfrak{C}}_{{C}_{2}}}},\ldots ,\overline{\overline{{\mathfrak{C}}_{{C}_{n}}}} \right) \nonumber\\\displaystyle = \left( \begin{array}{@{}c@{}} \displaystyle \left[ \left( 1-{e}^{-{ \left( \sum _{j=1}^{n}\overline{\overline{{\mathfrak{W}}_{j}}}{ \left( -\text{log} \left( 1-{\overline{\overline{\dot {{\mathcal{M}}_{\overline{\overline{{R}_{\varphi \left( j \right) }}}}}}}}^{L} \right) ~ \right) }^{\psi } \right) }^{ \frac{1}{\psi } }} \right) , \left( 1-{e}^{-{ \left( \sum _{j=1}^{n}\overline{\overline{{\mathfrak{W}}_{j}}}{ \left( -\text{log} \left( 1-{\overline{\overline{\dot {{\mathcal{M}}_{\overline{\overline{{R}_{\varphi \left( j \right) }}}}}}}}^{U} \right) ~ \right) }^{\psi } \right) }^{ \frac{1}{\psi } }} \right) \right] \\ \displaystyle {e}^{i2\pi \left[ \left( 1-{e}^{-{ \left( \sum _{j=1}^{n}\overline{\overline{{\mathfrak{W}}_{j}}}{ \left( -\text{log} \left( 1-{\overline{\overline{\dot {{\mathcal{M}}_{\overline{\overline{{I}_{\varphi \left( j \right) }}}}}}}}^{L} \right) ~ \right) }^{\psi } \right) }^{ \frac{1}{\psi } }} \right) , \left( 1-{e}^{-{ \left( \sum _{j=1}^{n}\overline{\overline{{\mathfrak{W}}_{j}}}{ \left( -\text{log} \left( 1-{\overline{\overline{\dot {{\mathcal{M}}_{\overline{\overline{{I}_{\varphi \left( j \right) }}}}}}}}^{U} \right) ~ \right) }^{\psi } \right) }^{ \frac{1}{\psi } }} \right) \right] },\\ \displaystyle \left[ \left( {e}^{-{ \left( \sum _{j=1}^{n}\overline{\overline{{\mathfrak{W}}_{j}}}{ \left( -\text{log} \left( {\overline{\overline{\dot {{\mathcal{N}}_{\overline{\overline{{R}_{\varphi \left( j \right) }}}}}}}}^{L} \right) ~ \right) }^{\psi } \right) }^{ \frac{1}{\psi } }} \right) , \left( {e}^{-{ \left( \sum _{j=1}^{n}\overline{\overline{{\mathfrak{W}}_{j}}}{ \left( -\text{log} \left( {\overline{\overline{\dot {{\mathcal{N}}_{\overline{\overline{{R}_{\varphi \left( j \right) }}}}}}}}^{U} \right) ~ \right) }^{\psi } \right) }^{ \frac{1}{\psi } }} \right) \right] \\ \displaystyle {e}^{i2\pi \left[ \left( {e}^{-{ \left( \sum _{j=1}^{n}\overline{\overline{{\mathfrak{W}}_{j}}}{ \left( -\text{log} \left( {\overline{\overline{\dot {{\mathcal{N}}_{\overline{\overline{{I}_{\varphi \left( j \right) }}}}}}}}^{L} \right) ~ \right) }^{\psi } \right) }^{ \frac{1}{\psi } }} \right) , \left( {e}^{-{ \left( \sum _{j=1}^{n}\overline{\overline{{\mathfrak{W}}_{j}}}{ \left( -\text{log} \left( {\overline{\overline{\dot {{\mathcal{N}}_{\overline{\overline{{I}_{\varphi \left( j \right) }}}}}}}}^{U} \right) ~ \right) }^{\psi } \right) }^{ \frac{1}{\psi } }} \right) \right] } \end{array} \right) \end{eqnarray*}

**Idempotency-Property 7:** By taking CIVIFNs

$\overline{\overline{{\mathfrak{C}}_{{C}_{j}}}}= \left( {\scriptsize \begin{array}{@{}c@{}} \displaystyle \left[ {\overline{\overline{{\mathcal{M}}_{\overline{\overline{{R}_{j}}}}}}}^{L} \left( \widetilde {{x}_{E}} \right) ,{\overline{\overline{{\mathcal{M}}_{\overline{\overline{{R}_{j}}}}}}}^{U} \left( \widetilde {{x}_{E}} \right) \right] {e}^{i2\pi \left( \left[ {\overline{\overline{{\mathcal{M}}_{\overline{\overline{{I}_{j}}}}}}}^{L} \left( \widetilde {{x}_{E}} \right) ,{\overline{\overline{{\mathcal{M}}_{\overline{\overline{{I}_{j}}}}}}}^{U} \left( \widetilde {{x}_{E}} \right) \right] \right) },\\ \displaystyle \left[ {\overline{\overline{{\mathcal{N}}_{\overline{\overline{{R}_{j}}}}}}}^{L} \left( \widetilde {{x}_{E}} \right) ,{\overline{\overline{{\mathcal{N}}_{\overline{\overline{{R}_{j}}}}}}}^{U} \left( \widetilde {{x}_{E}} \right) \right] {e}^{i2\pi \left( \left[ {\overline{\overline{{\mathcal{N}}_{\overline{\overline{{I}_{j}}}}}}}^{L} \left( \widetilde {{x}_{E}} \right) ,{\overline{\overline{{\mathcal{N}}_{\overline{\overline{{I}_{j}}}}}}}^{U} \left( \widetilde {{x}_{E}} \right) \right] \right) } \end{array}} \right) ,j=1,2,\ldots ,n$, if $\overline{\overline{{\mathfrak{C}}_{{C}_{j}}}}=\overline{\overline{\mathfrak{C}}}$, then

(32) \begin{eqnarray*}CIVIFAAHA \left( \overline{\overline{{\mathfrak{C}}_{{C}_{1}}}},\overline{\overline{{\mathfrak{C}}_{{C}_{2}}}},\ldots ,\overline{\overline{{\mathfrak{C}}_{{C}_{n}}}} \right) =\overline{\overline{\mathfrak{C}}}\end{eqnarray*}

**Monotonicity-Property 8:** By taking CIVIFNs

$\overline{\overline{{\mathfrak{C}}_{{C}_{j}}}}= \left( {\scriptsize \begin{array}{@{}c@{}} \displaystyle \left[ {\overline{\overline{{\mathcal{M}}_{\overline{\overline{{R}_{j}}}}}}}^{L} \left( \widetilde {{x}_{E}} \right) ,{\overline{\overline{{\mathcal{M}}_{\overline{\overline{{R}_{j}}}}}}}^{U} \left( \widetilde {{x}_{E}} \right) \right] {e}^{i2\pi \left( \left[ {\overline{\overline{{\mathcal{M}}_{\overline{\overline{{I}_{j}}}}}}}^{L} \left( \widetilde {{x}_{E}} \right) ,{\overline{\overline{{\mathcal{M}}_{\overline{\overline{{I}_{j}}}}}}}^{U} \left( \widetilde {{x}_{E}} \right) \right] \right) },\\ \displaystyle \left[ {\overline{\overline{{\mathcal{N}}_{\overline{\overline{{R}_{j}}}}}}}^{L} \left( \widetilde {{x}_{E}} \right) ,{\overline{\overline{{\mathcal{N}}_{\overline{\overline{{R}_{j}}}}}}}^{U} \left( \widetilde {{x}_{E}} \right) \right] {e}^{i2\pi \left( \left[ {\overline{\overline{{\mathcal{N}}_{\overline{\overline{{I}_{j}}}}}}}^{L} \left( \widetilde {{x}_{E}} \right) ,{\overline{\overline{{\mathcal{N}}_{\overline{\overline{{I}_{j}}}}}}}^{U} \left( \widetilde {{x}_{E}} \right) \right] \right) } \end{array}} \right) $, if ${\overline{\overline{\mathfrak{C}}}}^{-}= \left( \left[ {\min }_{j}{\overline{\overline{{\mathcal{M}}_{\overline{\overline{{R}_{j}}}}}}}^{L}~,{\min }_{j}{\overline{\overline{{\mathcal{M}}_{\overline{\overline{{R}_{j}}}}}}}^{U}~ \right] {e}^{i2\pi \left[ {\min }_{j}{\overline{\overline{{\mathcal{M}}_{\overline{\overline{{I}_{j}}}}}}}^{L}~,{\min }_{j}{\overline{\overline{{\mathcal{M}}_{\overline{\overline{{I}_{j}}}}}}}^{U}~ \right] } \right. $, $ \left. \left[ {\max }_{j}{\overline{\overline{{\mathcal{N}}_{\overline{\overline{{R}_{j}}}}}}}^{L}~,{\max }_{j}{\overline{\overline{{\mathcal{N}}_{\overline{\overline{{R}_{j}}}}}}}^{U}~ \right] {e}^{i2\pi \left[ {\max }_{j}{\overline{\overline{{\mathcal{N}}_{\overline{\overline{{I}_{j}}}}}}}^{L}~,{\max }_{j}{\overline{\overline{{\mathcal{N}}_{\overline{\overline{{I}_{j}}}}}}}^{U}~ \right] } \right) $ and

${\overline{\overline{\mathfrak{C}}}}^{+}= \left( \left[ {\max }_{j}{\overline{\overline{{\mathcal{M}}_{\overline{\overline{{R}_{j}}}}}}}^{L}~,{\max }_{j}{\overline{\overline{{\mathcal{M}}_{\overline{\overline{{R}_{j}}}}}}}^{U}~ \right] {e}^{i2\pi \left[ {\max }_{j}{\overline{\overline{{\mathcal{M}}_{\overline{\overline{{I}_{j}}}}}}}^{L}~,{\max }_{j}{\overline{\overline{{\mathcal{M}}_{\overline{\overline{{I}_{j}}}}}}}^{U}~ \right] } \right. $, $ \left. \left[ {\min }_{j}{\overline{\overline{{\mathcal{N}}_{\overline{\overline{{R}_{j}}}}}}}^{L}~,{\min }_{j}{\overline{\overline{{\mathcal{N}}_{\overline{\overline{{R}_{j}}}}}}}^{U}~ \right] {e}^{i2\pi \left[ {\min }_{j}{\overline{\overline{{\mathcal{N}}_{\overline{\overline{{I}_{j}}}}}}}^{L}~,{\min }_{j}{\overline{\overline{{\mathcal{N}}_{\overline{\overline{{I}_{j}}}}}}}^{U}~ \right] } \right) $, then

(33) \begin{eqnarray*}{\overline{\overline{\mathfrak{C}}}}^{-}\leq CIVIFAAHA \left( \overline{\overline{{\mathfrak{C}}_{{C}_{1}}}},\overline{\overline{{\mathfrak{C}}_{{C}_{2}}}},\ldots ,\overline{\overline{{\mathfrak{C}}_{{C}_{n}}}} \right) \leq {\overline{\overline{\mathfrak{C}}}}^{+}\end{eqnarray*}

**Boundedness-Property 9:** By taking CIVIFNs

$\overline{\overline{{\mathfrak{C}}_{{C}_{j}}}}= \left( {\scriptsize \begin{array}{@{}c@{}} \displaystyle \left[ {\overline{\overline{{\mathcal{M}}_{\overline{\overline{{R}_{j}}}}}}}^{L} \left( \widetilde {{x}_{E}} \right) ,{\overline{\overline{{\mathcal{M}}_{\overline{\overline{{R}_{j}}}}}}}^{U} \left( \widetilde {{x}_{E}} \right) \right] {e}^{i2\pi \left( \left[ {\overline{\overline{{\mathcal{M}}_{\overline{\overline{{I}_{j}}}}}}}^{L} \left( \widetilde {{x}_{E}} \right) ,{\overline{\overline{{\mathcal{M}}_{\overline{\overline{{I}_{j}}}}}}}^{U} \left( \widetilde {{x}_{E}} \right) \right] \right) },\\ \displaystyle \left[ {\overline{\overline{{\mathcal{N}}_{\overline{\overline{{R}_{j}}}}}}}^{L} \left( \widetilde {{x}_{E}} \right) ,{\overline{\overline{{\mathcal{N}}_{\overline{\overline{{R}_{j}}}}}}}^{U} \left( \widetilde {{x}_{E}} \right) \right] {e}^{i2\pi \left( \left[ {\overline{\overline{{\mathcal{N}}_{\overline{\overline{{I}_{j}}}}}}}^{L} \left( \widetilde {{x}_{E}} \right) ,{\overline{\overline{{\mathcal{N}}_{\overline{\overline{{I}_{j}}}}}}}^{U} \left( \widetilde {{x}_{E}} \right) \right] \right) } \end{array}} \right) ,j=1,2,\ldots ,n$, if $\overline{\overline{{\mathfrak{C}}_{{C}_{j}}}}\leq {\overline{\overline{{\mathfrak{C}}_{{C}_{j}}}}}^{{}^{{^{\prime}}}}$, then

(34) \begin{eqnarray*}CIVIFAAHA \left( \overline{\overline{{\mathfrak{C}}_{{C}_{1}}}},\overline{\overline{{\mathfrak{C}}_{{C}_{2}}}},\ldots ,\overline{\overline{{\mathfrak{C}}_{{C}_{n}}}} \right) \leq CIVIFAAHA \left( {\overline{\overline{{\mathfrak{C}}_{{C}_{1}}}}}^{{}^{{^{\prime}}}},{\overline{\overline{{\mathfrak{C}}_{{C}_{2}}}}}^{{}^{{^{\prime}}}},\ldots ,{\overline{\overline{{\mathfrak{C}}_{{C}_{n}}}}}^{{}^{{^{\prime}}}} \right) \end{eqnarray*}

**Definition 14:** By taking CIVIFNs $\overline{\overline{{\mathfrak{C}}_{{C}_{j}}}}= \left( {\scriptsize \begin{array}{@{}c@{}} \displaystyle \left[ {\overline{\overline{{\mathcal{M}}_{\overline{\overline{{R}_{j}}}}}}}^{L} \left( \widetilde {{x}_{E}} \right) ,{\overline{\overline{{\mathcal{M}}_{\overline{\overline{{R}_{j}}}}}}}^{U} \left( \widetilde {{x}_{E}} \right) \right] {e}^{i2\pi \left( \left[ {\overline{\overline{{\mathcal{M}}_{\overline{\overline{{I}_{j}}}}}}}^{L} \left( \widetilde {{x}_{E}} \right) ,{\overline{\overline{{\mathcal{M}}_{\overline{\overline{{I}_{j}}}}}}}^{U} \left( \widetilde {{x}_{E}} \right) \right] \right) },\\ \displaystyle \left[ {\overline{\overline{{\mathcal{N}}_{\overline{\overline{{R}_{j}}}}}}}^{L} \left( \widetilde {{x}_{E}} \right) ,{\overline{\overline{{\mathcal{N}}_{\overline{\overline{{R}_{j}}}}}}}^{U} \left( \widetilde {{x}_{E}} \right) \right] {e}^{i2\pi \left( \left[ {\overline{\overline{{\mathcal{N}}_{\overline{\overline{{I}_{j}}}}}}}^{L} \left( \widetilde {{x}_{E}} \right) ,{\overline{\overline{{\mathcal{N}}_{\overline{\overline{{I}_{j}}}}}}}^{U} \left( \widetilde {{x}_{E}} \right) \right] \right) } \end{array}} \right) $, then the CIVIFAAWG operator is interpreted as:

(35) \begin{eqnarray*}CIVIFAAWG \left( \overline{\overline{{\mathfrak{C}}_{{C}_{1}}}},\overline{\overline{{\mathfrak{C}}_{{C}_{2}}}},\ldots ,\overline{\overline{{\mathfrak{C}}_{{C}_{n}}}} \right) ={\overline{\overline{{\mathfrak{C}}_{{C}_{1}}}}}^{\overline{\overline{{\mathfrak{W}}_{1}}}}\otimes {\overline{\overline{{\mathfrak{C}}_{{C}_{2}}}}}^{\overline{\overline{{\mathfrak{W}}_{2}}}}\otimes \cdots \otimes {\overline{\overline{{\mathfrak{C}}_{{C}_{n}}}}}^{\overline{\overline{{\mathfrak{W}}_{n}}}}={\mathop{\otimes \nolimits }\nolimits }_{j=1}^{n} \left( {\overline{\overline{{\mathfrak{C}}_{{C}_{j}}}}}^{\overline{\overline{{\mathfrak{W}}_{j}}}} \right) \end{eqnarray*}

**Theorem 4:** By taking CIVIFNs $\overline{\overline{{\mathfrak{C}}_{{C}_{j}}}}= \left( {\scriptsize \begin{array}{@{}c@{}} \displaystyle \left[ {\overline{\overline{{\mathcal{M}}_{\overline{\overline{{R}_{j}}}}}}}^{L} \left( \widetilde {{x}_{E}} \right) ,{\overline{\overline{{\mathcal{M}}_{\overline{\overline{{R}_{j}}}}}}}^{U} \left( \widetilde {{x}_{E}} \right) \right] {e}^{i2\pi \left( \left[ {\overline{\overline{{\mathcal{M}}_{\overline{\overline{{I}_{j}}}}}}}^{L} \left( \widetilde {{x}_{E}} \right) ,{\overline{\overline{{\mathcal{M}}_{\overline{\overline{{I}_{j}}}}}}}^{U} \left( \widetilde {{x}_{E}} \right) \right] \right) },\\ \displaystyle \left[ {\overline{\overline{{\mathcal{N}}_{\overline{\overline{{R}_{j}}}}}}}^{L} \left( \widetilde {{x}_{E}} \right) ,{\overline{\overline{{\mathcal{N}}_{\overline{\overline{{R}_{j}}}}}}}^{U} \left( \widetilde {{x}_{E}} \right) \right] {e}^{i2\pi \left( \left[ {\overline{\overline{{\mathcal{N}}_{\overline{\overline{{I}_{j}}}}}}}^{L} \left( \widetilde {{x}_{E}} \right) ,{\overline{\overline{{\mathcal{N}}_{\overline{\overline{{I}_{j}}}}}}}^{U} \left( \widetilde {{x}_{E}} \right) \right] \right) } \end{array}} \right) $, then by using Eq. (35), we elaborate

(36) \begin{eqnarray*}CIVIFAAWG \left( \overline{\overline{{\mathfrak{C}}_{{C}_{1}}}},\overline{\overline{{\mathfrak{C}}_{{C}_{2}}}},\ldots ,\overline{\overline{{\mathfrak{C}}_{{C}_{n}}}} \right) \nonumber\\\displaystyle = \left( \begin{array}{@{}c@{}} \displaystyle \left[ \left( {e}^{-{ \left( \sum _{j=1}^{n}{ \left( -\text{log} \left( {\overline{\overline{{\mathcal{M}}_{\overline{\overline{{R}_{j}}}}}}}^{L} \right) ~ \right) }^{\psi \overline{\overline{{\mathfrak{W}}_{j}}}} \right) }^{ \frac{1}{\psi } }} \right) , \left( {e}^{-{ \left( \sum _{j=1}^{n}{ \left( -\text{log} \left( {\overline{\overline{{\mathcal{M}}_{\overline{\overline{{R}_{j}}}}}}}^{U} \right) ~ \right) }^{\psi \overline{\overline{{\mathfrak{W}}_{j}}}} \right) }^{ \frac{1}{\psi } }} \right) \right] \\ \displaystyle {e}^{i2\pi \left[ \left( {e}^{-{ \left( \sum _{j=1}^{n}{ \left( -\text{log} \left( {\overline{\overline{{\mathcal{M}}_{\overline{\overline{{I}_{j}}}}}}}^{L} \right) ~ \right) }^{\psi \overline{\overline{{\mathfrak{W}}_{j}}}} \right) }^{ \frac{1}{\psi } }} \right) , \left( {e}^{-{ \left( \sum _{j=1}^{n}{ \left( -\text{log} \left( {\overline{\overline{{\mathcal{M}}_{\overline{\overline{{I}_{j}}}}}}}^{U} \right) ~ \right) }^{\psi \overline{\overline{{\mathfrak{W}}_{j}}}} \right) }^{ \frac{1}{\psi } }} \right) \right] },\\ \displaystyle \left[ \left( 1-{e}^{-{ \left( \sum _{j=1}^{n}{ \left( -\text{log} \left( 1-{\overline{\overline{{\mathcal{N}}_{\overline{\overline{{R}_{j}}}}}}}^{L} \right) ~ \right) }^{\psi \overline{\overline{{\mathfrak{W}}_{j}}}} \right) }^{ \frac{1}{\psi } }} \right) , \left( 1-{e}^{-{ \left( \sum _{j=1}^{n}{ \left( -\text{log} \left( 1-{\overline{\overline{{\mathcal{N}}_{\overline{\overline{{R}_{j}}}}}}}^{U} \right) ~ \right) }^{\psi \overline{\overline{{\mathfrak{W}}_{j}}}} \right) }^{ \frac{1}{\psi } }} \right) \right] \\ \displaystyle {e}^{i2\pi \left[ \left( 1-{e}^{-{ \left( \sum _{j=1}^{n}{ \left( -\text{log} \left( 1-{\overline{\overline{{\mathcal{N}}_{\overline{\overline{{I}_{j}}}}}}}^{L} \right) ~ \right) }^{\psi \overline{\overline{{\mathfrak{W}}_{j}}}} \right) }^{ \frac{1}{\psi } }} \right) , \left( 1-{e}^{-{ \left( \sum _{j=1}^{n}{ \left( -\text{log} \left( 1-{\overline{\overline{{\mathcal{N}}_{\overline{\overline{{I}_{j}}}}}}}^{U} \right) ~ \right) }^{\psi \overline{\overline{{\mathfrak{W}}_{j}}}} \right) }^{ \frac{1}{\psi } }} \right) \right] } \end{array} \right) \end{eqnarray*}

**Definition 15:** For CIVIFNs $\overline{\overline{{\mathfrak{C}}_{{C}_{j}}}}= \left( {\scriptsize \begin{array}{@{}c@{}} \displaystyle \left[ {\overline{\overline{{\mathcal{M}}_{\overline{\overline{{R}_{j}}}}}}}^{L} \left( \widetilde {{x}_{E}} \right) ,{\overline{\overline{{\mathcal{M}}_{\overline{\overline{{R}_{j}}}}}}}^{U} \left( \widetilde {{x}_{E}} \right) \right] {e}^{i2\pi \left( \left[ {\overline{\overline{{\mathcal{M}}_{\overline{\overline{{I}_{j}}}}}}}^{L} \left( \widetilde {{x}_{E}} \right) ,{\overline{\overline{{\mathcal{M}}_{\overline{\overline{{I}_{j}}}}}}}^{U} \left( \widetilde {{x}_{E}} \right) \right] \right) },\\ \displaystyle \left[ {\overline{\overline{{\mathcal{N}}_{\overline{\overline{{R}_{j}}}}}}}^{L} \left( \widetilde {{x}_{E}} \right) ,{\overline{\overline{{\mathcal{N}}_{\overline{\overline{{R}_{j}}}}}}}^{U} \left( \widetilde {{x}_{E}} \right) \right] {e}^{i2\pi \left( \left[ {\overline{\overline{{\mathcal{N}}_{\overline{\overline{{I}_{j}}}}}}}^{L} \left( \widetilde {{x}_{E}} \right) ,{\overline{\overline{{\mathcal{N}}_{\overline{\overline{{I}_{j}}}}}}}^{U} \left( \widetilde {{x}_{E}} \right) \right] \right) } \end{array}} \right) ,j=1,\ldots ,n$, then the CIVIFAAOWG operator is interpreted as:

(37) \begin{eqnarray*}CIVIFAAOWG \left( \overline{\overline{{\mathfrak{C}}_{{C}_{1}}}},\overline{\overline{{\mathfrak{C}}_{{C}_{2}}}},\ldots ,\overline{\overline{{\mathfrak{C}}_{{C}_{n}}}} \right) \nonumber\\\displaystyle ={\overline{\overline{{\mathfrak{C}}_{{C}_{\varphi \left( 1 \right) }}}}}^{\overline{\overline{{\mathfrak{W}}_{1}}}}\otimes {\overline{\overline{{\mathfrak{C}}_{{C}_{\varphi \left( 2 \right) }}}}}^{\overline{\overline{{\mathfrak{W}}_{2}}}}\otimes \cdots \otimes {\overline{{\mathfrak{C}}_{{C}_{\varphi \left( n \right) }}}}^{\overline{\overline{{\mathfrak{W}}_{n}}}}={\mathop{\otimes \nolimits }\nolimits }_{j=1}^{n} \left( {\overline{\overline{{\mathfrak{C}}_{{C}_{\varphi \left( j \right) }}}}}^{\overline{\overline{{\mathfrak{W}}_{j}}}} \right) \end{eqnarray*}

where $\overline{\overline{\mathfrak{W}}}={ \left( {\overline{\overline{\mathfrak{W}}}}_{1},{\overline{\overline{\mathfrak{W}}}}_{2},\ldots ,{\overline{\overline{\mathfrak{W}}}}_{n} \right) }^{T}$ indicates the weight of *C*_*j*_, with a rule ${\mathop{\sum }\nolimits }_{j=1}^{n}{\overline{\overline{\mathfrak{W}}}}_{j}=1$, with parameter $\varphi \left( 1 \right) ,\varphi \left( 2 \right) ,\ldots ,\varphi \left( n \right) $ based on $\overline{\overline{{\mathfrak{C}}_{{C}_{\varphi \left( j \right) }}}}\leq \overline{\overline{{\mathfrak{C}}_{{C}_{\varphi \left( j-1 \right) }}}}$.

**Theorem 5:** For CIVIFNs $\overline{\overline{{\mathfrak{C}}_{{C}_{j}}}}= \left( {\scriptsize \begin{array}{@{}c@{}} \displaystyle \left[ {\overline{\overline{{\mathcal{M}}_{\overline{\overline{{R}_{j}}}}}}}^{L} \left( \widetilde {{x}_{E}} \right) ,{\overline{\overline{{\mathcal{M}}_{\overline{\overline{{R}_{j}}}}}}}^{U} \left( \widetilde {{x}_{E}} \right) \right] {e}^{i2\pi \left( \left[ {\overline{\overline{{\mathcal{M}}_{\overline{\overline{{I}_{j}}}}}}}^{L} \left( \widetilde {{x}_{E}} \right) ,{\overline{\overline{{\mathcal{M}}_{\overline{\overline{{I}_{j}}}}}}}^{U} \left( \widetilde {{x}_{E}} \right) \right] \right) },\\ \displaystyle \left[ {\overline{\overline{{\mathcal{N}}_{\overline{\overline{{R}_{j}}}}}}}^{L} \left( \widetilde {{x}_{E}} \right) ,{\overline{\overline{{\mathcal{N}}_{\overline{\overline{{R}_{j}}}}}}}^{U} \left( \widetilde {{x}_{E}} \right) \right] {e}^{i2\pi \left( \left[ {\overline{\overline{{\mathcal{N}}_{\overline{\overline{{I}_{j}}}}}}}^{L} \left( \widetilde {{x}_{E}} \right) ,{\overline{\overline{{\mathcal{N}}_{\overline{\overline{{I}_{j}}}}}}}^{U} \left( \widetilde {{x}_{E}} \right) \right] \right) } \end{array}} \right) ,j=1,\ldots ,n$, then we elaborate

(38) \begin{eqnarray*}CIVIFAAOWG \left( \overline{\overline{{\mathfrak{C}}_{{C}_{1}}}},\overline{\overline{{\mathfrak{C}}_{{C}_{2}}}},\ldots ,\overline{\overline{{\mathfrak{C}}_{{C}_{n}}}} \right) \nonumber\\\displaystyle = \left( \begin{array}{@{}c@{}} \displaystyle \left[ \left( {e}^{-{ \left( \sum _{j=1}^{n}{ \left( -\text{log} \left( {\overline{{\mathcal{M}}_{\overline{{R}_{\varphi \left( j \right) }}}}}^{L} \right) ~ \right) }^{\psi \overline{\overline{{\mathfrak{W}}_{j}}}} \right) }^{ \frac{1}{\psi } }} \right) , \left( {e}^{-{ \left( \sum _{j=1}^{n}{ \left( -\text{log} \left( {\overline{{\mathcal{M}}_{\overline{{R}_{\varphi \left( j \right) }}}}}^{U} \right) ~ \right) }^{\psi \overline{\overline{{\mathfrak{W}}_{j}}}} \right) }^{ \frac{1}{\psi } }} \right) \right] \\ \displaystyle {e}^{i2\pi \left[ \left( {e}^{-{ \left( \sum _{j=1}^{n}{ \left( -\text{log} \left( {\overline{{\mathcal{M}}_{\overline{{I}_{\varphi \left( j \right) }}}}}^{L} \right) ~ \right) }^{\psi \overline{\overline{{\mathfrak{W}}_{j}}}} \right) }^{ \frac{1}{\psi } }} \right) , \left( {e}^{-{ \left( \sum _{j=1}^{n}{ \left( -\text{log} \left( {\overline{{\mathcal{M}}_{\overline{{I}_{\varphi \left( j \right) }}}}}^{U} \right) ~ \right) }^{\psi \overline{\overline{{\mathfrak{W}}_{j}}}} \right) }^{ \frac{1}{\psi } }} \right) \right] },\\ \displaystyle \left[ \left( 1-{e}^{-{ \left( \sum _{j=1}^{n}{ \left( -\text{log} \left( 1-{\overline{{\mathcal{N}}_{\overline{{R}_{\varphi \left( j \right) }}}}}^{L} \right) ~ \right) }^{\psi \overline{\overline{{\mathfrak{W}}_{j}}}} \right) }^{ \frac{1}{\psi } }} \right) , \left( 1-{e}^{-{ \left( \sum _{j=1}^{n}{ \left( -\text{log} \left( 1-{\overline{{\mathcal{N}}_{\overline{{R}_{\varphi \left( j \right) }}}}}^{U} \right) ~ \right) }^{\psi \overline{\overline{{\mathfrak{W}}_{j}}}} \right) }^{ \frac{1}{\psi } }} \right) \right] \\ \displaystyle {e}^{i2\pi \left[ \left( 1-{e}^{-{ \left( \sum _{j=1}^{n}{ \left( -\text{log} \left( 1-{\overline{{\mathcal{N}}_{\overline{{I}_{\varphi \left( j \right) }}}}}^{L} \right) ~ \right) }^{\psi \overline{\overline{{\mathfrak{W}}_{j}}}} \right) }^{ \frac{1}{\psi } }} \right) , \left( 1-{e}^{-{ \left( \sum _{j=1}^{n}{ \left( -\text{log} \left( 1-{\overline{{\mathcal{N}}_{\overline{{I}_{\varphi \left( j \right) }}}}}^{U} \right) ~ \right) }^{\psi \overline{\overline{{\mathfrak{W}}_{j}}}} \right) }^{ \frac{1}{\psi } }} \right) \right] } \end{array} \right) \end{eqnarray*}

**Idempotency-Property 10:** By taking CIVIFNs

$\overline{\overline{{\mathfrak{C}}_{{C}_{j}}}}= \left( {\scriptsize \begin{array}{@{}c@{}} \displaystyle \left[ {\overline{\overline{{\mathcal{M}}_{\overline{\overline{{R}_{j}}}}}}}^{L} \left( \widetilde {{x}_{E}} \right) ,{\overline{\overline{{\mathcal{M}}_{\overline{\overline{{R}_{j}}}}}}}^{U} \left( \widetilde {{x}_{E}} \right) \right] {e}^{i2\pi \left( \left[ {\overline{\overline{{\mathcal{M}}_{\overline{\overline{{I}_{j}}}}}}}^{L} \left( \widetilde {{x}_{E}} \right) ,{\overline{\overline{{\mathcal{M}}_{\overline{\overline{{I}_{j}}}}}}}^{U} \left( \widetilde {{x}_{E}} \right) \right] \right) },\\ \displaystyle \left[ {\overline{\overline{{\mathcal{N}}_{\overline{\overline{{R}_{j}}}}}}}^{L} \left( \widetilde {{x}_{E}} \right) ,{\overline{\overline{{\mathcal{N}}_{\overline{\overline{{R}_{j}}}}}}}^{U} \left( \widetilde {{x}_{E}} \right) \right] {e}^{i2\pi \left( \left[ {\overline{\overline{{\mathcal{N}}_{\overline{\overline{{I}_{j}}}}}}}^{L} \left( \widetilde {{x}_{E}} \right) ,{\overline{\overline{{\mathcal{N}}_{\overline{\overline{{I}_{j}}}}}}}^{U} \left( \widetilde {{x}_{E}} \right) \right] \right) } \end{array}} \right) ,j=1,2,\ldots ,n$, if $\overline{\overline{{\mathfrak{C}}_{{C}_{j}}}}=\overline{\overline{\mathfrak{C}}}$, then

(39) \begin{eqnarray*}CIVIFAAOWG \left( \overline{\overline{{\mathfrak{C}}_{{C}_{1}}}},\overline{\overline{{\mathfrak{C}}_{{C}_{2}}}},\ldots ,\overline{\overline{{\mathfrak{C}}_{{C}_{n}}}} \right) =\overline{\overline{\mathfrak{C}}}\end{eqnarray*}

**Monotonicity-Property 11:** By taking CIVIFNs $\overline{\overline{{\mathfrak{C}}_{{C}_{j}}}}= \left( {\scriptsize \begin{array}{@{}c@{}} \displaystyle \left[ {\overline{\overline{{\mathcal{M}}_{\overline{\overline{{R}_{j}}}}}}}^{L} \left( \widetilde {{x}_{E}} \right) ,{\overline{\overline{{\mathcal{M}}_{\overline{\overline{{R}_{j}}}}}}}^{U} \left( \widetilde {{x}_{E}} \right) \right] {e}^{i2\pi \left( \left[ {\overline{\overline{{\mathcal{M}}_{\overline{\overline{{I}_{j}}}}}}}^{L} \left( \widetilde {{x}_{E}} \right) ,{\overline{\overline{{\mathcal{M}}_{\overline{\overline{{I}_{j}}}}}}}^{U} \left( \widetilde {{x}_{E}} \right) \right] \right) },\\ \displaystyle \left[ {\overline{\overline{{\mathcal{N}}_{\overline{\overline{{R}_{j}}}}}}}^{L} \left( \widetilde {{x}_{E}} \right) ,{\overline{\overline{{\mathcal{N}}_{\overline{\overline{{R}_{j}}}}}}}^{U} \left( \widetilde {{x}_{E}} \right) \right] {e}^{i2\pi \left( \left[ {\overline{\overline{{\mathcal{N}}_{\overline{\overline{{I}_{j}}}}}}}^{L} \left( \widetilde {{x}_{E}} \right) ,{\overline{\overline{{\mathcal{N}}_{\overline{\overline{{I}_{j}}}}}}}^{U} \left( \widetilde {{x}_{E}} \right) \right] \right) } \end{array}} \right) ,j=1,2,\ldots ,n$, if

${\overline{\overline{\mathfrak{C}}}}^{-}= \left( \left[ {\min }_{j}{\overline{\overline{{\mathcal{M}}_{\overline{\overline{{R}_{j}}}}}}}^{L}~,{\min }_{j}{\overline{\overline{{\mathcal{M}}_{\overline{\overline{{R}_{j}}}}}}}^{U}~ \right] {e}^{i2\pi \left[ {\min }_{j}{\overline{\overline{{\mathcal{M}}_{\overline{\overline{{I}_{j}}}}}}}^{L}~,{\min }_{j}{\overline{\overline{{\mathcal{M}}_{\overline{\overline{{I}_{j}}}}}}}^{U}~ \right] }, \left[ {\max }_{j}{\overline{\overline{{\mathcal{N}}_{\overline{\overline{{R}_{j}}}}}}}^{L}~,{\max }_{j}{\overline{\overline{{\mathcal{N}}_{\overline{\overline{{R}_{j}}}}}}}^{U}~ \right] \right. $

$ \left. {e}^{i2\pi \left[ {\max }_{j}{\overline{\overline{{\mathcal{N}}_{\overline{\overline{{I}_{j}}}}}}}^{L}~,{\max }_{j}{\overline{\overline{{\mathcal{N}}_{\overline{\overline{{I}_{j}}}}}}}^{U}~ \right] } \right) $ and ${\overline{\overline{\mathfrak{C}}}}^{+}= \left( \left[ {\max }_{j}{\overline{\overline{{\mathcal{M}}_{\overline{\overline{{R}_{j}}}}}}}^{L}~,{\max }_{j}{\overline{\overline{{\mathcal{M}}_{\overline{\overline{{R}_{j}}}}}}}^{U}~ \right] \right. $

$ \left. {e}^{i2\pi \left[ {\max }_{j}{\overline{\overline{{\mathcal{M}}_{\overline{\overline{{I}_{j}}}}}}}^{L}~,{\max }_{j}{\overline{\overline{{\mathcal{M}}_{\overline{\overline{{I}_{j}}}}}}}^{U}~ \right] }, \left[ {\min }_{j}{\overline{\overline{{\mathcal{N}}_{\overline{\overline{{R}_{j}}}}}}}^{L}~,{\min }_{j}{\overline{\overline{{\mathcal{N}}_{\overline{\overline{{R}_{j}}}}}}}^{U}~ \right] {e}^{i2\pi \left[ {\min }_{j}{\overline{\overline{{\mathcal{N}}_{\overline{\overline{{I}_{j}}}}}}}^{L}~,{\min }_{j}{\overline{\overline{{\mathcal{N}}_{\overline{\overline{{I}_{j}}}}}}}^{U}~ \right] } \right) $, then

(40) \begin{eqnarray*}{\overline{\overline{\mathfrak{C}}}}^{-}\leq CIVIFAAOWG \left( \overline{\overline{{\mathfrak{C}}_{{C}_{1}}}},\overline{\overline{{\mathfrak{C}}_{{C}_{2}}}},\ldots ,\overline{\overline{{\mathfrak{C}}_{{C}_{n}}}} \right) \leq {\overline{\overline{\mathfrak{C}}}}^{+}\end{eqnarray*}

**Boundedness-Property 12:** By taking CIVIFNs

$\overline{\overline{{\mathfrak{C}}_{{C}_{j}}}}= \left( {\scriptsize \begin{array}{@{}c@{}} \displaystyle \left[ {\overline{\overline{{\mathcal{M}}_{\overline{\overline{{R}_{j}}}}}}}^{L} \left( \widetilde {{x}_{E}} \right) ,{\overline{\overline{{\mathcal{M}}_{\overline{\overline{{R}_{j}}}}}}}^{U} \left( \widetilde {{x}_{E}} \right) \right] {e}^{i2\pi \left( \left[ {\overline{\overline{{\mathcal{M}}_{\overline{\overline{{I}_{j}}}}}}}^{L} \left( \widetilde {{x}_{E}} \right) ,{\overline{\overline{{\mathcal{M}}_{\overline{\overline{{I}_{j}}}}}}}^{U} \left( \widetilde {{x}_{E}} \right) \right] \right) },\\ \displaystyle \left[ {\overline{\overline{{\mathcal{N}}_{\overline{\overline{{R}_{j}}}}}}}^{L} \left( \widetilde {{x}_{E}} \right) ,{\overline{\overline{{\mathcal{N}}_{\overline{\overline{{R}_{j}}}}}}}^{U} \left( \widetilde {{x}_{E}} \right) \right] {e}^{i2\pi \left( \left[ {\overline{\overline{{\mathcal{N}}_{\overline{\overline{{I}_{j}}}}}}}^{L} \left( \widetilde {{x}_{E}} \right) ,{\overline{\overline{{\mathcal{N}}_{\overline{\overline{{I}_{j}}}}}}}^{U} \left( \widetilde {{x}_{E}} \right) \right] \right) } \end{array}} \right) ,j=1,2,\ldots ,n$, if $\overline{\overline{{\mathfrak{C}}_{{C}_{j}}}}\leq {\overline{\overline{{\mathfrak{C}}_{{C}_{j}}}}}^{{}^{{^{\prime}}}}$, then

(41) \begin{eqnarray*}CIVIFAAOWG \left( \overline{\overline{{\mathfrak{C}}_{{C}_{1}}}},\overline{\overline{{\mathfrak{C}}_{{C}_{2}}}},\ldots ,\overline{\overline{{\mathfrak{C}}_{{C}_{n}}}} \right) \leq CIVIFAAOWG \left( {\overline{\overline{{\mathfrak{C}}_{{C}_{1}}}}}^{{}^{{^{\prime}}}},{\overline{\overline{{\mathfrak{C}}_{{C}_{2}}}}}^{{}^{{^{\prime}}}},\ldots ,{\overline{\overline{{\mathfrak{C}}_{{C}_{n}}}}}^{{}^{{^{\prime}}}} \right) \end{eqnarray*}

**Definition 16:** For CIVIFNs $\overline{\overline{{\mathfrak{C}}_{{C}_{j}}}}= \left( {\scriptsize \begin{array}{@{}c@{}} \displaystyle \left[ {\overline{\overline{{\mathcal{M}}_{\overline{\overline{{R}_{j}}}}}}}^{L} \left( \widetilde {{x}_{E}} \right) ,{\overline{\overline{{\mathcal{M}}_{\overline{\overline{{R}_{j}}}}}}}^{U} \left( \widetilde {{x}_{E}} \right) \right] {e}^{i2\pi \left( \left[ {\overline{\overline{{\mathcal{M}}_{\overline{\overline{{I}_{j}}}}}}}^{L} \left( \widetilde {{x}_{E}} \right) ,{\overline{\overline{{\mathcal{M}}_{\overline{\overline{{I}_{j}}}}}}}^{U} \left( \widetilde {{x}_{E}} \right) \right] \right) },\\ \displaystyle \left[ {\overline{\overline{{\mathcal{N}}_{\overline{\overline{{R}_{j}}}}}}}^{L} \left( \widetilde {{x}_{E}} \right) ,{\overline{\overline{{\mathcal{N}}_{\overline{\overline{{R}_{j}}}}}}}^{U} \left( \widetilde {{x}_{E}} \right) \right] {e}^{i2\pi \left( \left[ {\overline{\overline{{\mathcal{N}}_{\overline{\overline{{I}_{j}}}}}}}^{L} \left( \widetilde {{x}_{E}} \right) ,{\overline{\overline{{\mathcal{N}}_{\overline{\overline{{I}_{j}}}}}}}^{U} \left( \widetilde {{x}_{E}} \right) \right] \right) } \end{array}} \right) ,j=1,\ldots ,n$, then the CIVIFAAHG operator is shown as:

(42) \begin{eqnarray*}CIVIFAAHG \left( \overline{\overline{{\mathfrak{C}}_{{C}_{1}}}},\overline{\overline{{\mathfrak{C}}_{{C}_{2}}}},\ldots ,\overline{\overline{{\mathfrak{C}}_{{C}_{n}}}} \right) \nonumber\\\displaystyle ={\overline{\overline{\dot {{\mathfrak{C}}_{{C}_{\varphi \left( 1 \right) }}}}}}^{\overline{\overline{{\mathfrak{W}}_{1}}}}\otimes {\overline{\overline{\dot {{\mathfrak{C}}_{{C}_{\varphi \left( 2 \right) }}}}}}^{\overline{\overline{{\mathfrak{W}}_{2}}}}\otimes \cdots \otimes {\overline{\overline{\dot {{\mathfrak{C}}_{{C}_{\varphi \left( n \right) }}}}}}^{\overline{\overline{{\mathfrak{W}}_{n}}}}={\mathop{\otimes \nolimits }\nolimits }_{j=1}^{n} \left( {\overline{\overline{\dot {{\mathfrak{C}}_{{C}_{\varphi \left( j \right) }}}}}}^{\overline{\overline{{\mathfrak{W}}_{j}}}} \right) \end{eqnarray*}

where $\overline{\overline{\mathfrak{W}}}={ \left( \overline{\overline{{\mathfrak{W}}_{1}}},\overline{\overline{{\mathfrak{W}}_{2}}},\ldots ,\overline{\overline{{\mathfrak{W}}_{n}}} \right) }^{T}$ indicates the weight of *C*_*j*_, with a rule ${\mathop{\sum }\nolimits }_{j=1}^{n}\overline{\overline{{\mathfrak{W}}_{j}}}=1$, with parameter $\varphi \left( 1 \right) ,\varphi \left( 2 \right) ,\ldots ,\varphi \left( n \right) $ based on $\overline{\overline{\dot {{\mathfrak{C}}_{{C}_{\varphi \left( j \right) }}}}}\leq \overline{\overline{\dot {{\mathfrak{C}}_{{C}_{\varphi \left( j-1 \right) }}}}}$. Additionally, $\overline{\overline{\dot {{\mathfrak{C}}_{{C}_{\varphi \left( j \right) }}}}}=n\overline{\overline{{\mathfrak{W}}_{j}^{{}^{{^{\prime}}{^{\prime}}}}}}\overline{\overline{\dot {{\mathfrak{C}}_{{C}_{\varphi \left( j \right) }}}}}$ with ${\mathop{\sum }\nolimits }_{j=1}^{n}\overline{\overline{{\mathfrak{W}}_{j}^{{}^{{^{\prime}}{^{\prime}}}}}}=1$.

**Theorem 6:** For CIVIFNs $\overline{\overline{{\mathfrak{C}}_{{C}_{j}}}}= \left( {\scriptsize \begin{array}{@{}c@{}} \displaystyle \left[ {\overline{\overline{{\mathcal{M}}_{\overline{\overline{{R}_{j}}}}}}}^{L} \left( \widetilde {{x}_{E}} \right) ,{\overline{\overline{{\mathcal{M}}_{\overline{\overline{{R}_{j}}}}}}}^{U} \left( \widetilde {{x}_{E}} \right) \right] {e}^{i2\pi \left( \left[ {\overline{\overline{{\mathcal{M}}_{\overline{\overline{{I}_{j}}}}}}}^{L} \left( \widetilde {{x}_{E}} \right) ,{\overline{\overline{{\mathcal{M}}_{\overline{\overline{{I}_{j}}}}}}}^{U} \left( \widetilde {{x}_{E}} \right) \right] \right) },\\ \displaystyle \left[ {\overline{\overline{{\mathcal{N}}_{\overline{\overline{{R}_{j}}}}}}}^{L} \left( \widetilde {{x}_{E}} \right) ,{\overline{\overline{{\mathcal{N}}_{\overline{\overline{{R}_{j}}}}}}}^{U} \left( \widetilde {{x}_{E}} \right) \right] {e}^{i2\pi \left( \left[ {\overline{\overline{{\mathcal{N}}_{\overline{\overline{{I}_{j}}}}}}}^{L} \left( \widetilde {{x}_{E}} \right) ,{\overline{\overline{{\mathcal{N}}_{\overline{\overline{{I}_{j}}}}}}}^{U} \left( \widetilde {{x}_{E}} \right) \right] \right) } \end{array}} \right) ,j=1,\ldots ,n$, then by using Eq. (42)), we elaborate

(43) \begin{eqnarray*}CIVIFAAHG \left( \overline{\overline{{\mathfrak{C}}_{{C}_{1}}}},\overline{\overline{{\mathfrak{C}}_{{C}_{2}}}},\ldots ,\overline{\overline{{\mathfrak{C}}_{{C}_{n}}}} \right) =\nonumber\\\displaystyle \left( \begin{array}{@{}c@{}} \displaystyle \left[ \left( {e}^{-{ \left( \sum _{j=1}^{n}{ \left( -\text{log} \left( {\overline{\overline{\dot {{\mathcal{M}}_{\overline{\overline{{R}_{\varphi \left( j \right) }}}}}}}}^{L} \right) ~ \right) }^{\psi \overline{\overline{{\mathfrak{W}}_{j}}}} \right) }^{ \frac{1}{\psi } }} \right) , \left( {e}^{-{ \left( \sum _{j=1}^{n}{ \left( -\text{log} \left( {\overline{\overline{\dot {{\mathcal{M}}_{\overline{\overline{{R}_{\varphi \left( j \right) }}}}}}}}^{U} \right) ~ \right) }^{\psi \overline{\overline{{\mathfrak{W}}_{j}}}} \right) }^{ \frac{1}{\psi } }} \right) \right] \\ \displaystyle {e}^{i2\pi \left[ \left( {e}^{-{ \left( \sum _{j=1}^{n}{ \left( -\text{log} \left( {\overline{\overline{\dot {{\mathcal{M}}_{\overline{\overline{{I}_{\varphi \left( j \right) }}}}}}}}^{L} \right) ~ \right) }^{\psi \overline{\overline{{\mathfrak{W}}_{j}}}} \right) }^{ \frac{1}{\psi } }} \right) , \left( {e}^{-{ \left( \sum _{j=1}^{n}{ \left( -\text{log} \left( {\overline{\overline{\dot {{\mathcal{M}}_{\overline{\overline{{I}_{\varphi \left( j \right) }}}}}}}}^{U} \right) ~ \right) }^{\psi \overline{\overline{{\mathfrak{W}}_{j}}}} \right) }^{ \frac{1}{\psi } }} \right) \right] },\\ \displaystyle \left[ \left( 1-{e}^{-{ \left( \sum _{j=1}^{n}{ \left( -\text{log} \left( 1-{\overline{\overline{\dot {{\mathcal{N}}_{\overline{\overline{{R}_{\varphi \left( j \right) }}}}}}}}^{L} \right) ~ \right) }^{\psi \overline{\overline{{\mathfrak{W}}_{j}}}} \right) }^{ \frac{1}{\psi } }} \right) , \left( 1-{e}^{-{ \left( \sum _{j=1}^{n}{ \left( -\text{log} \left( 1-{\overline{\overline{\dot {{\mathcal{N}}_{\overline{\overline{{R}_{\varphi \left( j \right) }}}}}}}}^{U} \right) ~ \right) }^{\psi \overline{\overline{{\mathfrak{W}}_{j}}}} \right) }^{ \frac{1}{\psi } }} \right) \right] \\ \displaystyle {e}^{i2\pi \left[ \left( 1-{e}^{-{ \left( \sum _{j=1}^{n}{ \left( -\text{log} \left( 1-{\overline{\overline{\dot {{\mathcal{N}}_{\overline{\overline{{I}_{\varphi \left( j \right) }}}}}}}}^{L} \right) ~ \right) }^{\psi \overline{\overline{{\mathfrak{W}}_{j}}}} \right) }^{ \frac{1}{\psi } }} \right) , \left( 1-{e}^{-{ \left( \sum _{j=1}^{n}{ \left( -\text{log} \left( 1-{\overline{\overline{\dot {{\mathcal{N}}_{\overline{\overline{{I}_{\varphi \left( j \right) }}}}}}}}^{U} \right) ~ \right) }^{\psi \overline{\overline{{\mathfrak{W}}_{j}}}} \right) }^{ \frac{1}{\psi } }} \right) \right] } \end{array} \right) \end{eqnarray*}

**Idempotency-Property 13:** By taking CIVIFNs

$\overline{\overline{{\mathfrak{C}}_{{C}_{j}}}}= \left( {\scriptsize \begin{array}{@{}c@{}} \displaystyle \left[ {\overline{\overline{{\mathcal{M}}_{\overline{\overline{{R}_{j}}}}}}}^{L} \left( \widetilde {{x}_{E}} \right) ,{\overline{\overline{{\mathcal{M}}_{\overline{\overline{{R}_{j}}}}}}}^{U} \left( \widetilde {{x}_{E}} \right) \right] {e}^{i2\pi \left( \left[ {\overline{\overline{{\mathcal{M}}_{\overline{\overline{{I}_{j}}}}}}}^{L} \left( \widetilde {{x}_{E}} \right) ,{\overline{\overline{{\mathcal{M}}_{\overline{\overline{{I}_{j}}}}}}}^{U} \left( \widetilde {{x}_{E}} \right) \right] \right) },\\ \displaystyle \left[ {\overline{\overline{{\mathcal{N}}_{\overline{\overline{{R}_{j}}}}}}}^{L} \left( \widetilde {{x}_{E}} \right) ,{\overline{\overline{{\mathcal{N}}_{\overline{\overline{{R}_{j}}}}}}}^{U} \left( \widetilde {{x}_{E}} \right) \right] {e}^{i2\pi \left( \left[ {\overline{\overline{{\mathcal{N}}_{\overline{\overline{{I}_{j}}}}}}}^{L} \left( \widetilde {{x}_{E}} \right) ,{\overline{\overline{{\mathcal{N}}_{\overline{\overline{{I}_{j}}}}}}}^{U} \left( \widetilde {{x}_{E}} \right) \right] \right) } \end{array}} \right) ,j=1,2,\ldots ,n$, if $\overline{\overline{{\mathfrak{C}}_{{C}_{j}}}}=\overline{\overline{\mathfrak{C}}}$, then

(44) \begin{eqnarray*}CIVIFAAHG \left( \overline{\overline{{\mathfrak{C}}_{{C}_{1}}}},\overline{\overline{{\mathfrak{C}}_{{C}_{2}}}},\ldots ,\overline{\overline{{\mathfrak{C}}_{{C}_{n}}}} \right) =\overline{\overline{\mathfrak{C}}}\end{eqnarray*}

**Monotonicity-Property 14:** By taking CIVIFNs

$\overline{\overline{{\mathfrak{C}}_{{C}_{j}}}}= \left( {\scriptsize \begin{array}{@{}c@{}} \displaystyle \left[ {\overline{\overline{{\mathcal{M}}_{\overline{\overline{{R}_{j}}}}}}}^{L} \left( \widetilde {{x}_{E}} \right) ,{\overline{\overline{{\mathcal{M}}_{\overline{\overline{{R}_{j}}}}}}}^{U} \left( \widetilde {{x}_{E}} \right) \right] {e}^{i2\pi \left( \left[ {\overline{\overline{{\mathcal{M}}_{\overline{\overline{{I}_{j}}}}}}}^{L} \left( \widetilde {{x}_{E}} \right) ,{\overline{\overline{{\mathcal{M}}_{\overline{\overline{{I}_{j}}}}}}}^{U} \left( \widetilde {{x}_{E}} \right) \right] \right) },\\ \displaystyle \left[ {\overline{\overline{{\mathcal{N}}_{\overline{\overline{{R}_{j}}}}}}}^{L} \left( \widetilde {{x}_{E}} \right) ,{\overline{\overline{{\mathcal{N}}_{\overline{\overline{{R}_{j}}}}}}}^{U} \left( \widetilde {{x}_{E}} \right) \right] {e}^{i2\pi \left( \left[ {\overline{\overline{{\mathcal{N}}_{\overline{\overline{{I}_{j}}}}}}}^{L} \left( \widetilde {{x}_{E}} \right) ,{\overline{\overline{{\mathcal{N}}_{\overline{\overline{{I}_{j}}}}}}}^{U} \left( \widetilde {{x}_{E}} \right) \right] \right) } \end{array}} \right) $ , *j* = 1, 2, …, *n*, if ${\overline{\overline{\mathfrak{C}}}}^{-}= \left( \left[ {\min }_{j}{\overline{\overline{{\mathcal{M}}_{\overline{\overline{{R}_{j}}}}}}}^{L}~,{\min }_{j}{\overline{\overline{{\mathcal{M}}_{\overline{\overline{{R}_{j}}}}}}}^{U}~ \right] {e}^{i2\pi \left[ {\min }_{j}{\overline{\overline{{\mathcal{M}}_{\overline{\overline{{I}_{j}}}}}}}^{L}~,{\min }_{j}{\overline{\overline{{\mathcal{M}}_{\overline{\overline{{I}_{j}}}}}}}^{U}~ \right] } \right. $,

$ \left. \left[ {\max }_{j}{\overline{\overline{{\mathcal{N}}_{\overline{\overline{{R}_{j}}}}}}}^{L}~,{\max }_{j}{\overline{\overline{{\mathcal{N}}_{\overline{\overline{{R}_{j}}}}}}}^{U}~ \right] {e}^{i2\pi \left[ {\max }_{j}{\overline{\overline{{\mathcal{N}}_{\overline{\overline{{I}_{j}}}}}}}^{L}~,{\max }_{j}{\overline{\overline{{\mathcal{N}}_{\overline{\overline{{I}_{j}}}}}}}^{U}~ \right] } \right) $ and

${\overline{\overline{\mathfrak{C}}}}^{+}= \left( \left[ {\max }_{j}{\overline{\overline{{\mathcal{M}}_{\overline{\overline{{R}_{j}}}}}}}^{L}~,{\max }_{j}{\overline{\overline{{\mathcal{M}}_{\overline{\overline{{R}_{j}}}}}}}^{U}~ \right] {e}^{i2\pi \left[ {\max }_{j}{\overline{\overline{{\mathcal{M}}_{\overline{\overline{{I}_{j}}}}}}}^{L}~,{\max }_{j}{\overline{\overline{{\mathcal{M}}_{\overline{\overline{{I}_{j}}}}}}}^{U}~ \right] } \right. $,

$ \left. \left[ {\min }_{j}{\overline{\overline{{\mathcal{N}}_{\overline{\overline{{R}_{j}}}}}}}^{L}~,{\min }_{j}{\overline{\overline{{\mathcal{N}}_{\overline{\overline{{R}_{j}}}}}}}^{U}~ \right] {e}^{i2\pi \left[ {\min }_{j}{\overline{\overline{{\mathcal{N}}_{\overline{\overline{{I}_{j}}}}}}}^{L}~,{\min }_{j}{\overline{\overline{{\mathcal{N}}_{\overline{\overline{{I}_{j}}}}}}}^{U}~ \right] } \right) $, then

(45) \begin{eqnarray*}{\overline{\overline{\mathfrak{C}}}}^{-}\leq CIVIFAAHG \left( \overline{\overline{{\mathfrak{C}}_{{C}_{1}}}},\overline{\overline{{\mathfrak{C}}_{{C}_{2}}}},\ldots ,\overline{\overline{{\mathfrak{C}}_{{C}_{n}}}} \right) \leq {\overline{\overline{\mathfrak{C}}}}^{+}\end{eqnarray*}

**Boundedness-Property 15:** By taking CIVIFNs

$\overline{\overline{{\mathfrak{C}}_{{C}_{j}}}}= \left( {\scriptsize \begin{array}{@{}c@{}} \displaystyle \left[ {\overline{\overline{{\mathcal{M}}_{\overline{\overline{{R}_{j}}}}}}}^{L} \left( \widetilde {{x}_{E}} \right) ,{\overline{\overline{{\mathcal{M}}_{\overline{\overline{{R}_{j}}}}}}}^{U} \left( \widetilde {{x}_{E}} \right) \right] {e}^{i2\pi \left( \left[ {\overline{\overline{{\mathcal{M}}_{\overline{\overline{{I}_{j}}}}}}}^{L} \left( \widetilde {{x}_{E}} \right) ,{\overline{\overline{{\mathcal{M}}_{\overline{\overline{{I}_{j}}}}}}}^{U} \left( \widetilde {{x}_{E}} \right) \right] \right) },\\ \displaystyle \left[ {\overline{\overline{{\mathcal{N}}_{\overline{\overline{{R}_{j}}}}}}}^{L} \left( \widetilde {{x}_{E}} \right) ,{\overline{\overline{{\mathcal{N}}_{\overline{\overline{{R}_{j}}}}}}}^{U} \left( \widetilde {{x}_{E}} \right) \right] {e}^{i2\pi \left( \left[ {\overline{\overline{{\mathcal{N}}_{\overline{\overline{{I}_{j}}}}}}}^{L} \left( \widetilde {{x}_{E}} \right) ,{\overline{\overline{{\mathcal{N}}_{\overline{\overline{{I}_{j}}}}}}}^{U} \left( \widetilde {{x}_{E}} \right) \right] \right) } \end{array}} \right) ,j=1,2,\ldots ,n$, if $\overline{\overline{{\mathfrak{C}}_{{C}_{j}}}}\leq {\overline{\overline{{\mathfrak{C}}_{{C}_{j}}}}}^{{}^{{^{\prime}}}}$, then

(46) \begin{eqnarray*}CIVIFAAHG \left( \overline{\overline{{\mathfrak{C}}_{{C}_{1}}}},\overline{\overline{{\mathfrak{C}}_{{C}_{2}}}},\ldots ,\overline{\overline{{\mathfrak{C}}_{{C}_{n}}}} \right) \leq CIVIFAAHG \left( {\overline{\overline{{\mathfrak{C}}_{{C}_{1}}}}}^{{}^{{^{\prime}}}},{\overline{\overline{{\mathfrak{C}}_{{C}_{2}}}}}^{{}^{{^{\prime}}}},\ldots ,{\overline{\overline{{\mathfrak{C}}_{{C}_{n}}}}}^{{}^{{^{\prime}}}} \right) \end{eqnarray*}

## WASPAS Method for CIVIFSs

The main theme of this section is to illustrate the WASPAS method for CIVIFSs and verify the validity of the proposed method with the help of some numerical examples.

Some valuable and effective steps of the WASPAS method are listed below:

***Step 1:*** The input data of the technique is represented in the form of a matrix of alternatives and attributes, which is based on the data received from the expert.

***Step 2:*** Further, we normalize the information in decision matrix by using the below theory:

(47) \begin{eqnarray*}\overline{\overline{{\mathfrak{C}}_{{C}_{oj}}}}= \left( \begin{array}{@{}c@{}} \displaystyle \left[ {\max }_{j}{\overline{\overline{{\mathcal{M}}_{\overline{\overline{{R}_{oj}}}}}}}^{L} \left( \widetilde {{x}_{E}} \right) ~,{\max }_{j}{\overline{\overline{{\mathcal{M}}_{\overline{\overline{{R}_{oj}}}}}}}^{U} \left( \widetilde {{x}_{E}} \right) ~ \right] {e}^{i2\pi \left( \left[ {\max }_{j}{\overline{\overline{{\mathcal{M}}_{\overline{\overline{{I}_{oj}}}}}}}^{L} \left( \widetilde {{x}_{E}} \right) ~,{\max }_{j}{\overline{\overline{{\mathcal{M}}_{\overline{\overline{{I}_{oj}}}}}}}^{U} \left( \widetilde {{x}_{E}} \right) ~ \right] \right) },\\ \displaystyle \left[ {\min }_{j}{\overline{\overline{{\mathcal{N}}_{\overline{\overline{{R}_{0j}}}}}}}^{L} \left( \widetilde {{x}_{E}} \right) ~,{\min }_{j}{\overline{\overline{{\mathcal{N}}_{\overline{\overline{{R}_{0j}}}}}}}^{U} \left( \widetilde {{x}_{E}} \right) ~ \right] {e}^{i2\pi \left( \left[ {\min }_{j}{\overline{\overline{{\mathcal{N}}_{\overline{\overline{{I}_{0j}}}}}}}^{L} \left( \widetilde {{x}_{E}} \right) ~,{\min }_{j}{\overline{\overline{{\mathcal{N}}_{\overline{\overline{{I}_{0j}}}}}}}^{U} \left( \widetilde {{x}_{E}} \right) ~ \right] \right) } \end{array} \right) \end{eqnarray*}

where the data in Eq. (48) is used for benefit types of data, such as

(48) \begin{eqnarray*}{\overline{\overline{{\mathfrak{C}}_{{C}_{ij}}}}}^{/}= \left\{ \begin{array}{@{}cc@{}} \displaystyle 0&\displaystyle otherwise\\ \displaystyle \frac{{\overline{\overline{{\mathcal{M}}_{\overline{\overline{{R}_{ij}}}}}}}^{L}}{1+\overline{{\overline{\overline{{\mathcal{M}}_{\overline{\overline{{R}_{oj}}}}}}}^{L}}} &\displaystyle ~~~ \left( \begin{array}{@{}c@{}} \displaystyle {\overline{\overline{{\mathcal{M}}_{\overline{\overline{{R}_{ij}}}}}}}^{L}\leq {\overline{\overline{{\mathcal{M}}_{\overline{\overline{{R}_{oj}}}}}}}^{L},{\overline{\overline{{\mathcal{M}}_{\overline{\overline{{R}_{ij}}}}}}}^{U}\leq {\overline{\overline{{\mathcal{M}}_{\overline{\overline{{R}_{oj}}}}}}}^{U},\\ \displaystyle {\overline{\overline{{\mathcal{M}}_{\overline{\overline{{I}_{ij}}}}}}}^{L}\leq {\overline{\overline{{\mathcal{M}}_{\overline{\overline{{I}_{oj}}}}}}}^{L},{\overline{\overline{{\mathcal{M}}_{\overline{\overline{{I}_{ij}}}}}}}^{U}\leq {\overline{\overline{{\mathcal{M}}_{\overline{\overline{{I}_{oj}}}}}}}^{U} \end{array} \right) \\ \displaystyle \frac{{\overline{\overline{{\mathcal{N}}_{\overline{\overline{{R}_{ij}}}}}}}^{L}}{1+{\overline{\overline{{\mathcal{N}}_{\overline{\overline{{R}_{oj}}}}}}}^{L}} &\displaystyle for~non-membership~grade \end{array} \right. \end{eqnarray*}

Where the data in Eq. (49) is used for cost types of data, such as

(49) \begin{eqnarray*}{\overline{\overline{{\mathfrak{C}}_{{C}_{ij}}}}}^{/}= \left\{ \begin{array}{@{}cc@{}} \displaystyle 0&\displaystyle otherwise\\ \displaystyle \frac{{\overline{\overline{{\mathcal{M}}_{\overline{\overline{{R}_{ij}}}}}}}^{L}}{1+\overline{{\overline{{\mathcal{M}}_{{\mathfrak{C}}_{{C}_{oj}}}}}^{L}}} &\displaystyle ~~~ \left( \begin{array}{@{}c@{}} \displaystyle {\overline{\overline{{\mathcal{M}}_{\overline{\overline{{R}_{ij}}}}}}}^{L}\leq {\overline{\overline{{\mathcal{M}}_{\overline{\overline{{R}_{oj}}}}}}}^{L},{\overline{\overline{{\mathcal{M}}_{\overline{\overline{{R}_{ij}}}}}}}^{U}\leq {\overline{\overline{{\mathcal{M}}_{\overline{\overline{{R}_{oj}}}}}}}^{U},\\ \displaystyle {\overline{\overline{{\mathcal{M}}_{\overline{\overline{{I}_{ij}}}}}}}^{L}\leq {\overline{\overline{{\mathcal{M}}_{\overline{\overline{{I}_{oj}}}}}}}^{L},{\overline{\overline{{\mathcal{M}}_{\overline{\overline{{I}_{ij}}}}}}}^{U}\leq {\overline{\overline{{\mathcal{M}}_{\overline{\overline{{I}_{oj}}}}}}}^{U} \end{array} \right) \\ \displaystyle \frac{{\overline{\overline{{\mathcal{N}}_{\overline{\overline{{R}_{ij}}}}}}}^{L}}{1+\overline{{\overline{{\mathcal{N}}_{\overline{\overline{{\mathfrak{C}}_{{C}_{oj}}}}}}}^{L}}} &\displaystyle for~non-membership~grade \end{array} \right. \end{eqnarray*}

where ${\mathfrak{C}}_{{C}_{oj}}= \left( {\scriptsize \begin{array}{@{}c@{}} \displaystyle \left[ {\min }_{j}{\overline{\overline{{\mathcal{M}}_{\overline{\overline{{R}_{oj}}}}}}}^{L} \left( \widetilde {{x}_{E}} \right) ~,{\min }_{j}{\overline{\overline{{\mathcal{M}}_{\overline{\overline{{R}_{oj}}}}}}}^{U} \left( \widetilde {{x}_{E}} \right) ~ \right] {e}^{i2\pi \left( \left[ {\min }_{j}{\overline{\overline{{\mathcal{M}}_{\overline{\overline{{I}_{oj}}}}}}}^{L} \left( \widetilde {{x}_{E}} \right) ~,{\min }_{j}{\overline{\overline{{\mathcal{M}}_{\overline{\overline{{I}_{oj}}}}}}}^{U} \left( \widetilde {{x}_{E}} \right) ~ \right] \right) },\\ \displaystyle \left[ {\max }_{j}{\overline{{\mathcal{N}}_{\overline{{R}_{0j}}}}}^{L} \left( \widetilde {{x}_{E}} \right) ~,{\max }_{j}{\overline{{\mathcal{N}}_{\overline{{R}_{0j}}}}}^{U} \left( \widetilde {{x}_{E}} \right) ~ \right] {e}^{i2\pi \left( \left[ {\max }_{j}{\overline{{\mathcal{N}}_{\overline{{I}_{0j}}}}}^{L} \left( \widetilde {{x}_{E}} \right) ~,{\max }_{j}{\overline{{\mathcal{N}}_{\overline{{I}_{0j}}}}}^{U} \left( \widetilde {{x}_{E}} \right) ~ \right] \right) } \end{array}} \right) $.

***Step 3:*** Utilizing *CIVIFAAWA* and *CIVIFAAWG* operators to obtain the WSM and WPM of each alternative:

\begin{eqnarray*}WS{M}_{i}=CIVIFAAWA \left( \overline{\overline{{\mathfrak{C}}_{{C}_{1}}}},\overline{\overline{{\mathfrak{C}}_{{C}_{2}}}},\ldots ,\overline{\overline{{\mathfrak{C}}_{{C}_{n}}}} \right) \end{eqnarray*}

(50) \begin{eqnarray*}= \left( \begin{array}{@{}c@{}} \displaystyle \left[ \left( 1-{e}^{-{ \left( \sum _{j=1}^{n}\overline{\overline{{\mathfrak{W}}_{j}}}{ \left( -\text{log} \left( 1-{\overline{\overline{{\mathcal{M}}_{\overline{\overline{{R}_{j}}}}}}}^{L} \right) ~ \right) }^{\psi } \right) }^{ \frac{1}{\psi } }} \right) , \left( 1-{e}^{-{ \left( \sum _{j=1}^{n}\overline{\overline{{\mathfrak{W}}_{j}}}{ \left( -\text{log} \left( 1-{\overline{\overline{{\mathcal{M}}_{\overline{\overline{{R}_{j}}}}}}}^{U} \right) ~ \right) }^{\psi } \right) }^{ \frac{1}{\psi } }} \right) \right] \\ \displaystyle {e}^{i2\pi \left[ \left( 1-{e}^{-{ \left( \sum _{j=1}^{n}\overline{\overline{{\mathfrak{W}}_{j}}}{ \left( -\text{log} \left( 1-{\overline{\overline{{\mathcal{M}}_{\overline{\overline{{I}_{j}}}}}}}^{L} \right) ~ \right) }^{\psi } \right) }^{ \frac{1}{\psi } }} \right) , \left( 1-{e}^{-{ \left( \sum _{j=1}^{n}\overline{\overline{{\mathfrak{W}}_{j}}}{ \left( -\text{log} \left( 1-{\overline{\overline{{\mathcal{M}}_{\overline{\overline{{I}_{j}}}}}}}^{U} \right) ~ \right) }^{\psi } \right) }^{ \frac{1}{\psi } }} \right) \right] },\\ \displaystyle \left[ \left( {e}^{-{ \left( \sum _{j=1}^{n}\overline{\overline{{\mathfrak{W}}_{j}}}{ \left( -\text{log} \left( {\overline{\overline{{\mathcal{N}}_{\overline{\overline{{R}_{j}}}}}}}^{L} \right) ~ \right) }^{\psi } \right) }^{ \frac{1}{\psi } }} \right) , \left( {e}^{-{ \left( \sum _{j=1}^{n}\overline{\overline{{\mathfrak{W}}_{j}}}{ \left( -\text{log} \left( {\overline{\overline{{\mathcal{N}}_{\overline{\overline{{R}_{j}}}}}}}^{U} \right) ~ \right) }^{\psi } \right) }^{ \frac{1}{\psi } }} \right) \right] \\ \displaystyle {e}^{i2\pi \left[ \left( {e}^{-{ \left( \sum _{j=1}^{n}\overline{\overline{{\mathfrak{W}}_{j}}}{ \left( -\text{log} \left( {\overline{\overline{{\mathcal{N}}_{\overline{\overline{{I}_{j}}}}}}}^{L} \right) ~ \right) }^{\psi } \right) }^{ \frac{1}{\psi } }} \right) , \left( {e}^{-{ \left( \sum _{j=1}^{n}\overline{\overline{{\mathfrak{W}}_{j}}}{ \left( -\text{log} \left( {\overline{\overline{{\mathcal{N}}_{\overline{\overline{{I}_{j}}}}}}}^{U} \right) ~ \right) }^{\psi } \right) }^{ \frac{1}{\psi } }} \right) \right] } \end{array} \right) \end{eqnarray*}

\begin{eqnarray*}WP{M}_{i}=CIVIFAAWG \left( \overline{\overline{{\mathfrak{C}}_{{C}_{1}}}},\overline{\overline{{\mathfrak{C}}_{{C}_{2}}}},\ldots ,\overline{\overline{{\mathfrak{C}}_{{C}_{n}}}} \right) \end{eqnarray*}

(51) \begin{eqnarray*}= \left( \begin{array}{@{}c@{}} \displaystyle \left[ \left( {e}^{-{ \left( \sum _{j=1}^{n}{ \left( -\text{log} \left( {\overline{\overline{{\mathcal{M}}_{\overline{\overline{{R}_{j}}}}}}}^{L} \right) ~ \right) }^{\psi \overline{\overline{{\mathfrak{W}}_{j}}}} \right) }^{ \frac{1}{\psi } }} \right) , \left( {e}^{-{ \left( \sum _{j=1}^{n}{ \left( -\text{log} \left( {\overline{\overline{{\mathcal{M}}_{\overline{\overline{{R}_{j}}}}}}}^{U} \right) ~ \right) }^{\psi \overline{\overline{{\mathfrak{W}}_{j}}}} \right) }^{ \frac{1}{\psi } }} \right) \right] \\ \displaystyle {e}^{i2\pi \left[ \left( {e}^{-{ \left( \sum _{j=1}^{n}{ \left( -\text{log} \left( {\overline{\overline{{\mathcal{M}}_{\overline{\overline{{I}_{j}}}}}}}^{L} \right) ~ \right) }^{\psi \overline{\overline{{\mathfrak{W}}_{j}}}} \right) }^{ \frac{1}{\psi } }} \right) , \left( {e}^{-{ \left( \sum _{j=1}^{n}{ \left( -\text{log} \left( {\overline{\overline{{\mathcal{M}}_{\overline{\overline{{I}_{j}}}}}}}^{U} \right) ~ \right) }^{\psi \overline{\overline{{\mathfrak{W}}_{j}}}} \right) }^{ \frac{1}{\psi } }} \right) \right] },\\ \displaystyle \left[ \left( 1-{e}^{-{ \left( \sum _{j=1}^{n}{ \left( -\text{log} \left( 1-{\overline{\overline{{\mathcal{N}}_{\overline{\overline{{R}_{j}}}}}}}^{L} \right) ~ \right) }^{\psi \overline{\overline{{\mathfrak{W}}_{j}}}} \right) }^{ \frac{1}{\psi } }} \right) , \left( 1-{e}^{-{ \left( \sum _{j=1}^{n}{ \left( -\text{log} \left( 1-{\overline{\overline{{\mathcal{N}}_{\overline{\overline{{R}_{j}}}}}}}^{U} \right) ~ \right) }^{\psi \overline{\overline{{\mathfrak{W}}_{j}}}} \right) }^{ \frac{1}{\psi } }} \right) \right] \\ \displaystyle {e}^{i2\pi \left[ \left( 1-{e}^{-{ \left( \sum _{j=1}^{n}{ \left( -\text{log} \left( 1-{\overline{\overline{{\mathcal{N}}_{\overline{\overline{{I}_{j}}}}}}}^{L} \right) ~ \right) }^{\psi \overline{\overline{{\mathfrak{W}}_{j}}}} \right) }^{ \frac{1}{\psi } }} \right) , \left( 1-{e}^{-{ \left( \sum _{j=1}^{n}{ \left( -\text{log} \left( 1-{\overline{\overline{{\mathcal{N}}_{\overline{\overline{{I}_{j}}}}}}}^{U} \right) ~ \right) }^{\psi \overline{\overline{{\mathfrak{W}}_{j}}}} \right) }^{ \frac{1}{\psi } }} \right) \right] } \end{array} \right) \end{eqnarray*}

***Step 4:*** Compute the score value according to WSM and WPM, the detailed formula is listed as follows.

(52) \begin{eqnarray*}{S}_{i}=\textdegree F\ast \overline{\overline{{\mathcal{S}}_{SV}}} \left( WS{M}_{i} \right) + \left( 1-\textdegree F \right) \ast \overline{\overline{{\mathcal{S}}_{SV}}} \left( WP{M}_{i} \right) \end{eqnarray*}

***Step 5:*** Rank the alternatives and derive the best one referring to the score value *S*_*i*_ in Step 4.

Further, we justify the above-mentioned method by some practical examples.

**Example 1:** To verify the WASPAS technique under the consideration of some CIVIF information, we applied it for practical CIVIF decision matrix to obtain the best alternative. Four alternatives: *S*_1_, *S*_2_, *S*_3_, *S*_4_; and four criteria *C*_1_, *C*_2_, *C*_3_, *C*_4_, and *C*_*IJ*_ indicates the assessment information of *S*_*I*_(*I* = 1, 2, 3, 4) under the criterion ${C}_{J} \left( J=1,2,~3,~4 \right) .$ Some valuable and effective steps of the WASPAS method are listed below:

***Step 1:*** The input data of the technique is represented in the form of a matrix of alternatives and attributes, which is based on the data received from the expert.

\begin{eqnarray*}\overline{\overline{{\mathfrak{C}}_{{C}_{ij}}}}= \left[ \begin{array}{@{}c@{}} \displaystyle \left( \begin{array}{@{}c@{}} \displaystyle \left[ 0.3,0.6 \right] {e}^{i2\pi \left( \left[ 0.1,0.3 \right] \right) },\\ \displaystyle \left[ 0.3,0.4 \right] {e}^{i2\pi \left( \left[ 0.3,0.3 \right] \right) } \end{array} \right) \\ \displaystyle \left( \begin{array}{@{}c@{}} \displaystyle \left[ 0.2,0.3 \right] {e}^{i2\pi \left( \left[ 0.6,0.7 \right] \right) },\\ \displaystyle \left[ 0.3,0.4 \right] {e}^{i2\pi \left( \left[ 0.2,0.3 \right] \right) } \end{array} \right) \\ \displaystyle \begin{array}{@{}c@{}} \displaystyle \left( \begin{array}{@{}c@{}} \displaystyle \left[ 0.1,0.3 \right] {e}^{i2\pi \left( \left[ 0.3,0.4 \right] \right) },\\ \displaystyle \left[ 0.1,0.2 \right] {e}^{i2\pi \left( \left[ 0.1,0.2 \right] \right) } \end{array} \right) \\ \displaystyle \left( \begin{array}{@{}c@{}} \displaystyle \left[ 0.3,0.4 \right] {e}^{i2\pi \left( \left[ 0.3,0.3 \right] \right) },\\ \displaystyle \left[ 0.2,0.2 \right] {e}^{i2\pi \left( \left[ 0.1,0.2 \right] \right) } \end{array} \right) \end{array} \end{array}~~\begin{array}{@{}c@{}} \displaystyle \left( \begin{array}{@{}c@{}} \displaystyle \left[ 0.2,0.3 \right] {e}^{i2\pi \left( \left[ 0.2,0.4 \right] \right) },\\ \displaystyle \left[ 0.3,0.5 \right] {e}^{i2\pi \left( \left[ 0.3,0.4 \right] \right) } \end{array} \right) \\ \displaystyle \left( \begin{array}{@{}c@{}} \displaystyle \left[ 0.3,0.4 \right] {e}^{i2\pi \left( \left[ 0.5,0.6 \right] \right) },\\ \displaystyle \left[ 0.2,0.3 \right] {e}^{i2\pi \left( \left[ 0.1,0.3 \right] \right) } \end{array} \right) \\ \displaystyle \begin{array}{@{}c@{}} \displaystyle \left( \begin{array}{@{}c@{}} \displaystyle \left[ 0.2,0.4 \right] {e}^{i2\pi \left( \left[ 0.3,0.5 \right] \right) },\\ \displaystyle \left[ 0.2,0.3 \right] {e}^{i2\pi \left( \left[ 0.2,0.2 \right] \right) } \end{array} \right) \\ \displaystyle \left( \begin{array}{@{}c@{}} \displaystyle \left[ 0.3,0.5 \right] {e}^{i2\pi \left( \left[ 0.3,0.4 \right] \right) },\\ \displaystyle \left[ 0.2,0.2 \right] {e}^{i2\pi \left( \left[ 0.1,0.2 \right] \right) } \end{array} \right) \end{array} \end{array}~~\begin{array}{@{}c@{}} \displaystyle \left( \begin{array}{@{}c@{}} \displaystyle \left[ 0.3,0.4 \right] {e}^{i2\pi \left( \left[ 0.1,0.3 \right] \right) },\\ \displaystyle \left[ 0.2,0.2 \right] {e}^{i2\pi \left( \left[ 0.2,0.3 \right] \right) } \end{array} \right) \\ \displaystyle \left( \begin{array}{@{}c@{}} \displaystyle \left[ 0.4,0.5 \right] {e}^{i2\pi \left( \left[ 0.4,0.5 \right] \right) },\\ \displaystyle \left[ 0.3,0.4 \right] {e}^{i2\pi \left( \left[ 0.2,0.2 \right] \right) } \end{array} \right) \\ \displaystyle \begin{array}{@{}c@{}} \displaystyle \left( \begin{array}{@{}c@{}} \displaystyle \left[ 0.1,0.3 \right] {e}^{i2\pi \left( \left[ 0.2,0.2 \right] \right) },\\ \displaystyle \left[ 0.3,0.3 \right] {e}^{i2\pi \left( \left[ 0.2,0.3 \right] \right) } \end{array} \right) \\ \displaystyle \left( \begin{array}{@{}c@{}} \displaystyle \left[ 0.2,0.2 \right] {e}^{i2\pi \left( \left[ 0.2,0.3 \right] \right) },\\ \displaystyle \left[ 0.2,0.2 \right] {e}^{i2\pi \left( \left[ 0.1,0.2 \right] \right) } \end{array} \right) \end{array} \end{array}~~\begin{array}{@{}c@{}} \displaystyle \left( \begin{array}{@{}c@{}} \displaystyle \left[ 0.1,0.2 \right] {e}^{i2\pi \left( \left[ 0.2,0.2 \right] \right) },\\ \displaystyle \left[ 0.1,0.1 \right] {e}^{i2\pi \left( \left[ 0.1,0.3 \right] \right) } \end{array} \right) \\ \displaystyle \left( \begin{array}{@{}c@{}} \displaystyle \left[ 0.5,0.6 \right] {e}^{i2\pi \left( \left[ 0.3,0.4 \right] \right) },\\ \displaystyle \left[ 0.2,0.4 \right] {e}^{i2\pi \left( \left[ 0.2,0.2 \right] \right) } \end{array} \right) \\ \displaystyle \begin{array}{@{}c@{}} \displaystyle \left( \begin{array}{@{}c@{}} \displaystyle \left[ 0.2,0.2 \right] {e}^{i2\pi \left( \left[ 0.1,0.1 \right] \right) },\\ \displaystyle \left[ 0.3,0.4 \right] {e}^{i2\pi \left( \left[ 0.2,0.3 \right] \right) } \end{array} \right) \\ \displaystyle \left( \begin{array}{@{}c@{}} \displaystyle \left[ 0.1,0.1 \right] {e}^{i2\pi \left( \left[ 0.1,0.3 \right] \right) },\\ \displaystyle \left[ 0.1,0.2 \right] {e}^{i2\pi \left( \left[ 0.1,0.2 \right] \right) } \end{array} \right) \end{array} \end{array} \right] \end{eqnarray*}

***Step 2:*** Further, we normalize the information in decision matrix by using Eqs. (47)–49, and obtain following resutls:

\begin{eqnarray*}\overline{\overline{{\mathfrak{C}}_{{C}_{0,j}}}}= \left\{ \left( \begin{array}{@{}c@{}} \displaystyle \left[ 0.3,0.6 \right] {e}^{i2\pi \left( \left[ 0.2,0.4 \right] \right) },\\ \displaystyle \left[ 0.1,0.1 \right] {e}^{i2\pi \left( \left[ 0.1,0.3 \right] \right) } \end{array} \right) , \left( \begin{array}{@{}c@{}} \displaystyle \left[ 0.5,0.6 \right] {e}^{i2\pi \left( \left[ 0.6,0.7 \right] \right) },\\ \displaystyle \left[ 0.2,0.3 \right] {e}^{i2\pi \left( \left[ 0.1,0.2 \right] \right) } \end{array} \right) , \left( \begin{array}{@{}c@{}} \displaystyle \left[ 0.2,0.4 \right] {e}^{i2\pi \left( \left[ 0.3,0.5 \right] \right) },\\ \displaystyle \left[ 0.1,0.2 \right] {e}^{i2\pi \left( \left[ 0.1,0.2 \right] \right) } \end{array} \right) , \left( \begin{array}{@{}c@{}} \displaystyle \left[ 0.3,0.5 \right] {e}^{i2\pi \left( \left[ 0.3,0.4 \right] \right) },\\ \displaystyle \left[ 0.1,0.2 \right] {e}^{i2\pi \left( \left[ 0.1,0.2 \right] \right) } \end{array} \right) \right\} \end{eqnarray*}

\begin{eqnarray*}{\overline{\overline{{\mathfrak{C}}_{{C}_{ij}}}}}^{/}= \left[ \begin{array}{@{}c@{}} \displaystyle \left( \begin{array}{@{}c@{}} \displaystyle \left[ 0.231,0.375 \right] \\ \displaystyle {e}^{i2\pi \left( \left[ 0.083,0.214 \right] \right) },\\ \displaystyle \left[ 0.273,0.364 \right] \\ \displaystyle {e}^{i2\pi \left( \left[ 0.273,0.231 \right] \right) } \end{array} \right) \\ \displaystyle \left( \begin{array}{@{}c@{}} \displaystyle \left[ 0.133,0.187 \right] \\ \displaystyle {e}^{i2\pi \left( \left[ 0.375,0.412 \right] \right) },\\ \displaystyle \left[ 0.25,0.308 \right] \\ \displaystyle {e}^{i2\pi \left( \left[ 0.182,0.25 \right] \right) } \end{array} \right) \\ \displaystyle \begin{array}{@{}c@{}} \displaystyle \left( \begin{array}{@{}c@{}} \displaystyle \left[ 0.084,0.214 \right] \\ \displaystyle {e}^{i2\pi \left( \left[ 0.230,0.267 \right] \right) },\\ \displaystyle \left[ 0.091,0.167 \right] \\ \displaystyle {e}^{i2\pi \left( \left[ 0.091,0.167 \right] \right) } \end{array} \right) \\ \displaystyle \left( \begin{array}{@{}c@{}} \displaystyle \left[ 0.230,0.267 \right] \\ \displaystyle {e}^{i2\pi \left( \left[ 0.230,0.214 \right] \right) },\\ \displaystyle \left[ 0.182,0.167 \right] \\ \displaystyle {e}^{i2\pi \left( \left[ 0.091,0.167 \right] \right) } \end{array} \right) \end{array} \end{array}~~\begin{array}{@{}c@{}} \displaystyle \left( \begin{array}{@{}c@{}} \displaystyle \left[ 0.154,0.187 \right] {e}^{i2\pi \left( \left[ 0.167,0.285 \right] \right) },\\ \displaystyle \left[ 0.273,0.455 \right] {e}^{i2\pi \left( \left[ 0.273,0.307 \right] \right) } \end{array} \right) \\ \displaystyle \left( \begin{array}{@{}c@{}} \displaystyle \left[ 0.2,0.25 \right] {e}^{i2\pi \left( \left[ 0.312,0.353 \right] \right) },\\ \displaystyle \left[ 0.167,0.231 \right] {e}^{i2\pi \left( \left[ 0.091,0.25 \right] \right) } \end{array} \right) \\ \displaystyle \begin{array}{@{}c@{}} \displaystyle \left( \begin{array}{@{}c@{}} \displaystyle \left[ 0.167,0.285 \right] {e}^{i2\pi \left( \left[ 0.230,0.333 \right] \right) },\\ \displaystyle \left[ 0.182,0.25 \right] {e}^{i2\pi \left( \left[ 0.182,0.167 \right] \right) } \end{array} \right) \\ \displaystyle \left( \begin{array}{@{}c@{}} \displaystyle \left[ 0.230,0.334 \right] {e}^{i2\pi \left( \left[ 0.230,0.285 \right] \right) },\\ \displaystyle \left[ 0.182,0.167 \right] {e}^{i2\pi \left( \left[ 0.091,0.167 \right] \right) } \end{array} \right) \end{array} \end{array}~~\begin{array}{@{}c@{}} \displaystyle \left( \begin{array}{@{}c@{}} \displaystyle \left[ 0.231,0.25 \right] {e}^{i2\pi \left( \left[ 0.083,0.214 \right] \right) },\\ \displaystyle \left[ 0.182,0.182 \right] {e}^{i2\pi \left( \left[ 0.182,0.231 \right] \right) } \end{array} \right) \\ \displaystyle \left( \begin{array}{@{}c@{}} \displaystyle \left[ 0.267,0.312 \right] {e}^{i2\pi \left( \left[ 0.25,0.294 \right] \right) },\\ \displaystyle \left[ 0.25,0.308 \right] {e}^{i2\pi \left( \left[ 0.182,0.167 \right] \right) } \end{array} \right) \\ \displaystyle \begin{array}{@{}c@{}} \displaystyle \left( \begin{array}{@{}c@{}} \displaystyle \left[ 0.083,0.214 \right] {e}^{i2\pi \left( \left[ 0.153,0.133 \right] \right) },\\ \displaystyle \left[ 0.273,0.25 \right] {e}^{i2\pi \left( \left[ 0.182,0.25 \right] \right) } \end{array} \right) \\ \displaystyle \left( \begin{array}{@{}c@{}} \displaystyle \left[ 0.153,0.133 \right] {e}^{i2\pi \left( \left[ 0.153,0.214 \right] \right) },\\ \displaystyle \left[ 0.182,0.167 \right] {e}^{i2\pi \left( \left[ 0.091,0.167 \right] \right) } \end{array} \right) \end{array} \end{array}~~\begin{array}{@{}c@{}} \displaystyle \left( \begin{array}{@{}c@{}} \displaystyle \left[ 0.077,0.125 \right] \\ \displaystyle {e}^{i2\pi \left( \left[ 0.167,0.143 \right] \right) },\\ \displaystyle \left[ 0.,0. \right] {e}^{i2\pi \left( \left[ 0.,0. \right] \right) } \end{array} \right) \\ \displaystyle \left( \begin{array}{@{}c@{}} \displaystyle \left[ 0.333,0.375 \right] \\ \displaystyle {e}^{i2\pi \left( \left[ 0.187,0.2353 \right] \right) },\\ \displaystyle \left[ 0.167,0.308 \right] \\ \displaystyle {e}^{i2\pi \left( \left[ 0.182,0.167 \right] \right) } \end{array} \right) \\ \displaystyle \begin{array}{@{}c@{}} \displaystyle \left( \begin{array}{@{}c@{}} \displaystyle \left[ 0.167,0.142 \right] \\ \displaystyle {e}^{i2\pi \left( \left[ 0.076,0.067 \right] \right) },\\ \displaystyle \left[ 0.273,0.333 \right] \\ \displaystyle {e}^{i2\pi \left( \left[ 0.182,0.25 \right] \right) } \end{array} \right) \\ \displaystyle \left( \begin{array}{@{}c@{}} \displaystyle \left[ 0.076,0.067 \right] \\ \displaystyle {e}^{i2\pi \left( \left[ 0.076,0.214 \right] \right) },\\ \displaystyle \left[ 0.091,0.167 \right] \\ \displaystyle {e}^{i2\pi \left( \left[ 0.091,0.167 \right] \right) } \end{array} \right) \end{array} \end{array} \right] \end{eqnarray*}

***Step 3:*** Compute the WSM and WPM of each alternative according to the *CIVIFAAWA* and *CIVIFAAWG* operators (*ψ* = 1), the specific results are illustrated as follows.

\begin{eqnarray*}WS{M}_{i}= \left\{ \begin{array}{@{}c@{}} \displaystyle \left( \begin{array}{@{}c@{}} \displaystyle \left[ 0.000028,0.0011 \right] {e}^{i2\pi \left( \left[ 0.000007,0.00009 \right] \right) },\\ \displaystyle \left[ 0.8096,0.9050 \right] {e}^{i2\pi \left( \left[ 0.8096,0.8925 \right] \right) } \end{array} \right) , \left( \begin{array}{@{}c@{}} \displaystyle \left[ 0.0001,0.0003 \right] {e}^{i2\pi \left( \left[ 0.0003,0.0007 \right] \right) },\\ \displaystyle \left[ 0.828,0.9292 \right] {e}^{i2\pi \left( \left[ 0.6953,0.828 \right] \right) } \end{array} \right) ,\\ \displaystyle \left( \begin{array}{@{}c@{}} \displaystyle \left[ 0.000007,0.00009 \right] {e}^{i2\pi \left( \left[ 0.00002,0.00003 \right] \right) },\\ \displaystyle \left[ 0.8096,0.8907 \right] {e}^{i2\pi \left( \left[ 0.6952,0.8279 \right] \right) } \end{array} \right) , \left( \begin{array}{@{}c@{}} \displaystyle \left[ 0.00003,0.00004 \right] {e}^{i2\pi \left( \left[ 0.00003,0.0001 \right] \right) },\\ \displaystyle \left[ 0.6953,0.7296 \right] {e}^{i2\pi \left( \left[ 0.3637,0.7296 \right] \right) } \end{array} \right) \end{array} \right\} \end{eqnarray*}

\begin{eqnarray*}WP{M}_{i}= \left\{ \begin{array}{@{}c@{}} \displaystyle \left( \begin{array}{@{}c@{}} \displaystyle \left[ 0.7291,0.8651 \right] {e}^{i2\pi \left( \left[ 0.5452,0.8377 \right] \right) },\\ \displaystyle \left[ 0.00005,0.000160 \right] {e}^{i2\pi \left( \left[ 0.000059,0.00020 \right] \right) } \end{array} \right) , \left( \begin{array}{@{}c@{}} \displaystyle \left[ 0.8658,0.9222 \right] {e}^{i2\pi \left( \left[ 0.9222,0.9512 \right] \right) },\\ \displaystyle \left[ 0.00008,0.0004 \right] {e}^{i2\pi \left( \left[ 0.00002,0.00008 \right] \right) } \end{array} \right) ,\\ \displaystyle \left( \begin{array}{@{}c@{}} \displaystyle \left[ 0.5452,0.8377 \right] {e}^{i2\pi \left( \left[ 0.7292,0.7847 \right] \right) },\\ \displaystyle \left[ 0.00005,0.000187 \right] {e}^{i2\pi \left( \left[ 0.00002,0.00008 \right] \right) } \end{array} \right) , \left( \begin{array}{@{}c@{}} \displaystyle \left[ 0.7292,0.7847 \right] {e}^{i2\pi \left( \left[ 0.7292,0.8693 \right] \right) },\\ \displaystyle \left[ 0.00002,0.00003 \right] {e}^{i2\pi \left( \left[ 0.000003,0.00003 \right] \right) } \end{array} \right) \end{array} \right\} \end{eqnarray*}

Further, we examine the values of the score function, such as:

\begin{eqnarray*}WS{M}_{i}= \left\{ -0.8542,-0.82,-0.8058,-0.629 \right\} \end{eqnarray*}

\begin{eqnarray*}WP{M}_{i}= \left\{ 0.7442,0.9152,0.7241,0.7781 \right\} \end{eqnarray*}

***Step 4:*** Acquire the score value of each alternative by using the theory of WSM and WPM information:

\begin{eqnarray*}{S}_{1}=0.4245,{S}_{2}=0.5682,{S}_{3}=0.4181,{S}_{4}=0.4966 \end{eqnarray*}

For convenience, we assume °*F* = 0.2.

***Step 5:*** Identify the ranking information for evaluating or deriving the best preference.

\begin{eqnarray*}{S}_{2}\geq {S}_{4}\geq {S}_{1}\geq {S}_{3} \end{eqnarray*}

From the above analysis, we obtain the best preferences as *S*_2_.

### Application in MADM

The significant commitment of this examination is to apply MADM method under CIVIFS for deciding the optimal scheme from the group of complex interval-valued intuitionistic fuzzy data. To determine the best one, we expounded a dynamic interaction. There are *m* alternative $\overline{\overline{{\mathfrak{C}}_{C}}}= \left\{ \overline{\overline{{\mathfrak{C}}_{{C}_{1}}}},\overline{\overline{{\mathfrak{C}}_{{C}_{2}}}},\ldots ,\overline{\overline{{\mathfrak{C}}_{{C}_{m}}}} \right\} $ and *n* criteria looking like

### Procedure of decision-making

To achieve the acquirement of the best one, we built the dynamic calculation looking like the accompanying stages:

Stage 1: Construct the CIVIF decision matrix utilizing the CIVIF evaluation information.

Stage 2: Normalize the CIVIF decision matrix. The specific conversion process is shown below when dealing with beneficial data and cost data:

\begin{eqnarray*}D= \left\{ \begin{array}{@{}cc@{}} \displaystyle \left( \left[ {\overline{\overline{{\mathcal{M}}_{\overline{{R}_{jk}}}}}}^{L},{\overline{\overline{{\mathcal{M}}_{\overline{\overline{{R}_{jk}}}}}}}^{U} \right] {e}^{i2\pi \left( \left[ {\overline{\overline{{\mathcal{M}}_{\overline{\overline{{I}_{jk}}}}}}}^{L},{\overline{\overline{{\mathcal{M}}_{\overline{\overline{{I}_{jk}}}}}}}^{U} \right] \right) }, \left[ {\overline{\overline{{\mathcal{N}}_{\overline{{R}_{jk}}}}}}^{L},{\overline{\overline{{\mathcal{N}}_{\overline{\overline{{R}_{jk}}}}}}}^{U} \right] {e}^{i2\pi \left( \left[ {\overline{\overline{{\mathcal{N}}_{\overline{\overline{{I}_{jk}}}}}}}^{L},{\overline{\overline{{\mathcal{N}}_{\overline{\overline{{I}_{jk}}}}}}}^{U} \right] \right) } \right) &\displaystyle for~benefit~sort~of~data\\ \displaystyle \left( \left[ {\overline{\overline{{\mathcal{N}}_{\overline{\overline{{R}_{jk}}}}}}}^{L},{\overline{\overline{{\mathcal{N}}_{\overline{\overline{{R}_{jk}}}}}}}^{U} \right] {e}^{i2\pi \left( \left[ {\overline{\overline{{\mathcal{N}}_{\overline{\overline{{I}_{jk}}}}}}}^{L},{\overline{\overline{{\mathcal{N}}_{\overline{\overline{{I}_{jk}}}}}}}^{U} \right] \right) }, \left[ {\overline{\overline{{\mathcal{M}}_{\overline{\overline{{R}_{jk}}}}}}}^{L},{\overline{\overline{{\mathcal{M}}_{\overline{\overline{{R}_{jk}}}}}}}^{U} \right] {e}^{i2\pi \left( \left[ {\overline{\overline{{\mathcal{M}}_{\overline{\overline{{I}_{jk}}}}}}}^{L},{\overline{\overline{{\mathcal{M}}_{\overline{\overline{{I}_{jk}}}}}}}^{U} \right] \right) } \right) &\displaystyle for~cost~sort~of~data \end{array} \right. \end{eqnarray*}

Stage 3: Utilizing the Eq. (17) (CIVIFAAWA) and Eq. (32) (CIVIFAAWG) to aggregate the information in the decision matrix.

Stage 4: Using Eq. (6) to derive the score information.

Stage 5: Evaluate the ranking information in the availability of score information.

### Represented example

The significant finding of this investigation is to break down the explained administrators in the conditions of the MADM methodology. For this, we examined some pragmatic information to decide the practicality and probability of the introduced works.

### Clarification of the problem

Permit us to ponder a creation association that expects to enroll a publicizing director for an unfilled post. Here, we consider five competitors $\overline{\overline{{\mathfrak{C}}_{{C}_{j}}}},j=1,2,3,4,5$, allocated for extra appraisals, such as: $\overline{\overline{{\mathfrak{C}}_{{C}_{1}}^{{}^{{^{\prime}}}}}}:$ Oral presentation capacity; $\overline{\overline{{\mathfrak{C}}_{{C}_{2}}^{{}^{{^{\prime}}}}}}:$ History; $\overline{\overline{{\mathfrak{C}}_{{C}_{3}}^{{}^{{^{\prime}}}}}}:$ Overall tendency; and $\overline{\overline{{\mathfrak{C}}_{{C}_{4}}^{{}^{{^{\prime}}}}}}$: confidence. For this, we consider weight vectors such as 0.4,0.3,0.2,0.1. The five specialists $\overline{\overline{{\mathfrak{C}}_{{C}_{j}}}},j=1,2,3,4,5$ are to oversee vagueness under CIVIF information by utilizing dynamic strategies.

### Method under CIVIFAAWA and CIVIFAAWG operators

Determine the useful individual from the gathering of people (Five up-and-comers) by utilizing the MADM procedure under CIVIFAAWA and CIVIFAAWG operators. For obtaining the ideal one, we developed the dynamic calculation looking like the accompanying stages:

Stage 1: Construct the CIVIF decision matrix. The specific data covering the cost and beneficial sorts are listed as Table 2.

**Table 2:** CIVIF data.

	${\overline{\overline{{\mathfrak{C}}_{{C}_{1}}}}}^{{}^{{^{\prime}}}}$	${\overline{\overline{{\mathfrak{C}}_{{C}_{2}}}}}^{{}^{{^{\prime}}}}$
$\overline{\overline{{\mathfrak{C}}_{{C}_{1}}}}$	$ \left( \left[ 0.4,0.5 \right] {e}^{i2\pi \left( \left[ 0.3,0.4 \right] \right) }, \left[ 0.2,0.3 \right] {e}^{i2\pi \left( \left[ 0.2,0.4 \right] \right) } \right) $	$ \left( \left[ 0.41,0.51 \right] {e}^{i2\pi \left( \left[ 0.31,0.41 \right] \right) }, \left[ 0.21,0.31 \right] {e}^{i2\pi \left( \left[ 0.21,0.41 \right] \right) } \right) $
$\overline{\overline{{\mathfrak{C}}_{{C}_{2}}}}$	$ \left( \left[ 0.3,0.4 \right] {e}^{i2\pi \left( \left[ 0.0,0.1 \right] \right) }, \left[ 0.0,0.1 \right] {e}^{i2\pi \left( \left[ 0.0,0.1 \right] \right) } \right) $	$ \left( \left[ 0.31,0.41 \right] {e}^{i2\pi \left( \left[ 0.01,0.11 \right] \right) }, \left[ 0.01,0.11 \right] {e}^{i2\pi \left( \left[ 0.01,0.11 \right] \right) } \right) $
$\overline{\overline{{\mathfrak{C}}_{{C}_{3}}}}$	$ \left( \left[ 0.4,0.5 \right] {e}^{i2\pi \left( \left[ 0.1,0.2 \right] \right) }, \left[ 0.3,0.4 \right] {e}^{i2\pi \left( \left[ 0.2,0.3 \right] \right) } \right) $	$ \left( \left[ 0.41,0.51 \right] {e}^{i2\pi \left( \left[ 0.11,0.21 \right] \right) }, \left[ 0.31,0.41 \right] {e}^{i2\pi \left( \left[ 0.21,0.31 \right] \right) } \right) $
$\overline{\overline{{\mathfrak{C}}_{{C}_{4}}}}$	$ \left( \left[ 0.2,0.3 \right] {e}^{i2\pi \left( \left[ 0.4,0.5 \right] \right) }, \left[ 0.0,0.1 \right] {e}^{i2\pi \left( \left[ 0.1,0.2 \right] \right) } \right) $	$ \left( \left[ 0.21,0.31 \right] {e}^{i2\pi \left( \left[ 0.41,0.51 \right] \right) }, \left[ 0.01,0.11 \right] {e}^{i2\pi \left( \left[ 0.11,0.21 \right] \right) } \right) $
$\overline{\overline{{\mathfrak{C}}_{{C}_{5}}}}$	$ \left( \left[ 0.4,0.5 \right] {e}^{i2\pi \left( \left[ 0.4,0.5 \right] \right) }, \left[ 0.0,0.1 \right] {e}^{i2\pi \left( \left[ 0.2,0.3 \right] \right) } \right) $	$ \left( \left[ 0.41,0.51 \right] {e}^{i2\pi \left( \left[ 0.41,0.51 \right] \right) }, \left[ 0.01,0.11 \right] {e}^{i2\pi \left( \left[ 0.21,0.31 \right] \right) } \right) $
	${\overline{\overline{{\mathfrak{C}}_{{C}_{3}}}}}^{{}^{{^{\prime}}}}$	${\overline{\overline{{\mathfrak{C}}_{{C}_{4}}}}}^{{}^{{^{\prime}}}}$
$\overline{\overline{{\mathfrak{C}}_{{C}_{1}}}}$	$ \left( \left[ 0.42,0.52 \right] {e}^{i2\pi \left( \left[ 0.32,0.42 \right] \right) }, \left[ 0.22,0.32 \right] {e}^{i2\pi \left( \left[ 0.22,0.42 \right] \right) } \right) $	$ \left( \left[ 0.43,0.53 \right] {e}^{i2\pi \left( \left[ 0.33,0.43 \right] \right) }, \left[ 0.23,0.33 \right] {e}^{i2\pi \left( \left[ 0.23,0.43 \right] \right) } \right) $
$\overline{\overline{{\mathfrak{C}}_{{C}_{2}}}}$	$ \left( \left[ 0.32,0.42 \right] {e}^{i2\pi \left( \left[ 0.02,0.12 \right] \right) }, \left[ 0.02,0.12 \right] {e}^{i2\pi \left( \left[ 0.02,0.12 \right] \right) } \right) $	$ \left( \left[ 0.33,0.43 \right] {e}^{i2\pi \left( \left[ 0.03,0.13 \right] \right) }, \left[ 0.03,0.13 \right] {e}^{i2\pi \left( \left[ 0.03,0.13 \right] \right) } \right) $
$\overline{\overline{{\mathfrak{C}}_{{C}_{3}}}}$	$ \left( \left[ 0.42,0.52 \right] {e}^{i2\pi \left( \left[ 0.12,0.22 \right] \right) }, \left[ 0.32,0.42 \right] {e}^{i2\pi \left( \left[ 0.22,0.32 \right] \right) } \right) $	$ \left( \left[ 0.43,0.53 \right] {e}^{i2\pi \left( \left[ 0.13,0.23 \right] \right) }, \left[ 0.33,0.43 \right] {e}^{i2\pi \left( \left[ 0.23,0.33 \right] \right) } \right) $
$\overline{\overline{{\mathfrak{C}}_{{C}_{4}}}}$	$ \left( \left[ 0.22,0.32 \right] {e}^{i2\pi \left( \left[ 0.42,0.52 \right] \right) }, \left[ 0.02,0.12 \right] {e}^{i2\pi \left( \left[ 0.12,0.22 \right] \right) } \right) $	$ \left( \left[ 0.23,0.33 \right] {e}^{i2\pi \left( \left[ 0.43,0.53 \right] \right) }, \left[ 0.03,0.13 \right] {e}^{i2\pi \left( \left[ 0.13,0.23 \right] \right) } \right) $
$\overline{\overline{{\mathfrak{C}}_{{C}_{5}}}}$	$ \left( \left[ 0.42,0.52 \right] {e}^{i2\pi \left( \left[ 0.42,0.52 \right] \right) }, \left[ 0.02,0.12 \right] {e}^{i2\pi \left( \left[ 0.22,0.32 \right] \right) } \right) $	$ \left( \left[ 0.43,0.53 \right] {e}^{i2\pi \left( \left[ 0.43,0.53 \right] \right) }, \left[ 0.03,0.13 \right] {e}^{i2\pi \left( \left[ 0.23,0.33 \right] \right) } \right) $

Stage 2: Normalize the decision matrix referring to the subsequent conversion process.

\begin{eqnarray*}D= \left\{ \begin{array}{@{}cc@{}} \displaystyle \left( \left[ {\overline{\overline{{\mathcal{M}}_{\overline{\overline{{R}_{jk}}}}}}}^{L},{\overline{\overline{{\mathcal{M}}_{\overline{\overline{{R}_{jk}}}}}}}^{U} \right] {e}^{i2\pi \left( \left[ {\overline{\overline{{\mathcal{M}}_{\overline{\overline{{I}_{jk}}}}}}}^{L},{\overline{\overline{{\mathcal{M}}_{\overline{\overline{{I}_{jk}}}}}}}^{U} \right] \right) }, \left[ {\overline{\overline{{\mathcal{N}}_{\overline{\overline{{R}_{jk}}}}}}}^{L},{\overline{\overline{{\mathcal{N}}_{\overline{\overline{{R}_{jk}}}}}}}^{U} \right] {e}^{i2\pi \left( \left[ {\overline{\overline{{\mathcal{N}}_{\overline{\overline{{I}_{jk}}}}}}}^{L},{\overline{\overline{{\mathcal{N}}_{\overline{\overline{{I}_{jk}}}}}}}^{U} \right] \right) } \right) &\displaystyle for~benefit~sort~of~data\\ \displaystyle \left( \left[ {\overline{\overline{{\mathcal{N}}_{\overline{\overline{{R}_{jk}}}}}}}^{L},{\overline{\overline{{\mathcal{N}}_{\overline{\overline{{R}_{jk}}}}}}}^{U} \right] {e}^{i2\pi \left( \left[ {\overline{\overline{{\mathcal{N}}_{\overline{\overline{{I}_{jk}}}}}}}^{L},{\overline{\overline{{\mathcal{N}}_{\overline{\overline{{I}_{jk}}}}}}}^{U} \right] \right) }, \left[ {\overline{\overline{{\mathcal{M}}_{\overline{\overline{{R}_{jk}}}}}}}^{L},{\overline{\overline{{\mathcal{M}}_{\overline{\overline{{R}_{jk}}}}}}}^{U} \right] {e}^{i2\pi \left( \left[ {\overline{\overline{{\mathcal{M}}_{\overline{\overline{{I}_{jk}}}}}}}^{L},{\overline{\overline{{\mathcal{M}}_{\overline{\overline{{I}_{jk}}}}}}}^{U} \right] \right) } \right) &\displaystyle for~cost~sort~of~data \end{array} \right. \end{eqnarray*}

Stage 3: Under the CIVIFAAWA and CIVIFAAWG operators, we obtain the aggregation consequence shown in Table 3 (*ψ* = 1).

**Table 3:** Aggregation information matric.

	***CIV IFAAWA operator***	***CIV IFAAWG operator***
$\overline{\overline{{\mathfrak{C}}_{{C}_{1}}}}$	$ \left( \begin{array}{@{}c@{}} \displaystyle \left[ 0.2048,0.2665 \right] {e}^{i2\pi \left( \left[ 0.1489,0.2048 \right] \right) },\\ \displaystyle \left[ 0.5075,0.6012 \right] {e}^{i2\pi \left( \left[ 0.6012,0.6789 \right] \right) } \end{array} \right) $	$ \left( \begin{array}{@{}c@{}} \displaystyle \left[ 0.6789,0.7464 \right] {e}^{i2\pi \left( \left[ 0.6012,0.6789 \right] \right) },\\ \displaystyle \left[ 0.0973,0.1489 \right] {e}^{i2\pi \left( \left[ 0.1489,0.2048 \right] \right) } \end{array} \right) $
$\overline{\overline{{\mathfrak{C}}_{{C}_{2}}}}$	$ \left( \begin{array}{@{}c@{}} \displaystyle \left[ 0.1489,0.2048 \right] {e}^{i2\pi \left( \left[ 0.0048,0.0494 \right] \right) },\\ \displaystyle \left[ 0.1029,0.3828 \right] {e}^{i2\pi \left( \left[ 0.1029,0.3828 \right] \right) } \end{array} \right) $	$ \left( \begin{array}{@{}c@{}} \displaystyle \left[ 0.6012,0.6789 \right] {e}^{i2\pi \left( \left[ 0.1029,0.3828 \right] \right) },\\ \displaystyle \left[ 0.0048,0.0494 \right] {e}^{i2\pi \left( \left[ 0.0048,0.0494 \right] \right) } \end{array} \right) $
$\overline{\overline{{\mathfrak{C}}_{{C}_{3}}}}$	$ \left( \begin{array}{@{}c@{}} \displaystyle \left[ 0.2048,0.2665 \right] {e}^{i2\pi \left( \left[ 0.0494,0.0973 \right] \right) },\\ \displaystyle \left[ 0.6012,0.6789 \right] {e}^{i2\pi \left( \left[ 0.5075,0.6012 \right] \right) } \end{array} \right) $	$ \left( \begin{array}{@{}c@{}} \displaystyle \left[ 0.6789,0.7464 \right] {e}^{i2\pi \left( \left[ 0.3828,0.5075 \right] \right) },\\ \displaystyle \left[ 0.1489,0.2048 \right] {e}^{i2\pi \left( \left[ 0.0973,0.1489 \right] \right) } \end{array} \right) $
$\overline{\overline{{\mathfrak{C}}_{{C}_{4}}}}$	$ \left( \begin{array}{@{}c@{}} \displaystyle \left[ 0.0973,0.1489 \right] {e}^{i2\pi \left( \left[ 0.2048,0.2665 \right] \right) },\\ \displaystyle \left[ 0.1029,0.3828 \right] {e}^{i2\pi \left( \left[ 0.3828,0.5075 \right] \right) } \end{array} \right) $	$ \left( \begin{array}{@{}c@{}} \displaystyle \left[ 0.5075,0.6012 \right] {e}^{i2\pi \left( \left[ 0.6789,0.7464 \right] \right) },\\ \displaystyle \left[ 0.0048,0.0494 \right] {e}^{i2\pi \left( \left[ 0.0494,0.0973 \right] \right) } \end{array} \right) $
$\overline{\overline{{\mathfrak{C}}_{{C}_{5}}}}$	$ \left( \begin{array}{@{}c@{}} \displaystyle \left[ 0.2048,0.2665 \right] {e}^{i2\pi \left( \left[ 0.2048,0.2665 \right] \right) },\\ \displaystyle \left[ 0.1029,0.3828 \right] {e}^{i2\pi \left( \left[ 0.1029,0.3828 \right] \right) } \end{array} \right) $	$ \left( \begin{array}{@{}c@{}} \displaystyle \left[ 0.6789,0.7464 \right] {e}^{i2\pi \left( \left[ 0.6789,0.7464 \right] \right) },\\ \displaystyle \left[ 0.0048,0.0494 \right] {e}^{i2\pi \left( \left[ 0.0048,0.0494 \right] \right) } \end{array} \right) $

Stage 4: Here, we compute the score values of the aggregated information in Stage 3, see Table 4.

**Table 4:** Score values.

	***CIV IFAAWA operator***	***CIV IFAAWG operator***
$\overline{\overline{{\mathfrak{C}}_{{C}_{1}}}}$	−0.3909	0.5263
$\overline{\overline{{\mathfrak{C}}_{{C}_{2}}}}$	−0.1409	0.4143
$\overline{\overline{{\mathfrak{C}}_{{C}_{3}}}}$	−0.4427	0.4289
$\overline{\overline{{\mathfrak{C}}_{{C}_{4}}}}$	−0.1646	0.5833
$\overline{\overline{{\mathfrak{C}}_{{C}_{5}}}}$	−0.0072	0.6855

Stage 5: Obtain the ranking information based on the score values, the detailed result is stated in Table 5.

**Table 5:** Ranking lists.

CIFAAWA operator	$\overline{\overline{{\mathfrak{C}}_{{C}_{5}}}}\gt \overline{\overline{{\mathfrak{C}}_{{C}_{4}}}}\gt \overline{\overline{{\mathfrak{C}}_{{C}_{1}}}}\gt \overline{\overline{{\mathfrak{C}}_{{C}_{2}}}}\gt \overline{\overline{{\mathfrak{C}}_{{C}_{3}}}}$
CIFAAWG operator	$\overline{\overline{{\mathfrak{C}}_{{C}_{5}}}}\gt \overline{\overline{{\mathfrak{C}}_{{C}_{2}}}}\gt \overline{\overline{{\mathfrak{C}}_{{C}_{4}}}}\gt \overline{\overline{{\mathfrak{C}}_{{C}_{1}}}}\gt \overline{\overline{{\mathfrak{C}}_{{C}_{3}}}}$

According to the theory of CIFAAWA operator and CIFAAWG operator, the best optimal is $\overline{\overline{{\mathfrak{C}}_{{C}_{5}}}}$.

### Influence of parameter

Here, we discuss the stability and influence of the derived operators based on the different values of parameters *ψ*. Therefore, by using the information in Table 2 and various parameter values, we obtain the subsequent consequence listed in Table 6.

**Table 6:** Represented the stability of the proposed work.

***Parameter***	***Operator***	***Score values***	***Ranking values***
*ψ* = 1	CIVIFAAWA operator	−0.3909, − 0.1409, − 0.4427, − 0.1646, − 0.0072	$\overline{\overline{{\mathfrak{C}}_{{C}_{5}}}}\gt \overline{\overline{{\mathfrak{C}}_{{C}_{4}}}}\gt \overline{\overline{{\mathfrak{C}}_{{C}_{1}}}}\gt \overline{\overline{{\mathfrak{C}}_{{C}_{2}}}}\gt \overline{\overline{{\mathfrak{C}}_{{C}_{3}}}}$
	CIVIFAAWG operator	0.5263, 0.4143, 0.4289, 0.5833, 0.6855	$\overline{\overline{{\mathfrak{C}}_{{C}_{5}}}}\gt \overline{\overline{{\mathfrak{C}}_{{C}_{2}}}}\gt \overline{\overline{{\mathfrak{C}}_{{C}_{4}}}}\gt \overline{\overline{{\mathfrak{C}}_{{C}_{1}}}}\gt \overline{\overline{{\mathfrak{C}}_{{C}_{3}}}}$
*ψ* = 5	CIVIFAAWA operator	−0.3901, − 0.1261, − 0.4416, − 0.1571, 0.0065	$\overline{\overline{{\mathfrak{C}}_{{C}_{5}}}}\gt \overline{\overline{{\mathfrak{C}}_{{C}_{4}}}}\gt \overline{\overline{{\mathfrak{C}}_{{C}_{1}}}}\gt \overline{\overline{{\mathfrak{C}}_{{C}_{2}}}}\gt \overline{\overline{{\mathfrak{C}}_{{C}_{3}}}}$
	CIVIFAAWG operator	0.5255, 0.4049, 0.4278, 0.5811, 0.6826	$\overline{\overline{{\mathfrak{C}}_{{C}_{5}}}}\gt \overline{\overline{{\mathfrak{C}}_{{C}_{2}}}}\gt \overline{\overline{{\mathfrak{C}}_{{C}_{4}}}}\gt \overline{\overline{{\mathfrak{C}}_{{C}_{1}}}}\gt \overline{\overline{{\mathfrak{C}}_{{C}_{3}}}}$
*ψ* = 11	CIVIFAAWA operator	−0.3888, − 0.1169, − 0.4401, − 0.1518, 0.0151	$\overline{\overline{{\mathfrak{C}}_{{C}_{5}}}}\gt \overline{\overline{{\mathfrak{C}}_{{C}_{4}}}}\gt \overline{\overline{{\mathfrak{C}}_{{C}_{1}}}}\gt \overline{\overline{{\mathfrak{C}}_{{C}_{2}}}}\gt \overline{\overline{{\mathfrak{C}}_{{C}_{3}}}}$
	CIVIFAAWG operator	0.5242, 0.3989, 0.4262, 0.5791, 0.6803	$\overline{\overline{{\mathfrak{C}}_{{C}_{5}}}}\gt \overline{\overline{{\mathfrak{C}}_{{C}_{2}}}}\gt \overline{\overline{{\mathfrak{C}}_{{C}_{4}}}}\gt \overline{\overline{{\mathfrak{C}}_{{C}_{1}}}}\gt \overline{\overline{{\mathfrak{C}}_{{C}_{3}}}}$
*ψ* = 51	CIVIFAAWA operator	−0.3818, − 0.1049, − 0.4329, − 0.141, 0.0274	$\overline{\overline{{\mathfrak{C}}_{{C}_{5}}}}\gt \overline{\overline{{\mathfrak{C}}_{{C}_{4}}}}\gt \overline{\overline{{\mathfrak{C}}_{{C}_{1}}}}\gt \overline{\overline{{\mathfrak{C}}_{{C}_{2}}}}\gt \overline{\overline{{\mathfrak{C}}_{{C}_{3}}}}$
	CIVIFAAWG operator	0.5176, 0.3901, 0.4185, 0.5724, 0.6748	$\overline{\overline{{\mathfrak{C}}_{{C}_{5}}}}\gt \overline{\overline{{\mathfrak{C}}_{{C}_{2}}}}\gt \overline{\overline{{\mathfrak{C}}_{{C}_{4}}}}\gt \overline{\overline{{\mathfrak{C}}_{{C}_{1}}}}\gt \overline{\overline{{\mathfrak{C}}_{{C}_{3}}}}$
*ψ* = 101	CIVIFAAWA operator	−0.3773, − 0.1013, − 0.4289, − 0.1363, 0.0323	$\overline{\overline{{\mathfrak{C}}_{{C}_{5}}}}\gt \overline{\overline{{\mathfrak{C}}_{{C}_{4}}}}\gt \overline{\overline{{\mathfrak{C}}_{{C}_{1}}}}\gt \overline{\overline{{\mathfrak{C}}_{{C}_{2}}}}\gt \overline{\overline{{\mathfrak{C}}_{{C}_{3}}}}$
	CIVIFAAWG operator	0.5138, 0.3876, 0.4142, 0.5696, 0.6726	$\overline{\overline{{\mathfrak{C}}_{{C}_{5}}}}\gt \overline{\overline{{\mathfrak{C}}_{{C}_{2}}}}\gt \overline{\overline{{\mathfrak{C}}_{{C}_{4}}}}\gt \overline{\overline{{\mathfrak{C}}_{{C}_{1}}}}\gt \overline{\overline{{\mathfrak{C}}_{{C}_{3}}}}$

We have gotten the consistent advantageous ideal $\overline{\overline{{\mathfrak{C}}_{{C}_{5}}}}$ based on diverse operators by utilizing the particular upsides of the boundary. This result shows that our calculation model has a good stability.

### Comparative analysis

Here, our main theme to evaluate the comparison between proposed method with few existing analyses to show the stability and effectiveness of the proposed method. For this, we use various existing operators such as Aggregation operators (AOs) (Xu, 2007), geometric AOs (Xu & Yager, 2006), information AOs (Wang & Liu, 2012), Einstein geometric AOs (Wang & Liu, 2012), Hamacher AOs (Huang, 2014), Dombi AOs (Seikh & Mandal, 2021) under the IFSs, and AOs Garg & Rani (2019a) and Garg & Rani (2019b) based on CIVIFSs. The comparative analysis is stated in Table 7 for the data in Table 2.

**Table 7:** Comparison information for Table 1.

Methods	Score values	Ranking values
Xu (2007)	× × × × × × × × × ×	× × × × × × × × × ×
Xu & Yager (2006)	× × × × × × × × × ×	× × × × × × × × × ×
Wang & Liu (2012)	× × × × × × × × × ×	× × × × × × × × × ×
Wang & Liu (2011)	× × × × × × × × × ×	× × × × × × × × × ×
Huang (2014)	× × × × × × × × × ×	× × × × × × × × × ×
Seikh and Mandal (2021)	× × × × × × × × × ×	× × × × × × × × × ×
Garg & Rani (2019a) and Garg & Rani (2019b)	0.4174, 0.3054, 0.3199, 0.4744, 0.5766	$\overline{\overline{{\mathfrak{C}}_{{C}_{5}}}}\gt \overline{\overline{{\mathfrak{C}}_{{C}_{2}}}}\gt \overline{\overline{{\mathfrak{C}}_{{C}_{4}}}}\gt \overline{\overline{{\mathfrak{C}}_{{C}_{1}}}}\gt \overline{\overline{{\mathfrak{C}}_{{C}_{3}}}}$
CIVIFAAWA operator	−0.3909, − 0.1409, − 0.4427, − 0.1646, − 0.0072	$\overline{\overline{{\mathfrak{C}}_{{C}_{5}}}}\gt \overline{\overline{{\mathfrak{C}}_{{C}_{4}}}}\gt \overline{\overline{{\mathfrak{C}}_{{C}_{1}}}}\gt \overline{\overline{{\mathfrak{C}}_{{C}_{2}}}}\gt \overline{\overline{{\mathfrak{C}}_{{C}_{3}}}}$
CIVIFAAWG operator	0.5263, 0.4143, 0.4289, 0.5833, 0.6855	$\overline{\overline{{\mathfrak{C}}_{{C}_{5}}}}\gt \overline{\overline{{\mathfrak{C}}_{{C}_{2}}}}\gt \overline{\overline{{\mathfrak{C}}_{{C}_{4}}}}\gt \overline{\overline{{\mathfrak{C}}_{{C}_{1}}}}\gt \overline{\overline{{\mathfrak{C}}_{{C}_{3}}}}$

**Notes.**

“ ×” denotes it is unsuitble to calculate the score values.

Under the various kinds of operators, we have gotten a completely consistent optimal judgment $\overline{\overline{{\mathfrak{C}}_{{C}_{5}}}}$ by utilizing the particular upsides of the boundary. The best optimal is $\overline{\overline{{\mathfrak{C}}_{{C}_{5}}}}$ according to the theory which was proposed by Garg & Rani (2019a) and Garg & Rani (2019b) based on CIVIFSs and proposed operators. Further, the derived theory of Aggregation operators (AOs) (Xu, 2007), geometric AOs (Xu & Yager, 2006), information AOs (Wang & Liu, 2012), Einstein geometric AOs (Wang & Liu, 2012), Hamacher AOs (Huang, 2014), Dombi AOs (Seikh & Mandal, 2021) under the IFSs have been failed, due to various limitations, because these operators or information ware proposed based on FSs, IFSs, IVIFSs, CFSs, and CIFSs which are the particular cases of the proposed information and hence they are not able to evaluate our suggestion information (CIVIF values).

Therefore, it can be inferred that the presented information and MADM model are very valuable and dominant for handling awkward information.

## Conclusion

In this manuscript, we combined four main theories such as CIVIF information, Aczel-Alsina operational laws, averaging/geometric aggregation operators, and the WASPAS technique. Furthermore, the theory of CIVIF information is the modified version of the FSs, IFSs, CFSs, CIFSs, and IVIFSs, because these are the special cases of the invented theory. Further, we derived the theory of aggregation operators based on Aczel-Alsina operational laws for CIVIF information. The theory of the WAPSAS technique is also proposed based on Aczel-Alsina aggregation operators for CIVIF information. The key influence of this assessment is debated below: (1) We initiated the Aczel-Alsina operational laws and their related results; (2) We initiated the principle of CIVIFAAWA, CIVIFAAOWA, CIVIFAAHA, CIVIFAAWG, CIVIFAAOWG, and CIVIFAAHG operators, and illustrated their well-known properties and results; (3) We derived the WASPAS method for CIVIFSs and evaluated their main steps with the help of some numerical examples; (4) We demonstrated the MADM strategy under the invented works; (5) We expressed the supremacy and dominancy of the invented works with the help of sensitive analysis and geometrical shown of the explored works.

In the future, we concentrate to derive some new ideas such as complex fuzzy superior Mandelbrot sets, complex intuitionistic fuzzy mandelbrot set, and their extension, and we try to utilize them in the field of artificial intelligence, machine learning, game theory, neural networks, and clustering analysis to improve or enhance the quality of the presented information.

## Additional Information and Declarations

### Data Availability

The following information was supplied regarding data availability: No raw data or code are used in the paper.
